# Identification of New Compounds with Anticonvulsant and Antinociceptive Properties in a Group of 3-substituted (2,5-dioxo-pyrrolidin-1-yl)(phenyl)-Acetamides

**DOI:** 10.3390/ijms222313092

**Published:** 2021-12-03

**Authors:** Michał Abram, Marcin Jakubiec, Anna Rapacz, Szczepan Mogilski, Gniewomir Latacz, Bartłomiej Szulczyk, Małgorzata Szafarz, Katarzyna Socała, Dorota Nieoczym, Elżbieta Wyska, Piotr Wlaź, Rafał M. Kamiński, Krzysztof Kamiński

**Affiliations:** 1 Department of Medicinal Chemistry, Faculty of Pharmacy, Jagiellonian University Medical College, Medyczna 9, 30-688 Krakow, Poland; michal.abram@uj.edu.pl (M.A.); marcin.jakubiec@doctoral.uj.edu.pl (M.J.); rafa1.kaminski@uj.edu.pl (R.M.K.); 2 Department of Pharmacodynamics, Faculty of Pharmacy, Jagiellonian University Medical College, Medyczna 9, 30-688 Krakow, Poland; a.rapacz@uj.edu.pl (A.R.); szczepan.mogilski@uj.edu.pl (S.M.); 3 Department of Technology and Biotechnology of Drugs, Faculty of Pharmacy, Jagiellonian University Medical College, Medyczna 9, 30-688 Krakow, Poland; gniewomir.latacz@uj.edu.pl; 4 Department of Pharmacodynamics, Centre for Preclinical Research and Technology, Medical University of Warsaw, Banacha 1B, 02-097 Warsaw, Poland; bartlomiej.szulczyk@wum.edu.pl; 5 Department of Pharmacokinetics and Physical Pharmacy, Faculty of Pharmacy, Jagiellonian University Medical College, Medyczna 9, 30-688 Krakow, Poland; malgorzata.szafarz@uj.edu.pl (M.S.); mfwyska@cyf-kr.edu.pl (E.W.); 6 Department of Animal Physiology and Pharmacology, Institute of Biological Sciences, Maria Curie-Skłodowska University, Akademicka 19, 20-033 Lublin, Poland; katarzyna.socala@mail.umcs.pl (K.S.); dorota.nieoczym@mail.umcs.pl (D.N.); piotr.wlaz@mail.umcs.pl (P.W.)

**Keywords:** pyrrolidine-2,5-dione, salts, hybrid compounds, anticonvulsant activity, antinociceptive activity, electrophysiology, ADME-Tox studies

## Abstract

We report herein a series of water-soluble analogues of previously described anticonvulsants and their detailed in vivo and in vitro characterization. The majority of these compounds demonstrated broad-spectrum anticonvulsant properties in animal seizure models, including the maximal electroshock (MES) test, the pentylenetetrazole-induced seizure model (*sc*PTZ), and the psychomotor 6 Hz (32 mA) seizure model in mice. Compound **14** showed the most robust anticonvulsant activity (ED_50_ MES = 49.6 mg/kg, ED_50_ 6 Hz (32 mA) = 31.3 mg/kg, ED_50_ *sc*PTZ = 67.4 mg/kg). Notably, it was also effective in the 6 Hz (44 mA) model of drug-resistant epilepsy (ED_50_ = 63.2 mg/kg). Apart from favorable anticonvulsant properties, compound **14** revealed a high efficacy against pain responses in the formalin-induced tonic pain, the capsaicin-induced neurogenic pain, as well as in the oxaliplatin-induced neuropathic pain in mice. Moreover, compound **14** showed distinct anti-inflammatory activity in the model of carrageenan-induced aseptic inflammation. The mechanism of action of compound **14** is likely complex and may result from the inhibition of peripheral and central sodium and calcium currents, as well as the TRPV1 receptor antagonism as observed in the in vitro studies. This lead compound also revealed beneficial in vitro ADME-Tox properties and an in vivo pharmacokinetic profile, making it a potential candidate for future preclinical development. Interestingly, the in vitro studies also showed a favorable induction effect of compound **14** on the viability of neuroblastoma SH-SY5Y cells.

## 1. Introduction

Water solubility is one of the essential physicochemical properties from the perspective of formulation development for orally or parenterally (especially intravenously) administrated drug candidates [1]. Thus, it is a key driver for drug absorption, achieving a desired therapeutic exposure in plasma and target issues required for an effective pharmacological response. In consequence, a low aqueous solubility is one of the most essential problems related with the formulation preparation of new chemical entities and may cause the failure of a drug candidate in advanced preclinical or clinical development [2]. It should be stressed here that more than 40% of the currently marketed drugs and up to 75% of compounds under development are practically insoluble [3,4]. Bearing in mind the aforementioned facts, one of the important directions of lead compound optimization involves the improvement of its water solubility, which may be achieved by both physical and chemical methods. The latter relies primarily on the direct conversion of a molecule to a salt or introducing certain functional substituents to the parent structure (e.g., amine or carboxylic groups) that enable the formation of such salt bridges, as well as the insertion of non-ionized polar moieties (e.g., hydroxyl group), which may lead to a better solubility in water. However, the major challenge of any interference with the structure of a lead compound is the improvement of the solubility while maintaining or enhancing its biological activity. The physical approaches involve, among others, crystal engineering, particle size reduction, solid dispersion, the addition of surfactants, complexation, etc. [5].

**Figure 1:**
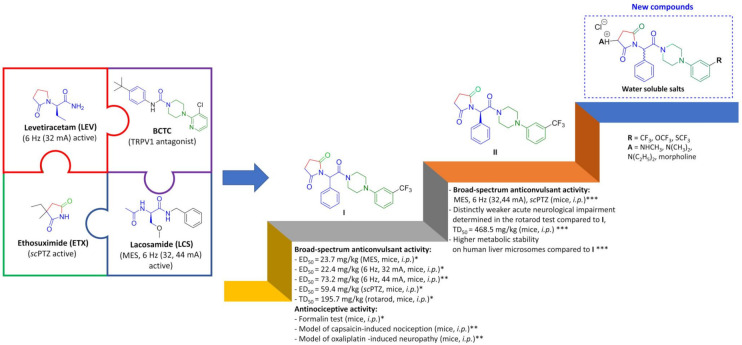
Molecular hybridization-yielding model, broad-spectrum anticonvulsants **I** and **II**, and general structure of new water-soluble congeners. * Data from ref. [6] (see compound **22**), ** Data from ref. [7] (see compound **KA-104**), *** Data from ref. [8] (see compound **(*R*)-16**).

Our most recent studies led to the identification of several hybrid compounds belonging to a group of pyrrolidine-2,5-dione derivatives, which proved to be promising candidates for further preclinical development in epilepsy and neuropathic pain indications [6,7,8]. It should be mentioned here that succinimide derivatives have been explored by many research groups in the CNS area, i.e., for the identification of new anticonvulsant and antidepressant agents [9,10,11]. Particularly, compounds I and II shown in Figure 1 revealed beneficial anticonvulsant properties. Both the aforementioned substances were found to be effective in all commonly employed screening seizure models with potent protection in the maximal electroshock seizure test (MES), the pentylenetetrazole seizure test (*sc*PTZ), as well as the 6 Hz seizure model (32 and 44 mA) in mice. Importantly, compound II (*R*-enantiomer) vs. I (racemate) was characterized by a distinctly more beneficial safety profile in the rotarod test, a higher metabolic stability on human liver microsomes (HLMs), as well as a less pronounced inhibition of CYP2C9 [8]. Notably, both I and II (data not published till now for II) demonstrated a potent effectiveness by decreasing pain responses in the capsaicin-induced pain, the formalin-induced tonic pain, and the oxaliplatin (OXPT)-induced neuropathic pain models in mice [7].

Despite the beneficial pharmacological properties of I and II, as well as their close analogues described before [12,13,14,15], the main drawback of these molecules is the low water solubility, hampering the preparation of optimal oral formulations. To address this problem, we designed and synthesized herein a series of water-soluble hydrochlorides, which are obtained by the introduction of an amine moiety into pyrrolidine-2,5-dione ring of previously described anticonvulsants [6,7,8]. It should be stressed that such compounds with an improved water solubility may also facilitate the development of intravenous formulations used in emergency clinical conditions, e.g., status epilepticus. The design strategy (molecular hybridization) yielding broad-spectrum anticonvulsants I and II, and the general structure of the compounds obtained herein are shown in Figure 1.

All substances obtained in the current studies were tested in vivo for their anticonvulsant activity in the MES, *sc*PTZ, and 6 Hz (32 mA) seizure models. In addition, for the most promising anticonvulsant, its efficacy in the 6 Hz (44 mA) seizure model of drug resistant epilepsy, an influence on the seizure threshold in the *iv*PTZ test, as well the as anti-nociceptive and anti-inflammatory activity were evaluated. Additionally, as part of the safety profiling for the lead molecule, we determined its influence on motor coordination in the rotarod test, neuromuscular strength, and rectal temperature in mice. We also assessed its in vivo pharmacokinetic profile and several in vitro ADME-Tox parameters, such as the hepatotoxicity, neurotoxicity, and influence on the function of several cytochrome P-450 isoforms (CYP3A4 and CYP2D6), to support the early development of a potential new drug candidate. Finally, we attempted to elucidate the possible mechanisms of action in the binding and functional (e.g., patch-clamp electrophysiological) studies.

## 2. Results and Discussion

### 2.1. Chemistry

**Scheme 1:**
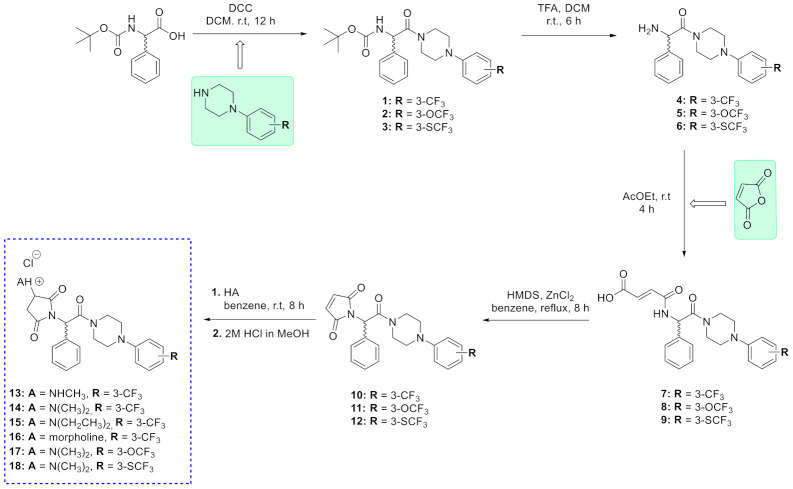
Synthesis of intermediates **1**–**12** and target water-soluble salts **13**–1**8**.

The (hydrochlorides) **13**–**18** were prepared according to the method depicted in Scheme 1. In the first step, the *N*-(*tert*-butoxycarbonyl)-DL-phenylglycine was coupled in the presence of DCC (*N*,*N*′-dicyclohexylcarbodiimide) with an appropriate phenylpiperazine to give Boc-protected phenylglycine derivatives **1**–**3**. The removal of the Boc-protecting group in the presence of trifluoroacetic acid (TFA) yielded amines **4**–**6**. Next, amines **4**–**6** were converted to amido-acid (maleamic) derivatives **7**–**9** in the condensation reaction with maleic anhydride. The monounsaturated pyrrolidine-2,5-dione derivatives **10**–**12** were obtained applying the hexamethyldisilazane (HMDS)-promoted cyclization reaction of derivatives **7**–**9**. The final compounds were obtained in the addition reaction of the primary or secondary amine with derivatives **10**–**12**. This reaction was performed in benzene at room temperature. Finally, the water-soluble salts **13**–**18** were converted into water-soluble hydrochlorides by treating the compound with a 2M methanolic hydrochloric acid solution. The desired compounds were obtained as white solids, followed by the concentration of organic solvents under reduced pressure and wash-up with diethyl ether.

**Scheme 2:**
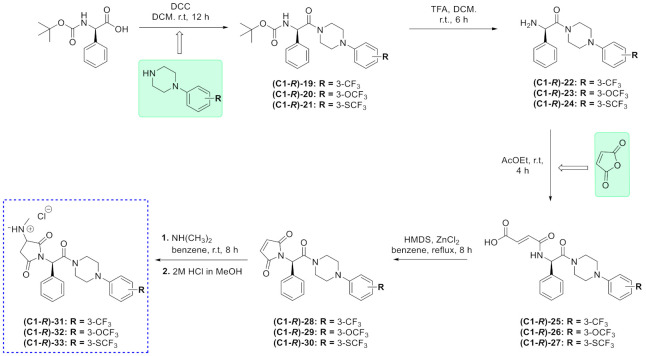
Synthesis of intermediates **(C1**-***R*)**-**19**–**30** and target hydrochlorides **(C1-*R*)-31**–**33**.

The next step of structural modifications involved the synthesis of compounds with an *R*-configuration of the asymmetric carbon atom (C1*-R*) located in the phenylacetamide moiety. Notably, this series was restricted only to three diastereoisomers **(C1-*R*)-31**–**33**, which were analogues of the most potent anticonvulsants with the *R*-configuration reported previously [8]. Furthermore, as an amine function in position three of the pyrrolidine-2,5-dione, we introduced the dimethylamine moiety that appeared to be especially beneficial for anticonvulsant activity among compounds **13**–**18**. The synthetic procedure was similar to racemates **13**–**18** and the commercially available (*R*)-2-((*tert*-butoxycarbonyl)amino)-2-phenylacetic acid was used as a starting material (for details, see Scheme 2). As the final compounds, (**C1-*R*)-31**–**33** were diastereoisomers generated by the introduction of amine at the C3 carbon atom of the imide ring (*R*,*S* configuration); the assessment of the enantiomeric purity at the **C1** carbon atom was determined for the monounsaturated pyrrolidine-2,5-dione derivatives **(C1-*R*)-28**–**30** and was >99% as determined by the chiral HPLC analysis. Furthermore, to check the retention of the configuration of the **C1** carbon atom at the phenyl-acetamide fragment and to exclude its potential racemization in the basic environment resulting from the addition of dimethylamine, we also performed a chiral resolution for the final hydrochlorides **(C1-*R*)-31**–**33**. The chiral HPLC results proved that the synthetic procedure applied enabled to retain the predefined configuration of the **C1** carbon atom of the phenylglycine fragment. The chiral HPLC chromatograms for the unsaturated enantiomers **(C1-*R*)-28**–**30** and the final diastereoisomers **(C1-*R*)-31**–**33** are shown in Supplementary Materials.

In the next step of the chemical studies, with the aim of confirming the preferential stereochemistry of the C1 carbon atom at the phenylacetamide linker for the most promising anticonvulsant identified among the racemates, compound **14** (for in vivo data, see Table 1), its **C1**-*S*-enantiomer (**(C1-*S*)-31**) was synthesized by applying a similar procedure as for the *R*-stereoisomers. In this case, the (*S*)-2-((*tert*-butoxycarbonyl)amino)-2-phenylacetic acid was used as the starting material (for details, see Scheme S1).

The target hydrochlorides were obtained in good yields (>70%). The structures of the final molecules were confirmed by ^1^H NMR, ^13^C NMR, and LC-MS spectra. The ^1^H NMR and ^13^C NMR spectra were prepared for free bases with the aim of a better signals visualization. An elemental analysis (C, H, and N) was performed for all final compounds (these data confirmed the formation of monohydrochlorides). The purity of target compounds determined by the use of the chromatographic UPLC method was ≥99%.

### 2.2. Anticonvulsant Activity

The preclinical animal seizure models, such as the MES, 6 Hz (32 mA or 44 mA), and *sc*PTZ tests, remain a routine tool in the search for new antiseizure drugs (ASDs) in view of the high predictability of the assessment of the potential clinical efficacy of candidates for new anticonvulsants [16]. Therefore, the efficacy of all ASDs introduced into treatment till now were confirmed applying the screening approaches in different animal seizure models. It should be stressed that the MES test is still recognized as one of the most useful preclinical seizure models as it enables the identification of substances potentially effective in the tonic–clonic seizures and partial convulsions with or without secondary generalization in humans [17]. In consequence, all final compounds were initially tested in the MES model after an intraperitoneal (*i.p*.) administration at a screening dose of 100 mg/kg in mice (in a group consisting of four animals) and the protection against MES seizures was observed at the pretreatment time point of 0.5 h, similarly to our previous studies [6,8].

According to the results obtained in the MES test, at least 75% of protection (three mice protected out of four tested) was demonstrated for compounds **14**, **15**, **17**, **(C1-*R*)-31** and **(C1-*R*)-32** (Table S1). Notably, 3-CF_3_ (**14**, **15**, **(C1-*R*)-31**) and 3-OCF_3_ (**(C1-*R*)-32**) derivatives provided maximal (100%) protection. Other substances showed weak (25%) or no activity at a dose of 100 mg/kg. An equally important, well-established, and commonly used preclinical seizure model in the discovery of new ASDs effective in human focal epilepsy is the psychomotor 6 Hz (32 mA) test [18]. Therefore, in the next step of the pharmacological characterization, all final compounds obtained were studied in this seizure model (data obtained are listed in Table S1). As a result, compounds **14**, **15**, **(C1-*R*)-31**, and **(C1-*R*)-32** displayed a potent anticonvulsant efficacy providing 75% protection, whereas **13** and **17** protected 50% of the animals. The further pharmacological investigations were focused on the efficacy assessment in the *sc*PTZ seizure model, which is known to be an animal model of general absence epilepsy [19]. In this test, **17**, **(C1-*R*)-31**, and **(C1-*R*)-32**) were the most potent compounds and displayed 75% protection at the time point of 0.5 h. Importantly, the detailed in vivo characterization of racemate **14** and its C1 stereoisomers revealed a distinctly weaker protection in all seizure models for compound **(C1-*S*)-31**, with the *S* configuration of the aforementioned carbon atom vs. **14** and **(C1-*R*)-31** diastereoisomer. These observations were in line with data obtained for its close analogues described previously [8,13].

In summary, based on the above screening data, four compounds with the dimethylamine group at the pyrrolidine-2,5-dione ring and 3-CF_3_ ((**14**, **(C1-*R*)-31**) or 3-OCF_3_ ((**17**, **(C1-*R*)-32**) substituents at the phenylpiperazine analogues revealed broad-spectrum anti-convulsant activity and potent protection in the MES, *sc*PTZ, and 6 Hz (32 mA) seizure models. The replacement of the dimethylamine group attached to the succinimide ring into diethylamine (**15**) yielded a compound that was predominantly active in the MES and 6 Hz (32 mA) tests. All other structural modifications of this place such as the introduction of methylamine (**13**) or morpholine (**16**), the insertion of the trifluoromethyl-thio group (**18**, **(C1**-***R*)**-**33**) at the *meta*-position of the phenylpiperazine moiety in place of trifluoromethyl or trifluoromethoxy substituents, as well as the inversion of the absolute configuration of the C1 carbon atom located in the phenylacetamide linker (from *R* to *S*), decreased the anticonvulsant activity (or caused the compounds to become inactive).

In the next step, based on the above screening data, the median effective doses (ED_50_) in the MES, 6 Hz (32 mA), and *sc*PTZ tests and the median neurotoxic doses (TD_50_) in the rotarod test were determined for the most effective compounds (at least 50% of protection in the screening studies, see Table S1). The protective indexes (PI) which describe the benefit–risk ratio of the therapeutic agent were calculated for each seizure model (PI = TD_50_/ED_50_).

The quantitative data summarized in Table 1 showed that compound **14** (3-CF_3_ derivative), **17** (3-OCF_3_ analogue), and their *R* stereoisomers at the phenylglycine fragment **(C1-*R*)-31** and **(C1-*R*)-32**, respectively, were characterized by the most potent anticonvulsant properties. Notably, all of these compounds possessed the dimethylamino moiety at the three position of the pyrrolidine-2,5-dione that seems to be preferential for potent anticonvulsant activity in the series of hydrochlorides described herein. It should be emphasized that in vivo data did not reveal significant or clear differences in biological activity between the aforementioned racemates (**14**, **17**) and compounds with the *R* configuration in the phenylglycine fragment—**(C1-*R*)-31** and **(C1-*R*)-32**. Nevertheless, preferably, these molecules showed broad and potent anticonvulsant activity in the MES and 6 Hz (32 mA) test and, of note, there was a distinct improvement of activity in the *sc*PTZ seizure model for **17** and **(C1-*R*)-32** (both 3-OCF_3_ derivatives) compared to the parent and hardly soluble compounds reported previously (i.e., **I** and **II** in Table 1) [6,8]. Unfortunately, all compounds reported herein were characterized by a higher motor impairment in the rotarod test that resulted in a lower safety margin expressed as PIs in relation to their three unsubstituted succinimide precursors **I** and **II** obtained before [6,8]. The higher acute neurotoxicity (motor impairment) in the rotarod test may result from very a fast penetration to the murine brain and a much higher concentration of salts in the CNS compared to the parent and hardly water-soluble compounds as it was observed for compound **14** and its direct analogue **I** (see compound KA-104, ref. [7]), in the pharmacokinetic studies (pharmacokinetic data for compound **14** are shown below).

**Table 1:** The quantitative pharmacological parameters ED_50_, TD_50_, and PIs in mice *i.p*.

Compound	TPE (h) ^a^	ED_50_ MES (mg/kg) ^b^	ED_50_ 6 Hz (32 mA) (mg/kg) ^c^	ED_50_ *sc*PTZ (mg/kg) ^d^	TD_50_ (mg/kg) ^e^	PI (TD_50_/ED_50_) ^f^
**13**	0.5	-	93.6 (77.2–113.4)	85.6 (57.1–128.4)	160.1 (143.1–179.0	1.7 (6 Hz) 1.9 (*sc*PTZ)
**14**	0.5	**49.6** (44.3–55.7)	**31.3** (18.2–53.9)	**67.4** (58.2–92.1)	**168.7** (146.3–194.5)	**3.4** (MES) **5.4** (6 Hz) **1.4** (*sc*PTZ)
**15**	0.5	77.5 (73.8-81.4)	80.4 (70.5–91.7)	-	246.6 (214.9–282.9)	3.2 (MES) 1.8 (6 Hz)
**17**	0.5	33.3 (28.9–38.4)	28.2 (16.9–47.2)	31.3 (18.2–53.9)	69.7 (52.0–93.5)	1.6 (MES) 3.7 (6 Hz) 2.2 (*sc*PTZ)
**(C1-*R*)-** **31**	0.5	57.7 (33.9–97.9)	50.9 (45.3–57.1))	65.5 (47.9–89.6)	94.9 (75.3–119.4)	1.6 (MES) 1.9 (6 Hz) 1.5 (*sc*PTZ)
**(C1-*S*)-31**	0.5	-	>130	-	188.5 (181.3–195.9)	n.c.
**(C1-*R*)-32**	0.5	26.3 (18.9–36.8)	28.3 (20.0–40.1)	44.9 (37.2–54.3)	72.0 (64.5–80.4)	2.7 (MES) 2.5 (6 Hz) 1.6 (*sc*PTZ)
**I** *	0.5	23.7 (18.4–31.2)	22.4 (17.4–28.8)	59.4 (37.5–94.1)	195.7 (132.7–288.6)	8.2 (MES) 8.7 (6 Hz) 3.3 (scPTZ)
**II** **	0.5	36.0 (31.4–41.2)	39.2 (31.6–48.6)	54.8 (43.5–69.1)	468.5 (397.0–553.0)	13.0 (MES) 12.0 (6 Hz) 8.5 (scPTZ)
**ETX ^g^**	0.25	n.a.	>200	140.4 (115.8–170.2)	318.0 (295.8–341.9)	2.3 (*sc*PTZ)
**LCS ^g^**	0.5	9.2 (8.5–10.0)	5.3 (3.5–7.8)	n.a.	46.2 (44.5–48.0)	5.0 (MES) 8.8 (6 Hz)
**LEV ^g^**	1.0	>500	15.7 (10.4–23.7)	n.a.	>500	>31.8 (6 Hz)
**VPA ^g^**	0.5	252.7 (220.1–290.2)	130.6 (117.6–145.2)	239.4 (209.2–274.1)	430.7 (407.9–454.9)	1.7 (MES) 3.3 (6 Hz) 1.8 (*sc*PTZ)

The data for the lead compound are in bold for better visualization. Values in parentheses are 95% confidence intervals [20]. **^a^** Time to peak effect. **^b^** ED_50_ (MES—maximal electroshock seizure test). **^c^** ED_50_ (6 Hz seizure test, 32 mA). **^d^** ED_50_ (*sc*PTZ—subcutaneous pentylenetetrazole seizure test). **^e^** TD_50_ (NT-acute neurological deficit determined in the rotarod test). **^f^** Protective index (TD_50_/ED_50_). **^g^** Reference ASDs: ethosuximide (ETX), lacosamide (LCS), levetiracetam (LEV), and valproic acid (VPA) tested in the same conditions. TPEs for model ASDs taken from own experiments or literature [21]. * Data for racemate I, see compound **22** in ref. [6]. ** Data for (*R*)-enantiomer II, see compound **(*R*)-16** in ref. [8]. A dash—not tested; n.a.—non-active; n.c.—not-calculated.

A more detailed pharmacological characterization of compound **14**, which demonstrated the most beneficial anticonvulsant and safety profile, proved its activity in the 6 Hz (44 mA) test. It should be emphasized that the 6 Hz (44 mA) test is currently one of the most important screening models for the identification and characterization of novel compounds with a potential efficacy in pharmacoresistant epilepsy [22,23]. The quantitative data for compound **14**, chemical prototype **I** (published previously [7]), LEV, and LCS, as the most effective ASDs in the 6 Hz (32 mA) test, as well as VPA as the broad-spectrum ASD, are summarized in Table 2. 

The results obtained revealed a relatively high activity for compound **14** in the 6 Hz (44 mA) test. It should be stressed that compound **14** was more effective and displayed a better safety profile (PI value) than that of VPA, which is one of the most clinically relevant broad-spectrum ASDs [24]. It is noteworthy that in this seizure model, LEV, which binds to the SV2A protein located in the membranes of synaptic vesicles, was ineffective even at a very high dose of 500 mg/kg, while the most effective substance was LCS that enhances the slow inactivation of voltage-gated sodium channels [25].

In summary, the obtained in vivo data enabled to identify compound **14** as a potent and broad-spectrum anticonvulsant for future preclinical development (especially after oral administration). Notably, compound **14** showed distinctly more potent anticonvulsant activity in the MES, 6 Hz (32/44 mA), and *sc*PTZ models compared to the broad-spectrum ASD, that is, VPA. It should be emphasized that the key chemical modification performed in the current studies allowed to obtain water-soluble salts which were close analogues of hybrid anticonvulsants reported previously [6,7,8]. In general, this modification did not affect the potent and broad-spectrum activity in the MES, 6 Hz (32/44 mA), and *sc*PTZ seizure models, but, unfortunately, compounds reported herein were simultaneously characterized by a higher motor impairment in the rotarod test that resulted in a lower safety margin expressed as PIs in relation to their chemical precursors **I** and **II** (Table 1). Nevertheless, in our opinion, the further development of this group, and especially compound **14,** was undoubtedly necessary, especially after oral administration.

### 2.3. Effect on the Seizure Threshold in the ivPTZ Test in Mice

The timed *iv*PTZ test was employed to evaluate the acute effect of compound **14** on the seizure thresholds for (1) the first myoclonic twitch, (2) generalized clonic seizure with the loss of the righting reflex, and (3) forelimb tonus. The experimental procedure has been described in detail elsewhere [26].

**Figure 2:**
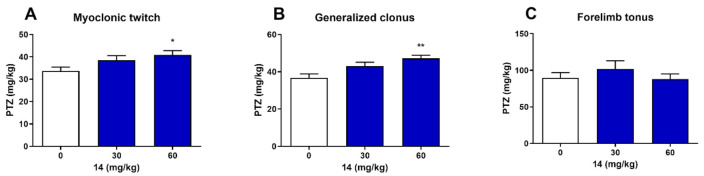
Acute effect of compound **14** on the threshold for the first myoclonic twitch (panel (**A**)), generalized clonus (panel (**B**)), and forelimb tonus (panel (**C**)) in the *iv*PTZ seizure threshold test in mice. Compound **14** was administered *i.p.* 30 min before the seizure test. Control animals received vehicle only. Each experimental group consisted of 9–11 animals. Each bar represents the mean (mg/kg PTZ) + S.E.M. * *p* < 0.05; ** *p* < 0.01 vs. the control group (one-way ANOVA, followed by Dunnett’s post hoc test, GraphPad Prism 8.0.1).

The acute effect of compound **14** on the seizure thresholds in the *iv*PTZ test is shown in Figure 2A–C (one-way ANOVA: F(2,28) = 3.58, *p* = 0.041, for myoclonic twitch; F(2,25) = 7.04, *p* = 0.003, for generalized clonus; F(2,26) = 0.72, *p* = 0.50, for forelimb tonus). Compound **14** administered at a dose of 30 mg/kg did not significantly affect the thresholds for the first myoclonic twitch and generalized clonus. However, when injected at a dose of 60 mg/kg, it slightly increased the thresholds for both the first myoclonic twitch and generalized clonic seizure (*p* < 0.05 and *p* < 0.01, respectively). No statistically significant changes in the threshold for forelimb tonic extension after compound **14** administration (at 30 and 60 mg/kg) were observed.

### 2.4. Neuromuscular Strength and Rectal Temperature

**Figure 3:**
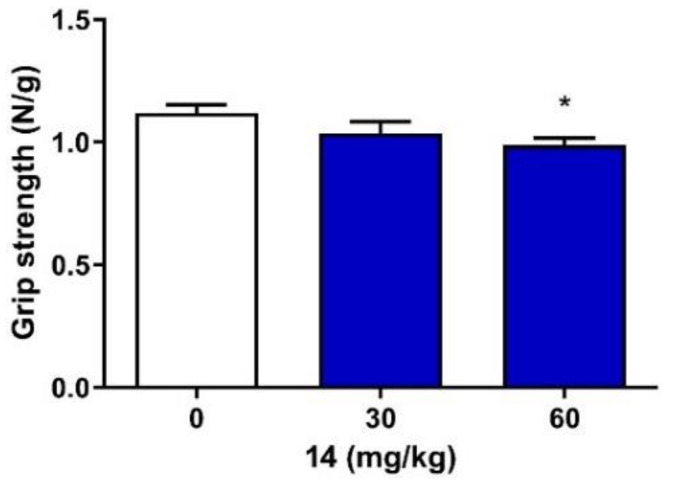
Acute effect of compound **14** on the neuromuscular strength in mice. Compound **14** was administered *i.p.* 30 min before the test. Control animals received vehicle only. Each experimental group consisted of 10 animals. Each bar represents the mean + S.E.M. grip strengths in Newtons per gram of mouse body weight (N/g). * *p* < 0.05 vs. the control group (one-way ANOVA, followed by Dunnett’s post hoc test, GraphPad Prism 8.0.1).

Although one-way ANOVA (F(2,27) = 3.28, *p* = 0.053) showed no statistically significant differences between group means, the Dunnett’s post hoc test revealed that compound **14** at a dose of 60 mg/kg significantly decreased the grip strength (*p* < 0.05). At a lower dose, i.e., 30 mg/kg, compound **14** did not significantly affect the neuromuscular strength in mice (Figure 3).

**Figure 4:**
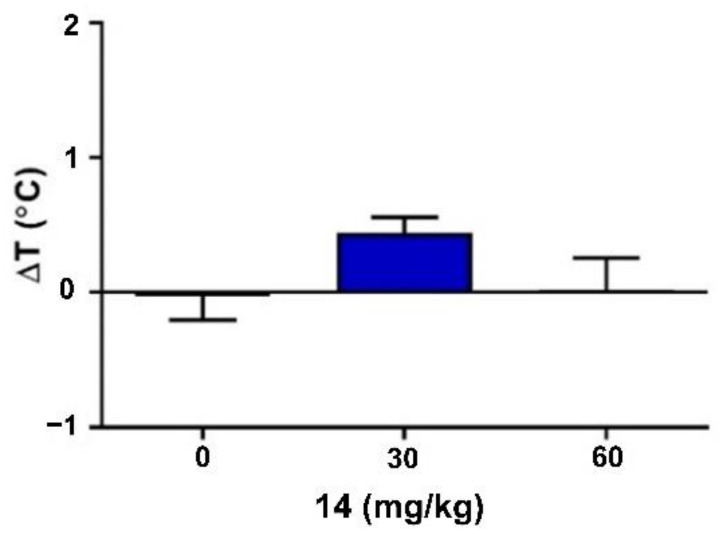
Acute effect of compound **14** on rectal temperature in mice. Compound **14** was administered *i.p.* 30 min before the seizure test. Control animals received vehicle only. Each experimental group consisted of 10 animals. Each bar represents the mean difference between the pre-injection and post-injection values of rectal temperature in mice (ΔT) + S.E.M. Data were analyzed with one-way ANOVA test (GraphPad Prism 8.0.1).

Compound **14** at doses of 30 and 60 mg/kg did not produce statistically significant changes in body temperature, as determined by the comparison of the differences between the pre-injection and post-injection values of rectal temperature in mice (one-way ANOVA: F(2,27) = 2.00, *p* = 0.155; Figure 4).

### 2.5. Antinociceptive Activity

Taking into account the fact that ASDs are most often used in the treatment of pain resulting from the damage/dysfunction of the central or peripheral nervous system, for the most potent anticonvulsant (**14**), the in vivo studies were conducted to confirm its antinociceptive activity in various animal models of pain.

The formalin test is a commonly used model of pain also useful in screening the analgesic activity of novel compounds. A subcutaneous formalin injection results in a biphasic nociceptive response; a phase one (5 min) followed by a quiescent period (10 min) and then phase two (15 min). C-fibers’ initial and direct stimulation in phase one led to peripheral inflammation accompanied by ongoing peripheral nerve activity and central sensitization during phase two. Moreover, a formalin injection induces persistent changes similar to those observed in nerve injury and subsequent neuropathic pain [27]. Our previous papers showed that the new anticonvulsant hybrid molecules with a multitarget mechanism of action have potent analgesic activity. These compounds showed efficacy in both phases of the formalin test, which was superior to ASDs such as VPA, showing an analgesic potential only in the second phase of the test [7,13].

**Figure 5:**
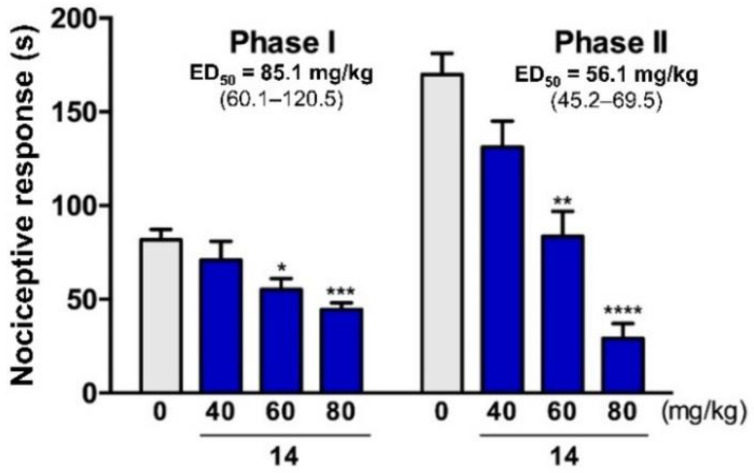
Antinociceptive activity of compound **14** in the formalin test. Results are shown as the nociceptive response time considered as licking or biting the paw injected with formalin. Phase I (0–5 min after intraplantar injection of formalin) and in phase II (15–30 min after formalin injection). Each value represents the mean ± S.E.M. for 8–10 animals. Statistical analysis: one-way ANOVA followed by post hoc Dunnett’s test. Statistical significance compared to vehicle-treated animals (Tween): * *p* < 0.05, ** *p* < 0.01, *** *p* < 0.001, **** *p* < 0.0001 (GraphPad Prism 8.0.1).

As a continuation of those works, we investigated the analgesic properties of the lead compound **14**. As shown in Figure 5, the *i.p.* administration of compound **14** before the subcutaneous injection of formalin significantly attenuated the nociceptive response in mice in both phases of the test. Its ED_50_ value in phase I was 85.1 mg/kg, whereas the ED_50_ value in phase II was 56.1 mg/kg. These results suggested promising analgesic properties of compound **14**, which were subsequently confirmed in other pain models.

**Figure 6:**
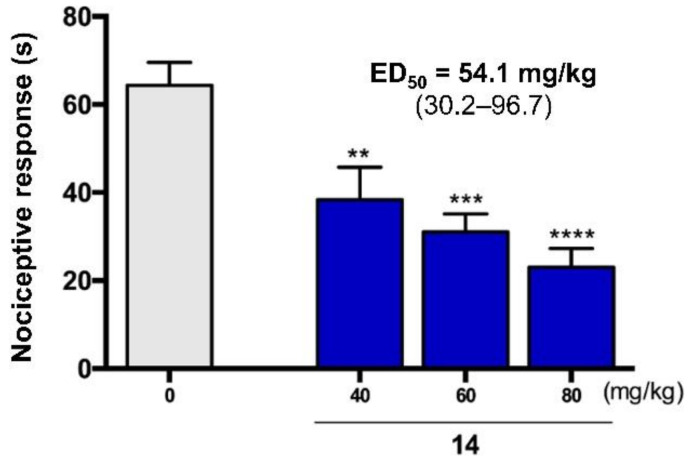
Antinociceptive activity of compound **14** in the capsaicin test. Results are shown as the time of nociceptive response (licking and biting the paw irritated with capsaicin) in 5 min period after intraplantar injection of capsaicin. Each value represents the mean ± S.E.M. for 8–10 animals. Statistical analysis: one-way ANOVA, followed by post hoc Dunnett’s test. Statistical significance compared to vehicle-treated animals (Tween): ** *p* < 0.01, *** *p* < 0.001, **** *p* < 0.0001 (GraphPad Prism 8.0.1).

We used the capsaicin-induced pain model to test the impact of compound **14** on neurogenic pain. In this model, the activation of TRPV1 receptors on C-type primary afferents led to the release of inflammatory neuropeptides, contributing to the local and spinal sensitization and excitability. We found that the duration of pain behaviors in the vehicle-treated mice was 64.36 ± 5.2 s. As shown in Figure 6, compound **14** attenuated the nociceptive response in a dose-dependent manner with an ED_50_ value of 54.1 mg/kg. These results showed that compound **14** attenuated the early neurogenic phase of nerve response for the noxious stimulus. It contrasted to VPA, which was active in this test only in a very high dose such as 200 mg/kg, as we showed in our previous paper [13].

**Figure 7:**
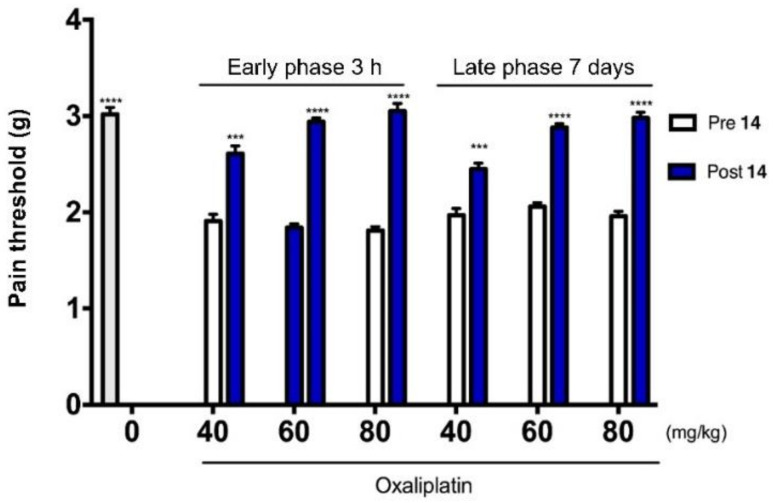
Antiallodynic effects of compound **14** in the tactile allodynia evaluated in the von Frey test in oxaliplatin-induced peripheral neuropathy. Statistical analysis: repeated measures analysis of variance (ANOVA), followed by Dunnett’s post hoc comparison: *** *p* < 0.001, **** *p* < 0.0001 (GraphPad Prism 8.0.1). Results obtained before the treatment with oxaliplatin (the gray bar) and after the treatment with oxaliplatin and the compound (post compound **14**, the red bars) were compared to the results obtained after the treatment with oxaliplatin and before the administration of the compound (pre compound **14**; the white bars).

To investigate the analgesic activity of compound **14** in neuropathic pain, we used the OXPT-induced peripheral neuropathy model. OXPT directly affects neurons, which results in the upregulation of TRPM8 and TRPA1 and the downregulation of Kv4.3 neuronal channels. Moreover, OXPT forms adduct with mitochondrial DNA and, in this way, change the expression of membrane-associated protein such as channels. All these effects finally lead to peripheral and central sensitization [28,29]. The neuropathy manifests as the decrease in the pain threshold and can be observed in hours or in days after OXPT administration, which is called the early or late phase of neuropathic pain, respectively. We tested the influence of compound **14** on tactile allodynia using the von Frey method 3 h and 7 days after the induction of neuropathy [30]. In the early phase, we observed a significant decrease in the value of mean force that caused a paw withdrawal reaction (1.84 ± 0.05 g) (60.9% of the baseline) compared to non-treated animals (3.02 ± 0.07 g) (the baseline). In the late phase, the pain threshold was 1.97 ± 0.07 (65.2% of the baseline). The observed tactile allodynia was attenuated by compound **14**, which elevated the pain sensitivity threshold in the early phase in a dose-dependent manner. (Figure 7) (2.61 ± 0.08 g, 2.94 ± 0.04 g, and 3.05 ± 0.08 g at the dose of 40 mg/kg, 60 mg/kg, and 80 mg/kg, respectively, which corresponded to 86.4%, 97.3%, and 100.9% of the baseline value). In the late phase, the pain threshold values were 2.45 ± 0.06 g, 2.88 ± 0.04 g, and 2.98 ± 0.06 g, which corresponded to 81.1%, 95.3%, and 98.6% of the baseline value. These results indicated that compound **14** has the potential for relieving, particularly neuropathic, pain (all the tested doses were significantly active) and should be further tested in that direction.

**Figure 8:**
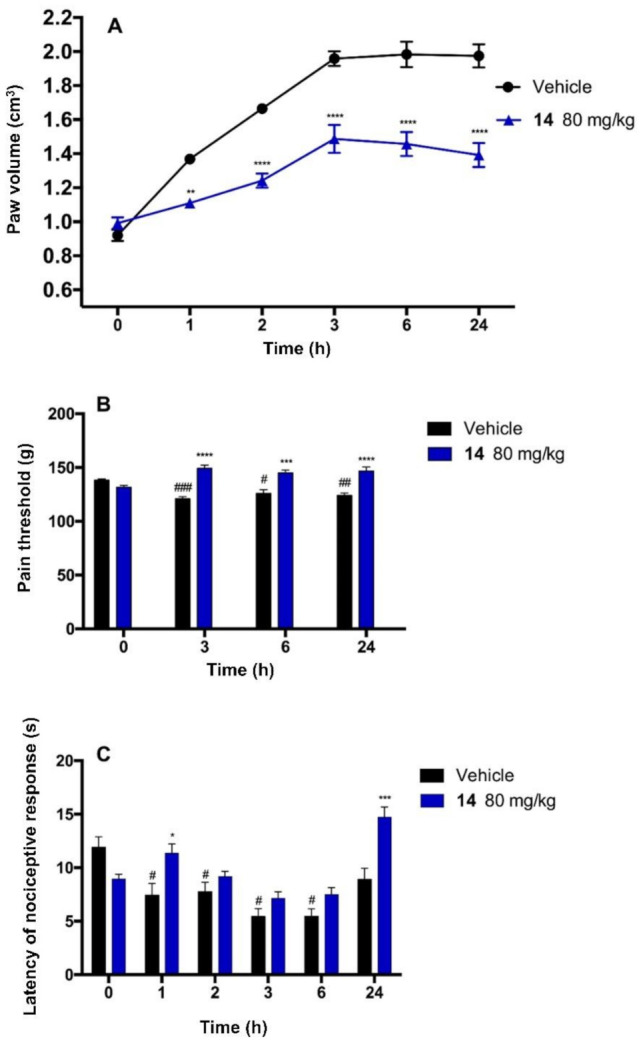
Effect of compound **14** on carrageenan-induced oedema (**A**), mechanical hyperalgesia (**B**), and thermal hyperalgesia (**C**), developed after subplantar injection of 1% carrageenan in rats. Data are expressed as means ± S.E.M. for 6 animals; 0—the initial value considered as the paw volume (**A**) or nociceptive reaction (**B**,**C**) before carrageenan administration. Statistical significance compared to vehicle-treated animals (control): * *p* < 0.05, ** *p* < 0.01, *** *p* < 0.001, **** *p* < 0.0001. Statistical analysis: two-way ANOVA post hoc Bonferroni test. Statistical significance in different time points of control group: ^#^ *p* < 0.05, ^##^ *p* < 0.01, ^###^ *p* < 0.001. Statistical analysis: one-way ANOVA, followed by Dunnett’s multiple comparison test (GraphPad Prism 8.0.1).

To test the anti-inflammatory activity and the potential to attenuate inflammatory pain, we tested compound **14** in the model of carrageenan-induced aseptic inflammation. In this model, the subplantar injection of carrageenan induced an edema, mechanical, and thermal hyperalgesia [31,32]. Figure 8A shows the time course of a rat paw edema development in the control group and the group pretreated with compound **14**. The tested compound was able to attenuate the formation of the paw edema significantly. The pretreatment with compound **14** at the dose of 80 mg/kg reduced the paw edema by 36.8%, 55.8%, 62.9%, 68.6%, and 74.3%, 1, 2, 3, 6-, and 24-h following carrageenan injection, respectively. Thus, the edema was the inflammation symptom, and the results suggested the anti-inflammatory activity of compound **14**, which may be considered as the additional effect to its analgesic activity resulting from its direct impact on nerves and processes of pain transduction and transmission. In our opinion, the most surprising and interesting was the long-lasting anti-edematous effect. It may result from the pharmacokinetic properties of compound **14**, but also from its unique mechanism. Since inflammation is one of the critical components of neuropathy, the anti-inflammatory activity of compound **14** may be a valuable and distinctive property from classical ASDs used to treat neuropathic pain [33]. Figure 8B shows that the subplantar injection of carrageenan decreased the withdrawal threshold (mechanical hyperalgesia). The pain threshold reached 87.3%, 90.9%, and 89.7% of its initial value 3, 6, and 24 h after carrageenan injection, respectively. The pretreatment with compound **14** at the dose of 80 mg/kg resulted in the inhibition of mechanical inflammatory hyperalgesia, observed as an increase in the withdrawal threshold to 113.3%, 110.1%, and 111.4% of the initial reaction. Figure 8C shows that the local injection of carrageenan-induced thermal hyperalgesia was observed as a decreased latency of a nociceptive response for radiant heat stimulation. The initial latency of the control group (before carrageenan injection) was 11.9 ± 0.9 s, and was significantly reduced to 62.4%, 65.1%, 45.6%, 45.8%, and 74.8% of the initial value 1, 2, 3, 6-, and 24-h following carrageenan injection, respectively. The initial latency for compound **14**-treated animals was 8.95 ± 0.4 s. The administration of compound **14** changed the values to 127.0%, 102.0%, 79.8%, 83.8%, and 164.4% of the initial value 1, 2, 3, 6-, and 24-h following carrageenan injection, respectively. Thus, compound **14** was able not only to attenuate the inflammatory hyperalgesia, but it also showed an analgesic effect for the heat stimulus. The main conclusion was that compound **14**, at a dose of 80 mg/kg, significantly reduced the nociceptive response in pain of different origins, including acute pain, persistent pain, neuropathic pain, and inflammatory hyperalgesia.

### 2.6. In Vitro Radioligand Binding Studies and Functional Assays

The previously described in vitro binding/functional results for chemical prototypes (see **I**, **II** in Figure 1) of compounds described in the present manuscript, revealed a potentially multi-targeted mechanism of action that included the antagonism of tetrodotoxin (TTX)-sensitive sodium (Na^+^) channels, Cav_1.2_ calcium channels, as well as the transient receptor potential cation channel vanilloid type 1 (TRPV1) [6,8]. Therefore, for two, the most effective anticonvulsants **14** and **(C1-*R*)-32** reported herein, we carried out binding assays to TTX-sensitive Na^+^ channels (that included Nav_1.1_, Nav_1.2_, Nav_1.3_, Nav_1.4_, Nav_1.6_, and Nav_1.7_ subtypes), calcium channel Cav_1.2_ (dihydropyridine site), as well as functional studies including the antagonist effect on Cav_1.2_ calcium channels and TRPV1 receptors (Table 3). It should be stresses that all the aforementioned channels/receptors were recognized as molecular targets for compounds with antiseizure or/and antinociceptive properties [34,35,36,37,38].

According to the results obtained, compounds **14** and **(C1-*R*)-32** revealed significant binding toward TTX-sensitive Na^+^ and Cav_1.2_ channels (dihydropyridine site) at a concentration of 50 μM. Furthermore, functional assays showed that compound **14** and **(C1-*R*)-32** possessed moderate Cav_1.2_ and weak TRPV1 antagonist activity at a concentration of 10 μM, whereas compound **14** revealed a relatively potent inhibition of the TRPV1 channel at 50 μM. The results obtained may probably indicate on the multimodal mechanism of action of compound **14** and **(C1-*R*)-32**, namely, the interaction with TTX-sensitive Na^+^ channels, voltage-gated Cav_1.2_ calcium channels, as well as TRPV1 receptors; nevertheless, further and more detailed binding/functional assays are necessary. It should be stressed, however, that on the basis of pharmacokinetic data obtained in mice *i.p.* (see below), and in a generally lower concentration of compound **14** in the brain (C_max_ = 10.9 μM, C_30 min._ = 7.2 μM) vs. serum (C_max_ = 41.1 μM, C_30 min._ = 24.3 μM) calculated for the free base, it may be concluded that anti-seizure activity was probably related with the inhibition of sodium and probably calcium conductance in the CNS. This assumption was confirmed especially by the patch-clamp studies, in which compound **14** significantly inhibited, and in a dose-depended manner, the fast voltage-gated sodium currents starting from the concentration of 0.1 μM (for details, see electrophysiological studies described below). Furthermore, the efficacy of compound **14** in the *sc*PTZ seizure test indicated its inhibitory influence on calcium currents mediated by Cav_1.2_ channels, as the sodium channels blockers (e.g., carbamazepine, LCS, lamotrigine, or phenytoin) were ineffective in this seizure model compared to multi-targeted ASDs characterized by interaction with, i.e., calcium channels, e.g., VPA [39]. Taking into consideration the antinociceptive properties of compound **14**, it seems that this activity may have both central (Nav_x_ channels) and peripheral (Nav_x_, Cav_1.2_, and TRPV1 channels) origin that may result from ca. four-fold higher concentration of compound in serum. It should be stressed that the most recent studies identify Cav_1.2_ channel as a promising molecular target for new analgesics [37,40]. The role of sodium channels (especially Nav_1.3_, Nav_1.6_, Nav_1.7_, Nav_1.8_, and Nav_1.9_ subtypes) and TRPV1 receptors in nociception and pain stimuli conductance remains unquestionable [36,41].

### 2.7. In Vitro Electrophysiological Studies

**Figure 9:**
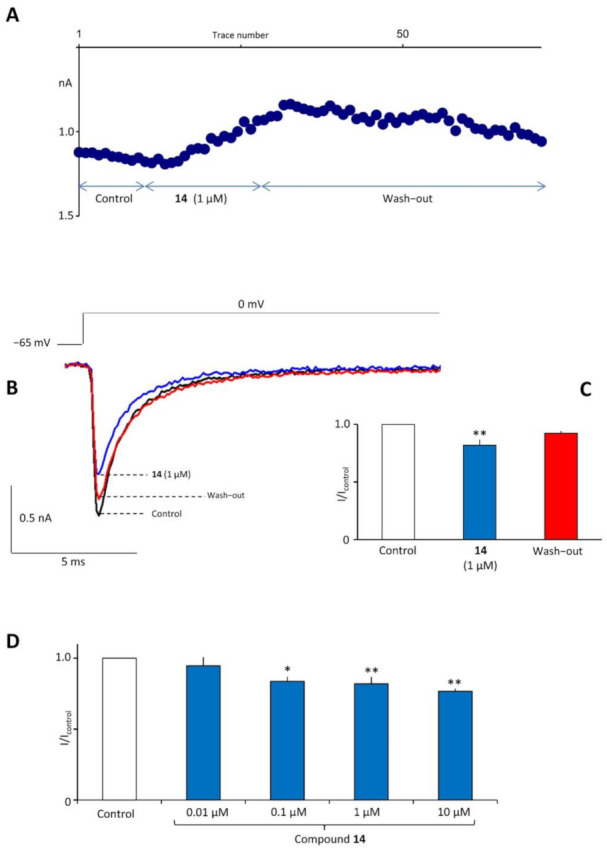
The influence of compound **14** on fast, voltage-gated sodium current. (**A**) The influence of tested compound on sodium current is shown on an example neuron. Current traces were evoked once every ten seconds by a rectangular voltage step. Vertical axis shows maximal current amplitudes (blue circles) in control, in the presence of compound **14** (1 µM) and after wash-out. Horizontal axis shows trace number. (**B**) Example sodium current recordings in control (black trace), after application of compound **14** (1 µM) (blue trace) and after wash-out (red trace). (**C**) Averaged normalized maximal current amplitudes in control, in the presence of compound **14** (1 µM) and after wash-out. (**D**) Averaged normalized maximal current amplitudes in control and in the presence of different concentrations of compound **14** (0.01 µM, 0.1 µM, 1 µM, and 10 µM). Statistical significance was analyzed by Graph Pad Prism 8.0.1 software using Tukey post hoc test (**C**) and Students’ *t*-test (**D**). Statistical significance-compared control: * *p* < 0.05, ** *p* < 0.01.

The activity of compound **14** in the electrically induced seizure models (e.g., MES and 6 Hz (32/44 mA)) and the results of binding studies, suggested its influence on neuronal sodium currents. Consequently, we determined the influence of compound **14** on the fast activating and fast inactivating voltage-gated sodium currents in rat prefrontal cortex pyramidal neurons using the patch-clamp technique. Maximal currents were evoked by rectangular voltage steps to 0 mV from a physiological holding potential of -65 mV. After recording a stable control, compound **14** was applied to the whole bath for three minutes. We found that compound **14** at a concentration of 1 µM inhibited the maximal amplitude of sodium currents (1.0 in control and 0.82 ± 0.05 after the application of compound **14**, Tukey post hoc test, *p* < 0.01, *n* = 5, Figure 9B). Sodium currents partially returned to control after drug wash-out (0.92 ± 0.02, Figure 9C). Notably, also a significant inhibition of sodium currents was observed for the parent compound **II** at a concentration of 1 μM (1.0 in control, 0.75 ± 0.04 in the presence of **II** (1 μM), Tukey post hoc test, *p* < 0.01, *n* = 5) reported previously [8]. Therefore, it was very likely that the potent inhibition of sodium currents by compound **14** similar to compound **II** was one of the mechanisms providing a similar level of anticonvulsant activity. Moreover, the influence of several additional concentrations of compound **14** on voltage-gated sodium currents were tested to confirm the dose–response relationship. In sum, the following concentrations were applied; 0.01 µM, 0.1 µM, 1 µM, 10 µM, and the results obtained were as follows: 0.95 ± 0.05 (*p* > 0.05, *n* = 5), 0.84 ± 0.03 (*p* < 0.05, *n* = 5), 0.82 ± 0.05 (*p* < 0.01, *n* = 5), and 0.77 ± 0.02 (*p* < 0.01, *n* = 5), respectively (Figure 9D, currents were normalized to control currents). It is worthy of note that the significant inhibitory effect was observed starting from the concentration of 0.1 µM, whereas no activity was detected at 0.01 µM. Furthermore, a clear dose–response relationship was visible. All the aforementioned results certainly supporting the inhibition of fast voltage-gated sodium currents, seemed to be crucial for the antiseizure activity of compound **14**.

### 2.8. Pharmacokinetic Studies

**Figure 10:**
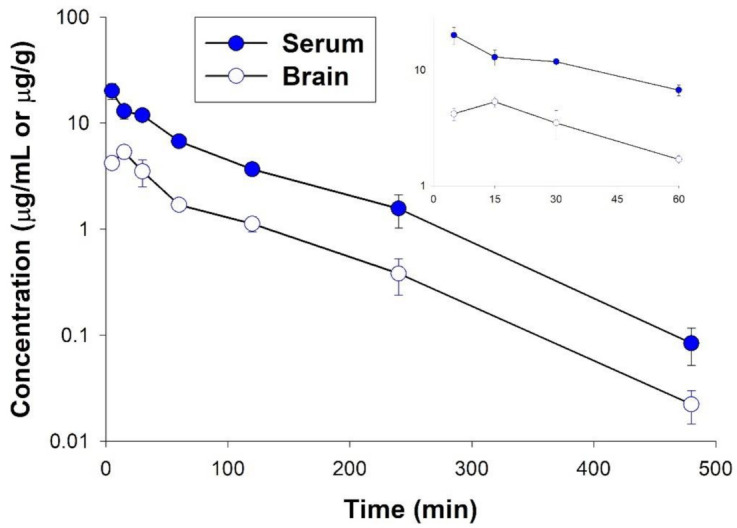
Concentrations of compound **14** measured in murine serum and brain tissue as a function of time following *i.p.* administration of this compound at a dose of 40 mg/kg. The inset shows the concentration–time curves close to the peak (0–60 min). The values represent the mean ± SD (*n* = 4).

We performed this study to investigate the pharmacokinetic profile of compound **14** after its *i.p*. administration in mice at a dose of 40 mg/kg, which corresponded to ED_50_s from the MES and 6 Hz (32 mA) seizure models. The amount of the target compound in the serum and brain was determined by an LC-MS/MS system. The concentrations versus time profiles of compound **14** are presented in Figure 10. It appears from this figure that the studied compound was very rapidly absorbed from the peritoneal cavity, as the peak concentration in the serum was achieved at the first observation time, i.e., 5 min. The compound also very quickly penetrated to the murine brain and reached the maximum concentration in this organ at 15 min. However, the brain concentrations were lower than those in the serum. The compound was slowly eliminated from the body as its concentrations were still measurable up to 8 h. The terminal slope reflecting the elimination rate from the brain was similar to that observed in the serum.

These observations were further confirmed by the values of pharmacokinetic parameters presented in Table 4. The elimination of half-lives estimated based on the terminal slope for the serum and brain tissue was almost identical. The volume of distribution was relatively high and significantly exceeded the volume of mouse blood or body water. This indicated that the compound was extensively distributed to tissues and organs. However, its brain penetration was not very high as expressed by a relatively low brain-to-serum AUC ratio that did not exceed 0.3. Compound **14** reached the highest concentration at the site of action 15 min after its administration (5.32 ± 0.54 µg/g tissue) and the brain levels slightly dropped to 3.50 ± 0.99 µg/g at the time of in vivo pharmacological testing, i.e., 30 min post-dose.

### 2.9. In Vitro ADME-Tox Assays

The in vitro determination of toxicity of new promising molecules with a therapeutic perspective is a crucial part of the assessment of the drug candidate quality. Drug–drug interactions (DDIs), as well as hepatotoxicity and neurotoxicity, are reported relatively frequently for various clinically used ASDs. Various CYP isoforms were studies, including the induction of CYP3A4, CYP2C9, CYP1A2, and CYP2B6 (phenytoin, phenobarbital, and carbamazepine) or the inhibition of CYP2C9 (VPA) [42,43,44,45]. Moreover, the idiosyncratic hepatotoxicity of certain ASDs may be due to the reactive metabolites produced by CYPs [46]. Thus, in the present study, we focused on the application of in vitro methods for the prediction of possible DDIs, hepatotoxicity, and neurotoxicity of the lead compound **14**. To this purpose, we examined the effect on the activity of CYP3A4 and 2D6 isoforms using commercial luminescent procedures, and on the viability of hepatoma HepG2 and neuroblastoma SH-SY5Y cell lines after a long 72 h treatment with several concentrations of compound **14**.

**Figure 11:**
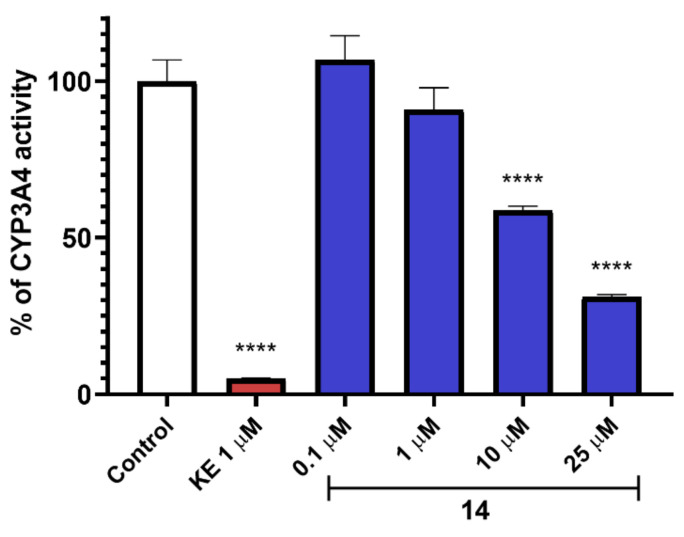
The influence of compound **14** and the reference inhibitor ketoconazole (KE) on CYP3A4 activity. Statistical significance (**** *p* < 0.0001) was analyzed by Graph Pad Prism 8.0.1 software using one-way ANOVA and Bonferroni’s multiple comparison post-test. The compound was examined in triplicate.

**Figure 12:**
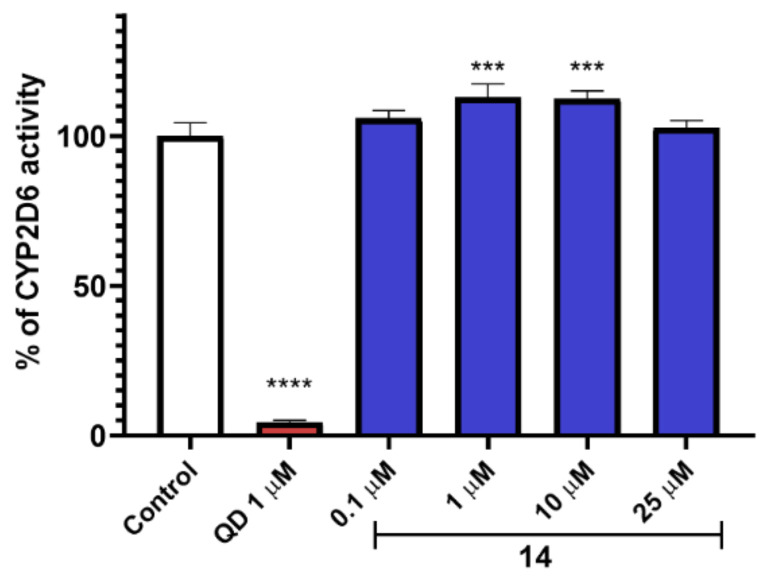
The influence of compound **14** and the reference inhibitor quinidine (QD) on CYP2D6 activity. Statistical significance (*** *p* < 0.001, **** *p* < 0.0001) was analyzed by Graph Pad Prism 8.0.1 software using one-way ANOVA and Bonferroni’s multiple comparison post-test. The compound was examined in triplicate.

The tested compound significantly (*p* < 0.0001) inhibited CYP3A4 activity, but only at the highest used doses of 10 µM and 25 µM. Moreover, its activity at 10 µM was higher than 50% (Figure 11) and indicated compound **14** as a moderate inhibitor of this isoform. The opposite, significant activation of CYP2D6 was observed at 1 and 10 µM concentrations of compound **14** (Figure 12). This effect was in accordance with the results obtained previously for other pyrrolidine-2,5-dione derivatives [6,12,47].

**Figure 13:**
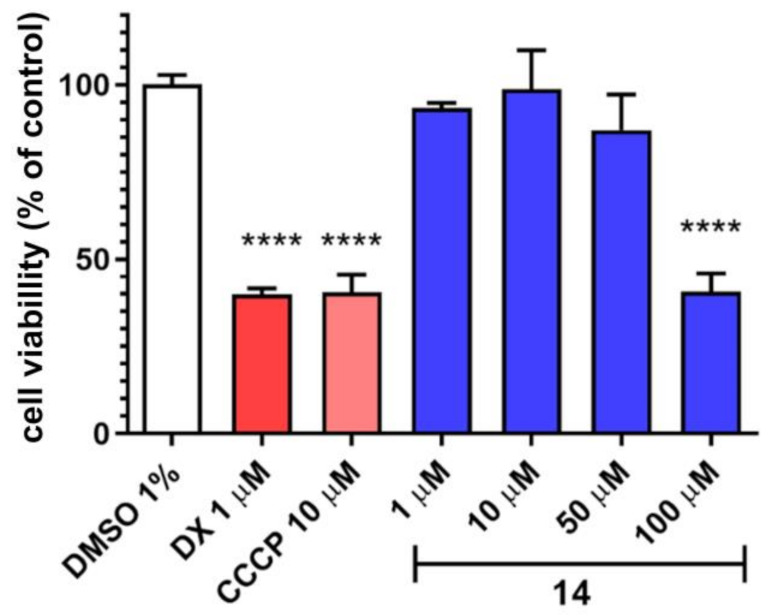
The influence of compound **14**, cytostatic drug doxorubicin (DX) and the mitochondrial toxin carbonyl cyanide 3-chlorophenyl-hydrazone (CCCP) on HepG2 cell line viability (72 h of incubation at 37 °C, 5% CO_2_). Statistical analysis (performed by GraphPad Prism 8.0.1); one-way ANOVA, followed by Bonferroni’s comparison test. The statistical significance: **** *p* < 0.0001 compared with negative control (DMSO 1% in growth media).

The hepatotoxicity testing of compound **14** with the use of the HepG2 cell line indicated that this compound was generally non-toxic. The effect on cell viability at 100 µM after 72 h treatment was similar to that observed for the reference cytostatic drug doxorubicin or the mitochondrial toxin carbonyl cyanide 3-chlorophenyl-hydrazone (CCCP), but at much lower concentrations of 1 and 10 µM, respectively (Figure 13).

**Figure 14:**
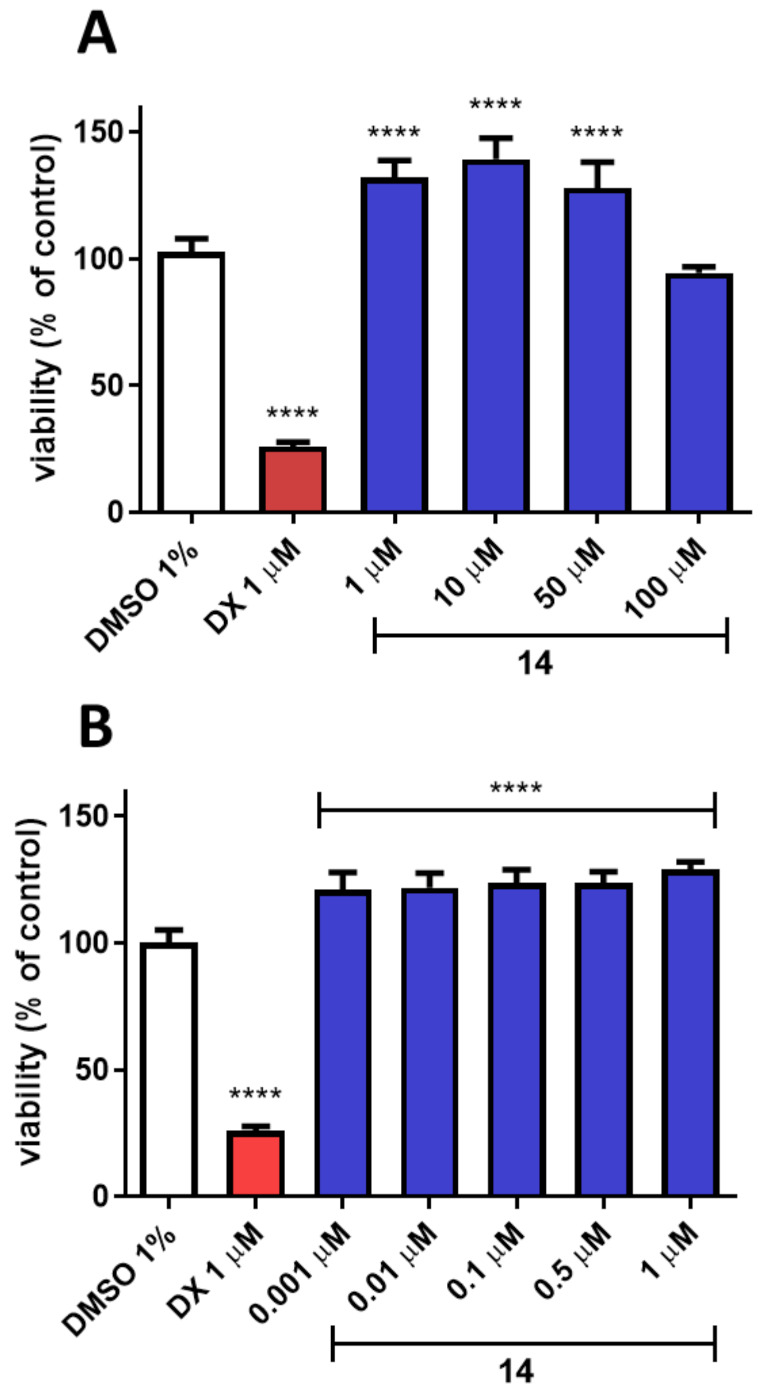
The effect of compound **14** and cytostatic drug doxorubicin (DX) on neuroblastoma SH-SY5Y cell line viability after 72 h of incubation at 37 °C, 5% CO_2_ in concentration ranges 1–100 µM (**A**) and 0.001–1 µM (**B**). The statistical significance (GraphPad Prism 8.0.1) was evaluated by a one-way ANOVA, followed by Bonferroni’s comparison test (**** *p* < 0.0001) compared with negative control (DMSO 1% in growth media).

Interestingly, the MTS test demonstrated that compound **14** significantly induced the viability of SH-SY5Y cells in almost all tested concentrations from 1 µM up to 50 µM (Figure 14A). Moreover, the repeated experiment showed a dose-dependent increase in cell viability in the concentration range from 0.001 to 1 µM (Figure 14B). The induction effect up to 120% of the control was observed even at the lowest used dose of 0.001 µM. The highest impact on SH-SY5Y cells was observed at 10 µM of compound **14** (139.7% of control viability). What is more, the similar beneficial effect on neurogenesis was determined previously for compound C11, the pyrrolidine-2,5-dione derivative, which effectively stimulated the viability of astrocytes up to 139.1% of the control [48].

## 3. Materials and Methods

### 3.1. Chemistry

All chemicals and solvents were purchased from commercial suppliers and were used without further purification. Melting points (mp.) were determined in open capillaries on a BÜCHI 353 melting point apparatus (BÜCHI Labortechnik, Flawil, Switzerland). TLC and the gradient UPLC chromatography were used to assess the purity and homogeneity of the compounds. TLC was carried out on silica gel 60 F_254_ pre-coated aluminum sheets (MACHEREY-NAGEL, Düren, Germany), using the following developing systems: S_1_–DCM:MeOH (9:0.2; *v*/*v*); S_2_–DCM:MeOH (9:0.3; *v*/*v*); S_3_–DCM:MeOH (9:0.5; *v*/*v*)). Spots detection: UV light (λ = 254 nm). The UPLC and mass spectra (LC-MS) were obtained on Waters ACQUITY™ TQD system (Waters, Milford, CT, USA) with the MS-TQ detector and UV–Vis-DAD eλ detector. The ACQUITY UPLC BEH C18, 1.7 μm (2.1 × 100 mm) column was used with the VanGuard Acquity UPLC BEH C18, 1.7 μm (2.1 × 5 mm) (Waters, Milford, CT, USA). Standard solutions (1 mg/mL) of each compound were prepared in analytical grade MeCN/water mixture (1:1; *v*/*v*). Conditions applied were as follows: eluent A (water/0.1% HCOOH), eluent B (MeCN/0.1% HCOOH), a flow rate of 0.3 mL/min, a gradient of 5–100% B over 10 min, and an injection volume of 10 μL. The UPLC retention times (*t*_R_) were given in minutes. Preparative column chromatography was performed using silica gel 60 (particle size 0.063–0.200; 70–230 Mesh ATM) purchased from Merck (Darmstadt, Germany). Elemental analyses (C, H, and N) for final compounds were carried out by a micro method using the elemental Vario EI III Elemental analyzer (Hanau, Germany). The results of elemental analyses were within ± 0.4% of the theoretical values. The ^1^H NMR and ^13^C NMR spectra were obtained in a JEOL-500 spectrometer (JEOL USA, Inc. Peabody, MA, USA), in CDCl_3_ operating at 500 MHz (^1^H NMR) and 126 MHz (^13^C NMR). Chemical shifts were reported in δ values (ppm) relative to TMS δ = 0 (^1^H), as internal standard. The *J* values were expressed in Hertz (Hz). Signal multiplicities were represented by the following abbreviations: s (singlet), d (doublet), dd (double doublet), ddd (double double doublet), dt (doublet of triplets), t (triplet), td (triplet of doublets), q (quartet), m (multiplet). Enantiomeric purity was determined using a chiral HPLC technique on a Shimadzu Prominence LC-2030C SD Plus system (Shimadzu Corporation, Kyoto, Japan) equipped with an Amylose-C (250 × 4.6 mm) chiral column. The analysis was performed under the following conditions: column temperature: 35 °C; mixture of eluents: hexane/i-PrOH = 95/5 (*v*/*v*); flow rate: 1 mL/min; injection volume: 10 μL; analysis time: 100 min. (isocratic); detection at the wavelength λ = 207 nm. Enantiomeric purity was expressed in %. For diastereomers, analyses were performed applying the following conditions: column temperature: 54 °C; mixture of eluents: hexane/i-PrOH = 95.2/4.8 (*v*/*v*); flow rate: 2.1 mL/min; injection volume: 10 μL; analysis time: 70 min. (isocratic); detection at the wavelength λ = 207 nm.

#### 3.1.1. Synthetic Procedure for Boc-Protected Compounds (**1**–**3** and (**C1-*R***)-**19**–(**C1-*R***)-**21**) and (**C1-*S***)-**19**

To the DCM (20 mL) solution of (*R*,*S*), (*R*) or (*S*)-2-((*tert*-butoxycarbonyl)amino)-2-phenylacetic acid (1.25 g, 5 mmol, 1 eq) was successively added DCC (1.55 g, 7.5 mmol, 1.5 eq) dissolved in 5 mL of DCM. After stirring (15 min), the appropriate phenylpiperazine (5 mmol, 1.0 eq) dissolved in 5 mL of DCM was added dropwise and the reaction was stirred at room temperature for 4 h. The DCM was evaporated in vacuo, and the product purified by column chromatography using a DCM:MeOH–9:0.3 (*v*/*v*) mixture as a solvent system.

*Tert*-butyl-(*R*,*S*)-(2-oxo-1-phenyl-2-(4-(3-(trifluoromethyl)phenyl)piperazin-1-yl)ethyl)carbamate (**1**).

Light oil. Yield: 85% (1.97 g); TLC: R_f_ = 0.54 (S_1_); UPLC (purity: >99%): *t*_R_ = 8.41 min. C_24_H_28_F_3_N_3_O_3_ (463.50). LC-MS (ESI): *m*/*z* calcd for C_24_H_28_F_3_N_3_O_3_ (M+H)^+^ 464.21, found 464.2.

*Tert*-butyl-(*R*,*S*)-(2-oxo-1-phenyl-2-(4-(3-(trifluoromethoxy)phenyl)piperazin-1-yl)ethyl)carbamate (**2**).

Light oil. Yield: 82% (1.86 g); TLC: R_f_ = 0.55 (S_1_); UPLC (purity: >99%): *t*_R_ = 8.85 min. C_24_H_28_F_3_N_3_O_4_ (479.50). LC-MS (ESI): *m*/*z* calcd for C_24_H_28_F_3_N_3_O_4_ (M+H)^+^ 480.20, found 480.1.

*Tert*-butyl-(*R*,*S*)-(2-oxo-1-phenyl-2-(4-(3-((trifluoromethyl)thio)phenyl)piperazin-1-yl)ethyl)carbamate (**3**). 

Light oil. Yield: 84% (1.89 g); TLC: R_f_ = 0.68 (S_2_); UPLC (purity: >99%): *t*_R_ = 8.67 min. C_24_H_28_F_3_N_3_O_3_S (495.56). LC-MS (ESI): *m*/*z* calcd for C_24_H_28_F_3_N_3_O_3_S (M+H)^+^ 496.18, found 496.2.

*Tert*-butyl-(*R*)-(2-oxo-1-phenyl-2-(4-(3-(trifluoromethyl)phenyl)piperazin-1-yl)ethyl) carbamate ((**C1-*R***)**-19**). 

Light oil. Yield: 87% (2.02 g); TLC: R_f_ = 0.62 (S_2_); UPLC (purity: >99%): *t*_R_ = 8.40 min. C_24_H_28_F_3_N_3_O_3_ (463.50). LC-MS (ESI): *m*/*z* calcd for C_24_H_28_F_3_N_3_O_3_ (M+H)^+^ 464.21, found 464.2.

*Tert*-butyl-(*S*)-(2-oxo-1-phenyl-2-(4-(3-(trifluoromethyl)phenyl)piperazin-1-yl)ethyl) carbamate ((**C1-*S***)-**19**). 

Light oil. Yield: 87% (2.12 g); TLC: R_f_ = 0.62 (S_2_); UPLC (purity: 100%): *t*_R_ = 8.40 min. C_24_H_28_F_3_N_3_O_3_ (463.50). LC-MS (ESI): *m*/*z* calcd for C_24_H_28_F_3_N_3_O_3_ (M+H)^+^ 464.21, found 464.1.

*Tert*-butyl-(*R*)-(2-oxo-1-phenyl-2-(4-(3-(trifluoromethoxy)phenyl)piperazin-1-yl)ethyl)carbamate ((**C1-*R***)-**20**). 

Light oil. Yield: 87% (2.07 g); TLC: R_f_ = 0.65 (S_2_); UPLC (purity: >99%): *t*_R_ = 8.51 min. C_24_H_28_F_3_N_3_O_4_ (479.50). LC-MS (ESI): *m/z* calcd for C_24_H_28_F_3_N_3_O_4_ (M+H)^+^ 480.20, found 480.2.

*Tert*-butyl-(*R*)-(2-oxo-1-phenyl-2-(4-(3-((trifluoromethyl)thio)phenyl)piperazin-1-yl)ethyl)carbamate ((**C1-*R***)-**21**).

Light oil. Yield: 79% (1.68 g); TLC: R_f_ = 0.70 (S_2_); UPLC (purity: >99%): *t*_R_ = 8.65 min. C_24_H_28_F_3_N_3_O_3_S (495.56). LC-MS (ESI): *m*/*z* calcd for C_24_H_28_F_3_N_3_O_3_S (M+H)^+^ 496.18, found 496.2.

#### 3.1.2. Synthetic Procedure for Amines **4**–**6**, (**C1-*R***)-**22**–(**C1-*R***)-**24**) and (**C1-*S***)-**22**

The DCM (5 mL) solution of **1**–**3**, (**C1-*R***)**-19**–(**C1-*R***)**-21 or (C1-*S***)**-19** (3 mmol, 1 eq) was treated with TFA (1.03 g, 0.67 mL, 9 mmol, 3 eq) and stirred at room temperature for 5 h. Next, DCM was evaporated in vacuo. The oil residue obtained was suspended in water (20 mL) and 25% ammonium hydroxide was carefully added to pH = 8. The aqueous phase was extracted using DCM (3 × 20 mL), dried over anhydrous Na_2_SO_4_, and concentrated to dryness. Compounds **4**–**6**, **(C1-*R*)-22**–**(C1-*R*)-24** and **(C1-*S*)-22** were obtained as an oil and used for further reactions without purification.

(*R*,*S*)-2-Amino-2-phenyl-1-(4-(3-(trifluoromethyl)phenyl)piperazin-1-yl)ethan-1-one (**4**). 

Light oil. Yield: 96% (1.12 g); UPLC (purity: >99%): *t*_R_ = 4.97 min. C_19_H_20_F_3_N_3_O (363.38). LC-MS (ESI): *m*/*z* calcd for C_19_H_20_F_3_N_3_O (M+H)^+^ 364.16, found 364.1.

(*R*,*S*)-2-Amino-2-phenyl-1-(4-(3-(trifluoromethoxy)phenyl)piperazin-1-yl)ethan-1-one (**5**). 

Light oil. Yield: 96% (1.15 g); UPLC (purity: >99%): *t*_R_ = 4.63 min. C_19_H_20_F_3_N_3_O_2_ (379.38). LC-MS (ESI): *m*/*z* calcd for C_19_H_20_F_3_N_3_O_2_ (M+H)^+^ 380.15, found 380.2.

(*R*,*S*)-2-Amino-2-phenyl-1-(4-(3-((trifluoromethyl)thio)phenyl)piperazin-1-yl)ethan-1-one (**6**). 

Light oil. Yield: 94% (1.03 g); UPLC (purity: >99%): *t*_R_ = 5.05 min. C_19_H_20_F_3_N_3_OS (395.44). LC-MS (ESI): *m*/*z* calcd for C_19_H_20_F_3_N_3_OS (M+H)^+^ 369.13, found 396.2.

(*R*)-2-Amino-2-phenyl-1-(4-(3-(trifluoromethyl)phenyl)piperazin-1-yl)ethan-1-one (**C1-*R***)-**22**. 

Light oil. Yield: 94% (1.09 g); UPLC (purity: >99%): *t*_R_ = 4.98 min. C_19_H_20_F_3_N_3_O (363.38). LC-MS (ESI): *m*/*z* calcd for C_19_H_20_F_3_N_3_O (M+H)^+^ 364.16, found 364.2.

(*S*)-2-Amino-2-phenyl-1-(4-(3-(trifluoromethyl)phenyl)piperazin-1-yl)ethan-1-one (**C1-*S***)-**22**. 

Light oil. Yield: 95% (1.10 g); UPLC (purity: >99%): *t*_R_ = 4.97 min. C_19_H_20_F_3_N_3_O (363.38). LC-MS (ESI): *m*/*z* calcd for C_19_H_20_F_3_N_3_O (M+H)^+^ 364.16, found 364.3.

(*R*)-2-Amino-2-phenyl-1-(4-(3-(trifluoromethoxy)phenyl)piperazin-1-yl)ethan-1-one (**C1-*R***)-**23**. 

Light oil. Yield: 95% (1.13 g); UPLC (purity: >99%): *t*_R_ = 4.63 min. C_19_H_20_F_3_N_3_O_2_ (379.38). LC-MS (ESI): *m*/*z* calcd for C_19_H_20_F_3_N_3_O_2_ (M+H)^+^ 380.15, found 380.2.

(*R*)-2-Amino-2-phenyl-1-(4-(3-((trifluoromethyl)thio)phenyl)piperazin-1-yl)ethan-1-one (**C1-*R***)-**24**. 

Light oil. Yield: 96% (1.16 g); UPLC (purity: >99%): *t*_R_ = 5.03 min. C_19_H_20_F_3_N_3_OS (395.44). LC-MS (ESI): *m*/*z* calcd for C_19_H_20_F_3_N_3_OS (M+H)^+^ 396.13, found 396.2.

#### 3.1.3. Synthetic Procedure for Maleamic Acids (**7**–**9**, (**C1-*R***)-**25**–(**C1-*R***)-**27** and (**C1-*S***)-**25**)

Maleic anhydride (0.98 g 10.0 mmol, 1 eq) was added to a solution of **4**–**6**, (**C1-*R***)**-22**–(**C1-*R***)**-24 or** (**C1-*S***)**-22** (10.0 mmol, 1 eq) in AcOEt (50 mL) and stirred for 30 min. After this time, the solvent was distilled off to dryness. The compound was obtained as solid after washing with diethyl ether (Et_2_O).

(*R*,*S*)-4-Oxo-4-((2-oxo-1-phenyl-2-(4-(3-(trifluoromethyl)phenyl)piperazin-1-yl)ethyl) amino)but-2-enoic acid (**7**). 

White solid. Yield: 82% (3.62 g); UPLC (purity: 98%): *t*_R_ = 6.90 min. LC-MS (ESI): *m*/*z* calcd for C_23_H_22_F_3_N_3_O_4_ (M+H) ^+^ 462.44, found 462.2.

(*R*,*S*)-4-Oxo-4-((2-oxo-1-phenyl-2-(4-(3-(trifluoromethoxy)phenyl)piperazin-1-yl)ethyl)amino)but-2-enoic acid (**8**). 

White solid. Yield: 84% (3.68 g); UPLC (purity: 99%): *t*_R_ = 7.04 min. LC-MS (ESI): *m*/*z* calcd for C_23_H_22_F_3_N_3_O_5_ (M+H) ^+^ 478.15, found 478.2.

(*R*,*S*)-4-Oxo-4-((2-oxo-1-phenyl-2-(4-(3-((trifluoromethyl)thio)phenyl)piperazin-1-yl)ethyl)amino)but-2-enoic acid (**9**). 

White solid. Yield: 86% (3.71 g); UPLC (purity: 98%): *t*_R_ = 7.12 min. LC-MS (ESI): *m*/*z* calcd for C_23_H_22_F_3_N_3_O_4_S (M+H) ^+^ 494.13, found 494.1.

(*R*)-4-Oxo-4-((2-oxo-1-phenyl-2-(4-(3-(trifluoromethyl)phenyl)piperazin-1-yl)ethyl) amino)but-2-enoic acid (**C1-*R***)-**25**. 

White solid. Yield: 85% (3.76 g); UPLC (purity: 96%): *t*_R_ = 6.92 min. LC-MS (ESI): *m*/*z* calcd for C_23_H_22_F_3_N_3_O_4_ (M+H) ^+^ 462.44, found 462.2.

(*S*)-4-Oxo-4-((2-oxo-1-phenyl-2-(4-(3-(trifluoromethyl)phenyl)piperazin-1-yl)ethyl) amino)but-2-enoic acid (**C1-*S***)-**25**. 

White solid. Yield: 87% (3.89 g); UPLC (purity: 98%): *t*_R_ = 6.92 min. LC-MS (ESI): *m*/*z* calcd for C_23_H_22_F_3_N_3_O_4_ (M+H) ^+^ 462.44, found 462.1.

(*R*)-4-Oxo-4-((2-oxo-1-phenyl-2-(4-(3-(trifluoromethoxy)phenyl)piperazin-1-yl)ethyl)amino)but-2-enoic acid (**C1-*R***)-**26**. 

White solid. Yield: 82% (3.43 g); UPLC (purity: 99%): *t*_R_ = 7.05 min. LC-MS (ESI): *m*/*z* calcd for C_23_H_22_F_3_N_3_O_5_ (M+H) ^+^ 478.15, found 478.2.

(*R*)-4-Oxo-4-((2-oxo-1-phenyl-2-(4-(3-((trifluoromethyl)thio)phenyl)piperazin-1-yl)ethyl)amino)but-2-enoic acid (**C1-*R***)-**27**. 

White solid. Yield: 87% (3.75 g); UPLC (purity: 97%): *t*_R_ = 7.10 min. LC-MS (ESI): *m*/*z* calcd for C_23_H_22_F_3_N_3_O_4_S (M+H) ^+^ 494.13, found 494.1.

#### 3.1.4. Synthetic Procedure for Unsaturated pyrrolidine-2,5-dione Derivative (**10**–**12**, (**C1-*R***)-**28**–(**C1-*R***)-**30**) and (**C1-*S***)-**28**

ZnCl_2_ (1.36 g, 10.0 mmol, 1 eq) was added to the suspension of **7**–**9**, (**C1-*R***)**-25**–(**C1-*R***)**-27 or** (**C1-*S***)**-25** (10.0 mmol, 1 eq) in dry benzene (100 mL), and the mixture was heated to 80 °C. Then, the solution of HMDS (15.0 mmol, 1.5 eq) in dry benzene (10 mL) was added dropwise over 30 minutes. The reaction was continued with stirring in reflux for about 24 h, then cooled and concentrated under reduced pressure. After distilling off the solvent, the oily residue was dissolved in DCM and extracted with 0.1 M HCl (3 × 50 mL), water (3 × 50 mL), and saturated NaCl solution (3 × 50 mL). The organic layer was dried over anhydrous Na_2_SO_4_ and then evaporated to dryness. The crude product was purified by column chromatography. The compound was obtained as solid after washing with Et_2_O.

(*R*,*S*)-1-(2-Oxo-1-phenyl-2-(4-(3-(trifluoromethyl)phenyl)piperazin-1-yl)ethyl)-1*H*-pyrrole-2,5-dione (**10**). 

White solid. Yield: 79% (3.34 g); UPLC (purity: >99%) *t*_R_ = 7.45 min. LC-MS (ESI): *m*/*z* calcd for C_23_H_20_F_3_N_3_O_3_ (M+H)^+^ 444.15 found 444.1.

(*R*,*S*)-1-(2-Oxo-1-phenyl-2-(4-(3-(trifluoromethoxy)phenyl)piperazin-1-yl)ethyl)-1*H*-pyrrole-2,5-dione (**11**). 

White solid. Yield: 75% (3.11 g); UPLC (purity: >99%) *t*_R_ = 7.51 min. LC-MS (ESI): *m*/*z* calcd for C_23_H_20_F_3_N_3_O_4_ (M+H)^+^ 460.14 found 460.2.

(*R*,*S*)-1-(2-Oxo-1-phenyl-2-(4-(3-((trifluoromethyl)thio)phenyl)piperazin-1-yl)ethyl)-1*H*-pyrrole-2,5-dione (**12**). 

White solid. Yield: 79% (3.34 g); UPLC (purity: >99%) *t*_R_ = 7.54 min. LC-MS (ESI): *m*/*z* calcd for C_23_H_20_F_3_N_3_O_4_S (M+H)^+^ 476.12 found 476.1.

(*R*)-1-(2-Oxo-1-phenyl-2-(4-(3-(trifluoromethyl)phenyl)piperazin-1-yl)ethyl)-1*H*-pyrrole-2,5-dione (**C1-*R***)-**28**. 

White solid. Yield: 74% (3.12 g); UPLC (purity: >99%) *t*_R_ = 7.44 min. LC-MS (ESI): *m*/*z* calcd for C_23_H_20_F_3_N_3_O_3_ (M+H)^+^ 444.15 found 444.1. Chiral HPLC > 99% ee (*t*_R_ = 76.57 min).

(*S*)-1-(2-Oxo-1-phenyl-2-(4-(3-(trifluoromethyl)phenyl)piperazin-1-yl)ethyl)-1*H*-pyrrole-2,5-dione (**C1-*S***)-**28**. 

White solid. Yield: 82% (3.42 g); UPLC (purity: >99%) *t*_R_ = 7.42 min. LC-MS (ESI): *m*/*z* calcd for C_23_H_20_F_3_N_3_O_3_ (M+H)^+^ 444.15 found 444.1. Chiral HPLC > 99% ee (*t*_R_ = 70.94 min).

(*R*)-1-(2-Oxo-1-phenyl-2-(4-(3-(trifluoromethoxy)phenyl)piperazin-1-yl)ethyl)-1*H*-pyrrole-2,5-dione (**C1-*R***)-**29**. 

White solid. Yield: 78% (3.16 g); UPLC (purity: >99%) *t*_R_ = 7.52 min. LC-MS (ESI): *m*/*z* calcd for C_23_H_20_F_3_N_3_O_4_ (M+H)^+^ 460.14 found 460.2. Chiral HPLC > 99% ee (*t*_R_ = 74.20 min).

(*R*)-1-(2-Oxo-1-phenyl-2-(4-(3-((trifluoromethyl)thio)phenyl)piperazin-1-yl)ethyl)-1*H*-pyrrole-2,5-dione (**C1-*R***)-**30**. 

White solid. Yield: 81% (3.45 g); UPLC (purity: >99%) *t*_R_ = 7.54 min. LC-MS (ESI): *m*/*z* calcd for C_23_H_20_F_3_N_3_O_4_S (M+H)^+^ 476.12 found 476.1. Chiral HPLC > 99% ee (*t*_R_ = 78.82 min).

#### 3.1.5. Synthetic Procedure for Target Hydrochlorides (**13**–**18**, (**C1-*R***)-**31**–(**C1-*R***)-**33**) and (**C1-*S***)-**31**

The respective primary or secondary amine solution in THF (2.2 mmol, 1 eq) was added to a solution of **10**–**12**, (**C1-*R***)**-28**–(**C1-*R***)**-30**, or (**C1-*S***)**-28** (2.2 mmol, 1 eq) in dry benzene (50 mL). The reaction was continued with stirring for about 8 h, then concentrated under reduced pressure. The crude product was purified by column chromatography. The compound was then converted into the hydrochloride salt by treating the compound with a 2M methanolic hydrochloric acid solution. The compound was obtained as solid after washing with Et_2_O.

3-(Methylamino)-1-(2-oxo-1-phenyl-2-(4-(3-(trifluoromethyl)phenyl)piperazin-1-yl)ethyl)pyrrolidine-2,5-dione hydrochloride (**13**). 

White solid. Yield: 87% (0.91 g); mp. 161.2–163.4 °C; TLC: R_f_ = 0.42 (S_3_); UPLC (purity: >99%): *t*_R_ = 5.49 min. LC-MS (ESI): *m*/*z* calcd for C_24_H_25_F_3_N_4_O_3_ (M+H)^+^ 475.48, found 475.3; ^1^H NMR (500 MHz, CDCl_3_) δ 2.47 (s, 3H), 2.55 (dd, *J* = 18.0, 5.2 Hz, 1H), 2.69–2.77 (m, 1H), 2.95 (dd, *J* = 18.3, 8.6 Hz, 1H), 3.04–3.17 (m, 2H), 3.23–3.39 (m, 4H), 3.68 (dd, *J* = 8.7, 5.0 Hz, 2H), 4.00 (ddd, *J* = 9.9, 6.3, 3.0 Hz, 1H), 6.10 (s, 1H), 6.94–6.98 (m, 1H), 7.01 (s, 1H), 7.09 (d, *J* = 7.7 Hz, 1H), 7.30–7.38 (m, 4H), 7.40–7.44 (m, 2H); ^13^C NMR (126 MHz, CDCl_3_) δ 29.8, 33.5, 42.4, 45.7, 48.5, 48.8, 56.8, 57.3, 112.8, 116.9, 119.3, 124.2 (q, *J* = 272.8 Hz), 128.9, 129.2, 129.8, 129.9, 131.6 (q, *J* = 31.8 Hz), 132.6, 150.9, 165.0, 174.4, 177.3. Anal. calcd for C_24_H_26_ClF_3_N_4_O_3_ (510.94): C: 56.42, H: 5.13, N: 10.97; Found C: 56.30, H: 5.27, N: 10.81.

3-(Dimethylamino)-1-(2-oxo-1-phenyl-2-(4-(3-(trifluoromethyl)phenyl)-piperazin-1-yl)ethyl)pyrrolidine-2,5-dione hydrochloride (**14**). 

White solid. Yield: 83% (0.90 g); mp. 157.8–159.2 °C; TLC: R_f_ = 0.49 (S_3_); UPLC (purity: >99%): *t*_R_ = 5.59 min. LC-MS (ESI): *m*/*z* calcd for C_25_H_27_F_3_N_4_O_3_ (M+H)^+^ 489.20, found 489.3; ^1^H NMR (500 MHz, CDCl_3_) δ 2.41 (s, 6H), 2.61 (dd, *J* = 18.6, 4.9 Hz, 1H), 2.68–2.74 (m, 1H), 2.84 (dd, *J* = 18.6, 9.12 Hz, 1H), 3.04–3.08 (m, 1H), 3.12 (ddd, *J* = 11.8, 8.3, 3.2 Hz, 1H), 3.24–3.36 (m, 3H), 3.66–3.71 (m, 1H), 3.74 (dd, *J* = 9.2, 4.9 Hz, 1H), 3.97–4.04 (m, 1H), 6.13 (s, 1H), 6.96 (dd, *J* = 8.3, 2.0 Hz, 1H), 7.01 (s, 1H), 7.08 (d, *J* = 7.5 Hz, 1H), 7.29–7.38 (m, 4H), 7.40–7.43 (m, 2H); ^13^C NMR (126 MHz, CDCl_3_) δ 31.1, 41.4, 42.4, 45.7, 48.5, 48.7, 56.7, 62.3, 112.8, 116.8, 119.3, 124.2 (q, *J* = 272.8 Hz), 128.9, 129.1, 129.8, 131.6 (q, *J* = 32.0 Hz), 132.8, 150.9, 165.0, 174.5, 175.7. Anal. calcd for C_25_H_28_ClF_3_N_4_O_3_ (524.97): C: 57.20, H: 5.38, N: 10.67; Found C: 57.30, H: 5.29, N: 10.84.

3-(Diethylamino)-1-(2-oxo-1-phenyl-2-(4-(3-(trifluoromethyl)phenyl)piperazin-1-yl)ethyl)pyrrolidine-2,5-dione hydrochloride (**15**). 

White solid. Yield: 88% (1.00 g); mp. 142.2–143.1 °C; TLC: R_f_ = 0.52 (S_3_); UPLC (purity: >99%): *t*_R_ = 5.79 min. LC-MS (ESI): *m*/*z* calcd for C_27_H_31_F_3_N_4_O_3_ (M+H)^+^ 517.23, found 517.3; ^1^H NMR (500 MHz, CDCl_3_) δ 1.07 (t, *J* = 7.2 Hz, 6H), 2.55–2.63 (m, 4H), 2.69–2.76 (m, 2H), 2.86 (dd, *J* = 18.3, 9.2 Hz, 1H), 3.06 (dt, *J* = 12.0, 2.9 Hz, 1H), 3.10–3.15 (m, 1H), 3.26–3.35 (m, 3H), 3.67–3.71 (m, 1H), 3.98 (dd, *J* = 9.2, 5.4 Hz, 1H), 4.00–4.07 (m, 1H), 6.13 (s, 1H), 6.96 (dd, *J* = 8.3, 2.0 Hz, 1H), 7.01 (s, 1H), 7.09 (d, *J* = 7.7 Hz, 1H), 7.30–7.37 (m, 4H), 7.40–7.43 (m, 2H).^13^C NMR (126 MHz, CDCl_3_) δ 13.8, 32.2, 42.4, 44.9, 45.7, 48.5, 48.8, 56.7, 58.8, 112.8, 116.8, 119.3, 124.2 (d, *J* = 272.8 Hz), 128.8, 129.1, 129.8, 131.6, 132.8, 150.9, 165.1, 174.7, 176.7. Anal. calcd for C_27_H_32_ClF_3_N_4_O_3_ (553.02): C: 58.64, H: 5.83, N: 10.13; Found C: 58.78, H: 5.69, N: 10.38.

3-Morpholino-1-(2-oxo-1-phenyl-2-(4-(3-(trifluoromethyl)phenyl)piperazin-1-yl)ethyl)pyrrolidine-2,5-dione hydrochloride (**16**). 

White solid. Yield: 84% (0.99 g); mp. 146.2–147.5 °C; TLC: R_f_ = 0.58 (S_3_); UPLC (purity: >99%): *t*_R_ = 6.75 min. LC-MS (ESI): *m*/*z* calcd for C_27_H_29_F_3_N_4_O_4_ (M+H)^+^ 531.21, found 531.4; ^1^H NMR (500 MHz, CDCl_3_) δ 2.61–2.67 (m, 3H), 2.68–2.73 (m, 1H), 2.79 (s, 2H), 2.87 (dd, *J* = 18.6, 9.2 Hz, 1H), 3.04–3.08 (m, 1H), 3.13 (ddd, *J* = 11.9, 8.5, 3.2 Hz, 1H), 3.24–3.37 (m, 3H), 3.65–3.73 (m, 6H), 4.01 (ddd, *J* = 13.3, 5.9, 3.4 Hz, 1H), 6.13 (s, 1H), 6.96 (dd, *J* = 8.5, 2.4 Hz, 1H), 7.01 (s, 1H), 7.09 (d, *J* = 7.7 Hz, 1H), 7.30–7.38 (m, 4H), 7.40–7.43 (m, 2H); ^13^C NMR (126 MHz, CDCl_3_) δ 31.0, 45.7, 48.5, 48.8, 49.4, 56.8, 62.3, 67.0, 112.9, 116.9, 119.3, 124.2 (q, *J* = 272.2 Hz), 128.9, 129.2, 129.8, 129.9, 131.6 (q, *J* = 32.0 Hz), 132.6, 150.9, 165.0, 174.3, 175.1. Anal. calcd for C_27_H_30_ClF_3_N_4_O_4_ (567.01): C: 57.19, H: 5.33, N: 9.88; Found C: 57.31, H: 5.51, N: 10.08.

3-Dimethylamino-1-(2-oxo-1-phenyl-2-(4-(3-(trifluoromethoxy)phenyl)piperazin-1-yl)ethyl)pyrrolidine-2,5-dione hydrochloride (**17**). 

White solid. Yield: 82% (0.96 g); mp. 152.3–153.5 °C; TLC: R_f_ = 0.62 (S_3_); UPLC (purity: >99%): *t*_R_ = 5.90 min. LC-MS (ESI): *m*/*z* calcd for C_25_H_27_F_3_N_4_O_4_ (M+H)^+^ 505.20, found 505.3 ^1^H NMR (500 MHz, CDCl_3_) δ 2.43 (s, 6H), 2.63 (dd, *J* = 18.5, 5.0 Hz, 1H), 2.67–2.71 (m, 1H), 2.85 (dd, *J* = 18.6, 9.2 Hz, 1H), 3.00–3.06 (m, 1H), 3.07–3.14 (m, 1H), 3.24–3.34 (m, 3H), 3.68 (ddd, *J* = 13.2, 8.0, 3.2 Hz, 1H), 3.76 (dd, *J* = 9.2, 4.9 Hz, 1H), 3.99 (td, *J* = 6.5, 3.6 Hz, 1H), 6.13 (s, 1H), 6.61 (s, 1H), 6.69–6.73 (m, 2H), 7.21 (t, *J* = 8.2 Hz, 1H), 7.33–7.38 (m, 3H), 7.40–7.42 (m, 2H); ^13^C NMR (126 MHz, CDCl_3_) δ 31.1, 41.4, 42.3, 45.7, 48.4, 48.7, 56.7, 62.3, 108.9, 112.2, 114.3, 120.4 (q, *J* = 257.1 Hz), 128.9, 129.1, 129.8, 130.3, 132.8, 150.3, 152.0, 165.0, 174.4, 175.6. Anal. calcd for C_25_H_28_ClF_3_N_4_O_4_ (540.97): C: 55.51, H: 5.22, N: 10.36; Found C: 55.78, H: 5.14, N: 10.49.

3-Dimethylamino-1-(2-oxo-1-phenyl-2-(4-(3-((trifluoromethyl)thio)phenyl)piperazin-1-yl)ethyl)pyrrolidine-2,5-dione hydrochloride (**18**). 

White solid. Yield: 81% (0.97 g); mp. 170.1–171.5 °C; TLC: R_f_ = 0.65 (S_3_); UPLC (purity: >99%): *t*_R_ = 6.08 min. LC-MS (ESI): *m*/*z* calcd for C_25_H_27_F_3_N_4_O_3_S (M+H)^+^ 521.18, found 521.1; ^1^H NMR (500 MHz, CDCl_3_) δ 2.42 (s, 6H), 2.61 (dd, *J* = 18.6, 4.9 Hz, 1H), 2.67–2.72 (m, 1H), 2.84 (dd, *J* = 18.6, 9.2 Hz, 1H), 3.01–3.07 (m, 1H), 3.09–3.15 (m, 1H), 3.25–3.35 (m, 3H), 3.65–3.72 (m, 1H), 3.74 (dd, *J* = 9.2, 4.9 Hz, 1H), 3.97–4.03 (m, 1H), 6.13 (s, 1H), 6.91 (ddd, *J* = 8.5, 2.4, 0.9 Hz, 1H), 7.05 (s, 1H), 7.12 (d, *J* = 7.5 Hz, 1H), 7.24–7.28 (m, 1H), 7.33–7.38 (m, 3H), 7.40–7.42 (m, 2H); ^13^C NMR (126 MHz, CDCl_3_) δ 31.1, 41.4, 42.4, 45.7, 48.4, 48.7, 56.7, 62.3, 118.5, 123.7, 127.8, 128.8 (q, *J* = 308.4 Hz), 128.9, 129.1, 129.8, 130.1, 132.8, 151.3, 165.0, 174.5, 175.7. Anal. calcd for C_25_H_28_ClF_3_N_4_O_3_S (557.03): C: 53.91, H: 5.07, N: 10.06 Found: C: 53.82, H: 5.14, N: 10.32.

3-Dimethylamino-1-((*R*)-oxo-1-phenyl-2-(4-(3-(trifluoromethyl)phenyl)-piperazin-1-yl)ethyl)pyrrolidine-2,5-dione hydrochloride (**C1-*R***)-**31**. 

White solid. Yield: 85% (0.92 g); mp. 169.8–170.6 °C; TLC: R_f_ = 0.49 (S_3_); UPLC (purity: >99%): *t*_R_ = 5.73 min. LC-MS (ESI): *m*/*z* calcd for C_25_H_27_F_3_N_4_O_3_ (M+H)^+^ 489.20, found 489.4; ^1^H NMR (500 MHz, CDCl_3_) δ 2.42 (s, 6H), 2.61 (dd, *J* = 18.6, 4.9 Hz, 1H), 2.67–2.74 (m, 1H), 2.84 (dd, *J* = 18.6, 9.2 Hz, 1H), 3.03–3.09 (m, 1H), 3.13 (ddd, *J* = 11.8, 8.2, 3.2 Hz, 1H), 3.24–3.36 (m, 3H), 3.65–3.72 (m, 1H), 3.74 (dd, *J* = 9.2, 4.9 Hz, 1H), 3.97–4.04 (m, 1H), 6.13 (s, 1H), 6.96 (dd, *J* = 8.3, 2.3 Hz, 1H), 7.01 (s, 1H), 7.09 (d, *J* = 7.5 Hz, 1H), 7.30–7.37 (m, 4H), 7.40–7.43 (m, 2H); ^13^C NMR (126 MHz, CDCl_3_) δ 31.1, 41.4, 42.4, 45.7, 48.5, 48.8, 56.7, 62.3, 112.8, 116.8, 119.3, 124.2 (q, *J* = 272.6 Hz), 128.9, 129.1, 129.8, 131.6 (q, *J* = 32.0 Hz), 132.8, 150.9, 165.0, 174.5, 175.8. Anal. calcd for C_25_H_28_ClF_3_N_4_O_3_ (524.97): C: 57.20, H: 5.38, N: 10.67; Found: C: 57.27, H: 5.42, N: 10.59.

3-Dimethylamino-1-((*S*)-oxo-1-phenyl-2-(4-(3-(trifluoromethyl)phenyl)-piperazin-1-yl)ethyl)pyrrolidine-2,5-dione hydrochloride (**C1-*S***)-**31**. 

White solid. Yield: 88% (0.95 g); mp. 169.2–170.8 °C; TLC: R_f_ = 0.49 (S_3_); UPLC (purity: >99%): *t*_R_ = 5.53 min. LC-MS (ESI): *m/z* calcd for C_25_H_27_F_3_N_4_O_3_ (M+H)^+^ 489.20, found 489.2; ^1^H NMR (500 MHz, CDCl_3_) δ 2.42 (s, 6H), 2.61 (dd, *J* = 18.6, 4.9 Hz, 1H), 2.67–2.74 (m, 1H), 2.84 (dd, *J* = 18.6, 9.2 Hz, 1H), 3.03–3.09 (m, 1H), 3.13 (ddd, *J* = 11.8, 8.2, 3.2 Hz, 1H), 3.24–3.37 (m, 3H), 3.65–3.72 (m, 1H), 3.74 (dd, *J* = 9.2, 4.9 Hz, 1H), 3.97–4.04 (m, 1H), 6.13 (s, 1H), 6.96 (dd, *J* = 8.3, 2.3 Hz, 1H), 7.01 (s, 1H), 7.09 (d, *J* = 7.5 Hz, 1H), 7.30–7.38 (m, 4H), 7.40–7.43 (m, 2H); ^13^C NMR (126 MHz, CDCl_3_) δ 31.1, 41.4, 42.4, 45.7, 48.5, 48.8, 56.7, 62.3, 112.8, 116.8, 119.3, 124.2 (q, *J* = 272.6 Hz), 128.9, 129.1, 129.8, 131.6 (q, *J* = 32.0 Hz), 132.8, 150.9, 165.0, 174.5, 175.8. Anal. calcd for C_25_H_28_ClF_3_N_4_O_3_ (524.97): C: 57.20, H: 5.38, N: 10.67; Found: C: 57.31, H: 5.27, N: 10.72.

3-Dimethylamino-1-((*R*)-2-oxo-1-phenyl-2-(4-(3-(trifluoromethoxy)phenyl)piperazin-1-yl)ethyl)pyrrolidine-2,5-dione hydrochloride (**C1-*R***)-**32**. 

White solid. Yield: 79% (0.92 g); mp. 164.8–165.7 °C; TLC: R_f_ = 0.62 (S_3_); UPLC (purity: >99%): *t*_R_ = 5.91 min. LC-MS (ESI): *m*/*z* calcd for C_25_H_27_F_3_N_4_O_4_ (M+H)^+^ 505.20, found 505.3; ^1^H NMR (500 MHz, CDCl_3_) δ 2.42 (s, 6H), 2.62 (dd, *J* = 18.6, 4.9 Hz, 1H), 2.67–2.72 (m, 1H), 2.85 (dd, *J* = 18.6, 9.2 Hz, 1H), 3.01–3.05 (m, 1H), 3.09–3.13 (m, 1H), 3.24–3.34 (m, 3H), 3.65–3.70 (m, 1H), 3.75 (dd, *J* = 9.2, 4.9 Hz, 1H), 3.96–4.02 (m, 1H), 6.13 (s, 1H), 6.61 (s, 1H), 6.68–6.73 (m, 2H), 7.21 (t, *J* = 8.3 Hz, 1H), 7.33–7.38 (m, 3H), 7.40–7.42 (m, 2H); ^13^C NMR (126 MHz, CDCl_3_) δ 31.1, 41.4, 42.3, 45.6, 48.3, 48.7, 56.7, 62.3, 108.9, 112.2, 114.3, 120.5 (q, *J* = 257.1 Hz), 128.9, 129.1, 129.8, 130.3, 132.8, 150.3, 152.0, 165.0, 174.5, 175.7. Anal. calcd for C_25_H_28_ClF_3_N_4_O_4_ (540.97): C: 55.51, H: 5.22, N: 10.36; Found: C: 55.62, H: 5.18, N: 10.28.

3-Dimethylamino-1-((*R*)-2-oxo-1-phenyl-2-(4-(3-((trifluoromethyl)thio)phenyl)piperazin-1-yl)ethyl)pyrrolidine-2,5-dione hydrochloride (**C1-*R***)-**33**. 

White solid. Yield: 79% (0.94 g); mp. 173.2–174.7 °C; TLC: R_f_ = 0.65 (S_3_); UPLC (purity: >99%): *t*_R_ = 6.08 min. LC-MS (ESI): *m*/*z* calcd for C_25_H_27_F_3_N_4_O_3_S (M+H)^+^ 521.18, found 521.1; ^1^H NMR (500 MHz, CDCl_3_) δ 2.42 (s, 6H), 2.61 (dd, *J* = 18.6, 4.9 Hz, 1H), 2.68–2.73 (m, 1H), 2.84 (dd, *J* = 18.5, 9.3 Hz, 1H), 3.01–3.07 (m, 1H), 3.11 (ddd, *J* = 11.7, 8.3, 3.0 Hz, 1H), 3.24–3.36 (m, 3H), 3.66–3.71 (m, 1H), 3.75 (dd, *J* = 9.2, 4.9 Hz, 1H), 3.96–4.03 (m, 1H), 6.13 (s, 1H), 6.89–6.93 (m, 1H), 7.05 (s, 1H), 7.12 (d, *J* = 7.5 Hz, 1H), 7.24–7.27 (m, 1H), 7.31–7.38 (m, 3H), 7.40–7.42 (m, 2H); ^13^C NMR (126 MHz, CDCl_3_) δ 31.1, 41.4, 42.4, 45.7, 48.4, 48.7, 56.7, 62.3, 118.5, 123.7, 127.8, 128.9 (q, *J* = 308.4 Hz), 129.1, 129.8, 130.1, 132.8, 151.4, 165.0, 174.5, 175.8. Anal. calcd for C_25_H_28_ClF_3_N_4_O_3_S (557.03): C: 53.91, H: 5.07, N: 10.06; Found: C: 53.82, H: 5.14, N: 10.32.

### 3.2. Anticonvulsant Activity and Acute Neurotoxicity

In this study, we used adult male CD-1 mice (accredited animal facility Jagiellonian University Medical College, Krakow, Poland) that weighed between 22 and 26 g. They were housed under standardized housing conditions in colony cages and had free access to food, as well as tap water. Four mice per group were randomly assigned to each experimental group (the initial anticonvulsant screening) with each mouse being used only once. To evaluate the ED_50_ or TD_50_ values, 3–4 groups of six animals were injected with various doses of tested compounds. All procedures involving animals and their care were performed in accordance with the current European Union Directive of 22 September 2010 (2010/63/EU) and Polish legislation on animal experimentation. The studies were carried out under experimental protocols approved by the I Local Ethical Committee for Experiments on Animals of the Jagiellonian University in Krakow, Poland (nos. 165/2018, 228A/2019, and 360/2019). All substances were suspended in Tween 80 (1% aqueous solution) and administered *i.p.* as a single injection at a dose of 10 mL/kg body weight. On each day of experimentation, fresh solutions were prepared. 

The detailed in vivo procedures were described elsewhere; maximal electroshock seizure test (MES) [14], subcutaneous pentylenetetrazole seizure test (*sc*PTZ) [14], the 6 Hz (32 and 44 mA) psychomotor seizure model [49], the rotarod test for acute neurological toxicity [50].

#### Data Analysis—Anticonvulsant Activity and Neurotoxicity Studies

The ED_50_ and TD_50_ values with 95% confidence limits were calculated by probit analysis [20]. The protective indexes for the compounds investigated and reference ASDs were calculated by dividing the TD_50_ value, as determined in the rotarod test, by the respective ED_50_ value, as determined in the MES, *sc*PTZ, or 6 Hz (32 mA or 44 mA) tests. The protective index was considered as an index of the margin of safety and tolerability between anticonvulsant doses and doses of the compounds exerting acute adverse effects such as sedation, motor coordination impairment, ataxia, or other neurotoxic manifestations.

### 3.3. Intravenous (iv) Pentylenetetrazole (PTZ) Seizure Threshold Test, Rectal Temperature Measurement and Grip Strength Test

In this study, we used adult male Swiss albino mice (Kołacz, Laboratory Animals Breeding, Warszawa, Poland) license no. 106/2019 (Local Ethical Committee in Lublin). In studies assessing the acute effect of compound **14** on neuromuscular strength, body temperature, and seizure threshold, compound **14** was suspended in a 1% solution of Tween 80 and administered *i.p.*, 30 min before the tests. 

The timed *iv*PTZ test was employed to evaluate the acute effect of compound **14** on the seizure thresholds for (1) the first myoclonic twitch, (2) generalized clonic seizure with loss of righting reflex, and (3) forelimb tonus. The experimental procedure was described in detail elsewhere [26]. The changes in temperature were recorded using an electronic thermometer (ThermoWorks, Alpine, UT, USA). Mice were gently restrained and a rectal probe was inserted to a depth of ~2 cm into the rectum. 

The probe remained in the rectum for several second, allowing for temperature stabilization. The temperature was recorded just before injection of compound **14** or vehicle and 30 min later. The difference between the pre- and post-injection temperature value was then calculated (Δ °C). 

The acute effect of compound **14** on neuromuscular strength was quantified using the grip-strength apparatus (BIOSEB, Chaville, France) according to the method described elsewhere [26].

### 3.4. Antinociceptive Activity

The experiments were conducted on adult male Albino Swiss mice (CD-1, 18–25 g) and male Wistar rats (Krf:(WI) WU), 180–250 g) as described previously [51]. The minimum number of animals used needed to obtain definite and normally distributed results with the utilized test. Behavioral measures were scored by trained observers, which were blind to experimental conditions. The following devices were used in the experiments: Analgesy Meter (37215, Ugo Basile), Plantar Test Apparatus (EFV5, Commat Ltd., Turkey), Plethysmometer (Plethysmometer 7140, Ugo Basile), Electronic von Frey unit (BIOSEB, France). All procedures were performed according to the European Union Directive of 22 September 2010 (2010/63/EU) and approved by the Local Ethics Committee for Experiments on Animals in Krakow, Poland (resolution no. 105/2016).

#### Data Analysis—Antinociceptive Activity Studies

Data were presented as means ± standard error of the mean (S.E.M). The Graph Pad Prism 8.0.1 was used to analyze the vast majority of data. Statistically significant differences between groups were calculated using one-way analysis of variance (ANOVA) and the post hoc Dunnett’s multiple comparison test or two-way analysis of variance (ANOVA) and the post hoc Tukey’s comparison when appropriate. The criterion for significance was set at *p* < 0.05. The log-probit method was applied to statistically determine the ED_50_ values with 95% confidence limits

### 3.5. Pharmacokinetic Study

Male CD-1 mice weighting 28–33 g housed in conditions of the constant temperature with a 12:12 h light–dark cycle with free access to food and water were used in this study. The investigated compound was dissolved in sterile water for injection (Polpharma, Poland) and administered *i.p*. at a dose of 40 mg/kg. The mice were sacrificed by decapitation under isoflurane anesthesia. Blood samples were collected at 5, 15, 30, 60, 120, 240, and 480 min after dosing and brains were harvested at the same time points. Blood was allowed to clot at room temperature for 20 min and serum was separated by centrifugation at 3000 rpm for 10 min (Eppendorf MiniSpin centrifuge, Germany). The samples were stored at −80 °C until analysis. All animal procedures were approved by the First Ethical Committee on Animal Experimentation in Krakow, Poland (no. 270/2019). 

#### 3.5.1. Analytical Method

Concentrations of compound 14 in mouse brains and serum were measured by liquid chromatography tandem mass spectrometry (LC-MS/MS) method using SCIEX QTRAP 4500 triple quadrupole mass spectrometer coupled to Excion LC AC HPLC system (both from Danaher Corporation, Washington, DC, USA). Chromatographic separation was carried out on the Hypersil GoldTM C18 analytical column (2.1 × 50 mm, 3 µm, Thermo Scientific, USA) with the oven temperature set at 40 °C. The mobile phase containing 0.1% formic acid in acetonitrile (A) and 0.1% formic acid in water (B) was delivered at the flow rate of 0.4 µL/min in the gradient mode. The initial mobile phase composition was 95% B for the first 2 min with a linear gradient to 5% B in the next 2 min, then isocratic mode for 2 min with the following rapid change back to 95% B in 0.1 min. The remaining time of elution was set at 95% B. The whole HPLC operation lasted 10 min. Electrospray ionization (ESI) in the positive ion mode was used for ion production. The tandem mass spectrometer was operated at unit resolution in the selected reaction monitoring mode (SRM), monitoring the transition of the protonated molecular ions *m*/*z* 489 to 132 (CE = 65 eV) and *m*/*z* 489 to 231 (CE = 29 eV) for compound 14 (first pair was used as a quantifier and the second for the identity verification qualifier) and *m*/*z* 436 to 235 (CE = 42 eV) for an internal standard (valsartan). The mass spectrometric conditions were optimized by continuous infusion of the standard solution at the rate of 7 μL/min using a Harvard infusion pump. The ion source temperature was maintained at 450 °C and the ion spray voltage was set at 5500 V. The curtain gas (CUR) was set at 40 psi and the collision gas (CAD) at medium. Data acquisition and processing were accomplished using the Analyst version 1.7 software. The calibration curves were constructed by plotting the ratio of the peak area of the studied compound to internal standard (IS) versus drug concentration and generated by weighted (1/x·x) linear regression analysis. The validated quantitation ranges for this method were within the expected concentration ranges, namely, from 0.1 to 20 µg/mL of serum and from 0.05 to 10 µg/g of brain tissue with accuracy from 93.73 to 104.29% and from 88.33 to 107% for serum and brain, respectively. No significant matrix effect was observed and there were no stability-related problems during the routine analysis of the samples. 

#### 3.5.2. Standard Solutions

The stock solution of compound **14** was prepared in methanol at the concentration of 1 mg/mL. Working standard solutions were prepared in the same solvent, by the serial dilution of the stock solution, at the following concentrations: 1, 2.5, 5, 10, 50, 100, and 200 µg/mL for serum analysis and 0.1, 0.25, 0.5, 1, 5, 10, and 20 µg/mL for analysis of brain homogenates. To prepare samples for calibration curve, 45 μL of matrix (plasma or brain homogenate) was spiked with 5 μL of standards working solution at appropriate concentration level and vortexed for 10 s.

#### 3.5.3. Sample Preparation

The brains were homogenized in distilled water at the ratio of 1:4 (*w/v*) with a tissue homogenizer LabGEN 125 (Cole-Parmer, Vernon Hills, IL, USA). Brain homogenates or serum samples (50 µL) were deproteinized with 150 μL of 0.1% formic acid in acetonitrile with an addition of internal standard, shaken for 10 min (IKA Vibrax VXR, Germany) and then centrifuged for 5 min at the speed of 8000× *g* (Eppendorf MiniSpin centrifuge, Germany). Serum supernatant was additionally diluted 10 times with the deproteinization solution. Then, brain or serum supernatants were transferred into the autosampler vials. The autosampler temperature was maintained at 15 °C and a sample volume of 2 μL was injected into the LC-MS/MS system.

### 3.6. In Vitro Pharmacology and ADME-Tox Studies

#### 3.6.1. Radioligand Binding/Functional Assays

Binding/functional studies were carried out commercially in Cerep Laboratories (Poitiers, France) and Eurofins Panlabs Discovery Services Taiwan, Ltd. (New Taipei City, Taiwan) using testing procedures reported previously. The general information is listed in Supplementary Materials—Table S2.

#### 3.6.2. In Vitro Electrophysiological Studies

The methodology of slice preparation, slice preincubation, and patch-clamp technique was the same as in our previous study [52]. In this study, however, we used different recording solutions. The composition of pipette solution was (in mM) CsF (110), NaCl (7), EGTA (3), HEPES-Cl (10), MgCl_2_ (2), and Na_2_ATP (4) at pH 7,4. The composition of extracellular solution was (in mM) NaCl (30), choline chloride (90), TEA-Cl (30), CaCl_2_ (2), MgCl_2_ (2), glucose (15), HEPES (10), LaCl_3_ (0.001), and CdCl_2_ (0.4) at pH 7.4. Compound **14** was tested at concentration of 1 µM.

#### 3.6.3. In Vitro Toxicity Studies

All applied in vitro methods for assessment of compound **14**’s toxicity were described previously. The luminescent CYP3A4 P450-Glo™ and CYP2D6 P450-Glo™ assays and protocols were provided by Promega^®^ (Madison, WI, USA). Compound **14** was tested in triplicate at the final concentrations from 0.1 to 25 μM. Hepatoma HepG2 (ATCC^®^ HB-8065™) and neuroblastoma SH-SY5Y (ATCC^®^ CRL-2266™) cell lines were obtained directly from ATCC^®^ (American Type Culture Collection, Manassas, VA, USA). The CellTiter 96^®^ AQueous Non-Radioactive Cell Proliferation Assay (MTS) was purchased from Promega (Madison, WI, USA). Compound **14** was incubated with cells in four repetitions in the concentration range 0.1–100 μM. The additional test was performed with SH-SY5Y cells in the concentration range 0.001–1 µM. During the toxicity examination, the luminescent signal and the absorbance (490 nm) were measured by using a microplate reader EnSpire PerkinElmer (Waltham, MA, USA).

## 4. Conclusions

In the current study, we generated a series of water-soluble hydrochlorides of the pyrrolidine-2,5-dione derivatives targeted as potential anticonvulsants with additional antinociceptive properties. The preclinical data in mice revealed that these compounds exhibited potent protection and showed broad-spectrum activity in different animal models of seizures, such as the MES, 6 Hz (32/44 mA), and/or *sc*PTZ seizure tests. The most beneficial anticonvulsant properties and safety profile revealed compound **14**. In addition, compound **14** showed potent effectiveness by decreasing pain responses in the formalin-induced tonic pain, the capsaicin-induced neurogenic pain, as well as in the OXPT-induced neuropathic pain in mice. Notably, compound **14** also displayed anti-inflammatory activity in the model of carrageenan-induced aseptic inflammation. The in vitro ADME-Tox studies revealed that compound **14** inhibited CYP3A4 activity, but only at the highest used concentration, showed negligible influence on CYP2D6, had a very low hepatotoxic potential, and notably increased the viability of SH-SY5Y cells. Overall, the obtained results indicated that compound **14** seems to be an interesting candidate for further and more advanced preclinical development in epilepsy and, potentially, neuropathic pain.

## 5. Patents

The authors declare no competing financial interest. The results are subject of the international WIPO patent application, no. WO 2020/145831 (publication date 16 July 2020).

## Figures and Tables

**Table 2:** The quantitative data in the 6 Hz (44 mA) test in mice *i.p*.

Compound	TPE (h) ^a^	ED_50_ 6 Hz (44 mA) (mg/kg) ^b^	TD_50_ (mg/kg) ^c^	PI (TD_50_/ED_50_) ^d^
**14**	0.5	63.2 (37.6–106.4)	168.7 (146.3–194.5)	2.7
**I** *	0.5	73.2 (57.4–93.4)	195.7 (132.7–288.6)	2.7
**LCS** *	0.5	6.9 (5.4–8.6)	46.2 (44.5–48.0)	6.7
**LEV** *	1.0	>500	>500	n.c.
**VPA** *	0.5	183.1 (143.5–233.7)	430.7 (407.9–454.9)	2.3

Values in parentheses are 95% confidence intervals [20].^**a**^ Time to peak effect. **^b^** ED_50_ (6 Hz psychomotor seizure test, 44 mA).^**c**^ TD_50_ (acute neurological toxicity determined in the rotarod test).^**d**^ Protective index (TD_50_/ED_50_). * Data for racemate **I** (compound **KA-104**) and reference ASDs (LCS, LEV, VPA) have been published previously [7].

**Table 3:** In vitro binding and functional assays for compound **14** and **(C1-*R*)-32**.

	% Inhibition of Control-Specific Binding (Concentration µM)	
Compd	TTX-sensitive Na^+^ channel *	Cav_1.2_ channel (dihydropyridine site, antagonist radioligand) *
**14**	90.3 (50) 31.0 (10)	75.4 (50) 6.0 (10)
(**C1-*R*)-32**	101.9 (50) 33.4 (10)	88.9 (50) 18.6 (10)
	**% Inhibition of Control Agonist Response (Concentration µM)**	
Compd	TRPV1 (VR1) (*h*) (antagonist effect) **	Cav_1.2_ (*h*) calcium ion channel cell based antagonist calcium flux assay **
**14**	88.8 (50) 17.3 (10)	29.0 (10)
(**C1-*R*)-32**	44.6 (50) 8.5 (10)	38.0 (10)

Results interpretation; activity > 50% means significant effects of the test substance; activity 25–50% indicates moderate effect; results < 25% means non-active. All studies were carried out commercially in Eurofins Cerep SA (Celle l’Evescault, France) or in Panlabs Discovery Services Taiwan, Ltd. (New Taipei City, Taiwan). Source: * Rat cerebral cortex; ** human recombinant HEK-293 cells.

**Table 4:** Pharmacokinetic parameters of compound **14** following *i.p.* administration of a dose of 40 mg/kg calculated using the non-compartmental analysis.

Parameter	Serum	Brain
t_max_ (min)	5	15
C_max_ (µg/mL(g))	20.06	5.32
λ_z_ (min^−1^)	0.011	0.011
t_0.5λz_ (min)	64.65	66.14
V/F (L/kg)	2.57	-
CL/F (L/min/kg)	0.027	-
AUC_0-∞_ (µg⋅min/mL(g))	1451.22	413.17
MRT (min)	93.46	89.17

## Supplementary Materials

The following are available online at https://www.mdpi.com/article/10.3390/ijms222313092/s1, synthetic pathway of compound (**C1-*S***)**-****31**, anticonvulsant screening data, binding/functional assay information, UPLC/MS traces, chiral HPLC chromatograms, and ^1^H NMR, ^13^C NMR spectra for target compounds.

## Abbreviations

ADME-Tox	Absorption, distribution, metabolism, excretion, toxicity
AEDs	Antiseizure drugs
CCCP	3-Chlorophenylhydrazone
DCC	Dicyclohexylcarbodiimide
DCM	Dichloromethane
DX	Doxorubicin
ETX	Ethosuximide
HLMs	Human liver microsomes
HMDS	Hexamethyldisilazane
6 Hz	6 Hz seizure test
LCS	Lacosamide
LEV	Levetiracetam
MeCN	Acetonitrile
MES	Maximal electroshock seizure test
MeOH	Methanol
OXPT	Oxaliplatin
PI	Protective index (TD_50_/ED_50_)
*sc*PTZ	Subcutaneous pentylenetetrazole seizure test
SV2A	Synaptic vesicle glycoprotein 2A
TFA	Trifluoroacetic acid
THF	Tetrahydrofuran
TPE	Time of peak effect
TRPV1	Transient receptor potential cation channel vanilloid type 1
VPA	Valproic acid
