# Supplementary material for: Identification of New Compounds with Anticonvulsant and Antinociceptive Properties in a Group of 3-substituted (2,5-dioxo-pyrrolidin-1-yl)(phenyl)-Acetamides
Source: Int J Mol Sci. 2021 Dec 3;22(23):13092. doi: 10.3390/ijms222313092 (PMC8658016; doi:10.3390/ijms222313092)
Supplement: Supplementary file 1 [file ijms-22-13092-s001.zip › ijms-1482227-supplementary.pdf]

Supplementary Materials for

Identification of new compounds with anticonvulsant and antinociceptive properties in a group of 3-substituted (2,5-dioxopyrrolidin-1-yl)(phenyl)-acetamides

Michał Abram [1], Marcin Jakubiec [1], Anna Rapacz [2], Szczepan Mogilski [2], Gniewomir Latacz [3], Bartłomiej Szulczyk [4], Małgorzata Szafarz [5], Katarzyna Socała [6], Dorota Nieoczym [6], Elżbieta Wyska [5], Piotr Wlaź [6], Rafał M. Kamiński [1], and Krzysztof Kamiński [1,*]

[1] Department of Medicinal Chemistry, Faculty of Pharmacy, Jagiellonian University Medical College, Medyczna 9, 30-688 Krakow, Poland; michal.abram@uj.edu.pl (M.A.); marcin.jakubiec@doctoral.uj.edu.pl (M.J.); rafa1.kaminski@uj.edu.pl (R.M.K.)
[2] Department of Pharmacodynamics, Faculty of Pharmacy, Jagiellonian University Medical College, Medyczna 9, 30-688 Krakow, Poland; a.rapacz@uj.edu.pl (A.R.); szczepan.mogilski@uj.edu.pl (S.M.)
[3] Department of Technology and Biotechnology of Drugs, Faculty of Pharmacy, Jagiellonian University Medical College, Medyczna 9, 30-688 Krakow, Poland; gniewomir.latacz@uj.edu.pl
[4] Department of Pharmacodynamics, Centre for Preclinical Research and Technology, Medical University of Warsaw, Banacha 1B, 02-097 Warsaw, Poland; bszulczyk@wum.edu.pl
[5] Department of Pharmacokinetics and Physical Pharmacy, Faculty of Pharmacy, Jagiellonian University Medical College, Medyczna 9, 30-688 Krakow, Poland; malgorzata.szafarz@uj.edu.pl (M.S.); mfwyska@cyf-kr.edu.pl (E.W.)
[6] Department of Animal Physiology and Pharmacology, Institute of Biological Sciences, Faculty of Biology and Biotechnology, Maria Curie-Skłodowska University, Akademicka 19, 20-033 Lublin, Poland; k.socala@poczta.umcs.lublin.pl (K.S.); dorota.nieoczym@mail.umcs.pl (D.N.); piotr.wlaz@mail.umcs.pl (P.W.)
* Correspondence: k.kaminski@uj.edu.pl; Tel.: +48-12-620-54-59

Table of contents

Scheme S1. Synthetic pathway of compound **(C1-*S*)-31**. 2

Table S1. Anticonvulsant activity screening data in the MES, 6 Hz (32 mA) and *sc*PTZ seizure models in mice *i.p.* (dose of 100 mg/kg) at 0.5 h post compound administration. 3

Table S2. Radioligand binding and functional assays for **14** .and **(C1-*R*)-32**. 3

References 4

UPLC/MS traces for target final compounds 5

^{1}H NMR, ^{13}C NMR spectra for final compounds 15

Chiral HPLC chromatograms 35

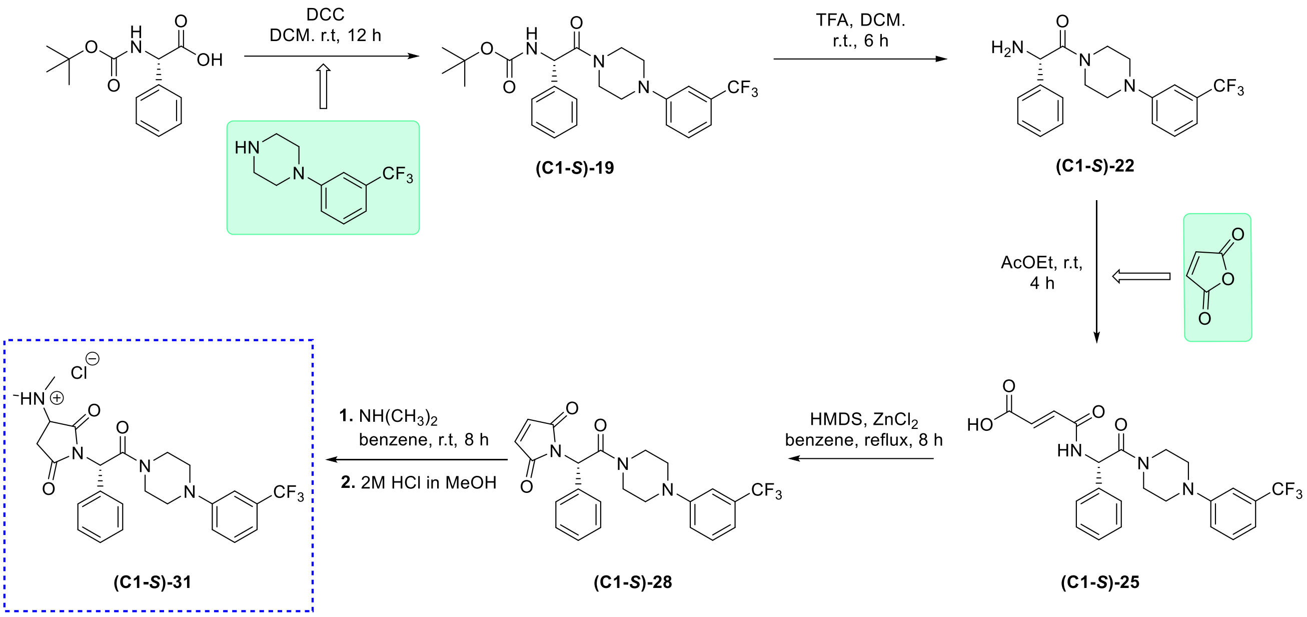

Scheme S1. Synthetic pathway of compound **(C1-*S*)-31**.

Table S1. Anticonvulsant activity screening data in the MES, 6 Hz (32 mA) and *sc*PTZ seizure models in mice *i.p.* (dose of 100 mg/kg) at 0.5 h post compound administration.

Compd	A	R	MES	6 Hz (32 mA)	*sc*PTZ
**13**	-$NHCH_3$	3-CF_3	1/4	**2/4**	**2/4**
**14**	-$N(CH_3)_2$	3-CF_3	**4/4**	**3/4**	**2/4**
**15**	-$N(C_2H_5)_2$	3-CF_3	**4/4**	**3/4**	1/4
**16**	morpholine	3-CF_3	0/4	0/4	1/4
**17**	-$N(CH_3)_2$	3-OCF_3	**3/4**	**2/4**	**3/4**
**18**	-$N(CH_3)_2$	3-SCF_3	1/4	1/4	1/4
**(C1-*R*)-31**	-$N(CH_3)_2$	3-CF_3	**4/4**	**3/4**	**3/4**
**(C1-*S*)-31**	-$N(CH_3)_2$	3-CF_3	1/4	**2/4**	1/4
**(C1-*R*)-32**	-$N(CH_3)_2$	3-OCF_3	**4/4**	**3/4**	**3/4**
**(C1-*R*)-33**	-$N(CH_3)_2$	3-SCF_3	1/4	1/4	1/4

Data for the most potent compounds have been highlighted in bold for easier interpretation.
[a] Data indicate: number of mice protected / number of mice tested. The animals were examined at one pretreatment time – 0.5 h.
MES – the maximal electroshock seizure test.
6 Hz – the psychomotor 6 Hz seizure test (current intensity of 32 mA).
*sc*PTZ – the subcutaneous pentylenetetrazole seizure test.

Table S2. Radioligand binding and functional assays for **14** .and **(C1-*R*)-32**.

Assays were performed commercially in Eurofins Cerep SA (Celle l'Evescault, France) or Eurofins Panlabs Discovery Services Taiwan, Ltd. (New Taipei City, Taiwan), using procedures describe elsewhere:

**Binding studies**	Ref.
Na^+ channel (site 2)	[1]
L-type Ca^{2+}(diltiazem site, antagonist radioligand)	[2]
**Functional studies**	
TRPV1 (VR1) *(h)* (antagonist effect)	[3]
$Cav_{1.2}$ (L-type) *(h)* calcium ion channel cell based antagonist calcium flux assay	[4,5]

References

1. Brown, G.B. 3H-Batrachotoxinin-A Benzoate Binding to Voltage-Sensitive Sodium Channels: Inhibition by the Channel Blockers Tetrodotoxin and Saxitoxin. *J. Neurosci.* **1986**, *6*, 2064–2070, doi:10.1523/JNEUROSCI.06-07-02064.1986.
2. Schoemaker, H.; Langer, S.Z. [3H]Diltiazem Binding to Calcium Channel Antagonists Recognition Sites in Rat Cerebral Cortex. *Eur. J. Pharmacol.* **1985**, *111*, 273–277, doi:10.1016/0014-2999(85)90768-x.
3. Phelps, P.T.; Anthes, J.C.; Correll, C.C. Cloning and Functional Characterization of Dog Transient Receptor Potential Vanilloid Receptor-1 (TRPV1). *Eur. J. Pharmacol.* **2005**, *513*, 57–66, doi:10.1016/j.ejphar.2005.02.045.
4. Sirenko, O.; Crittenden, C.; Callamaras, N.; Hesley, J.; Chen, Y.-W.; Funes, C.; Rusyn, I.; Anson, B.; Cromwell, E.F. Multiparameter in Vitro Assessment of Compound Effects on Cardiomyocyte Physiology Using IPSC Cells. *J. Biomol. Screen.* **2013**, *18*, 39–53, doi:10.1177/1087057112457590.
5. Xia, M.; Imredy, J.P.; Koblan, K.S.; Bennett, P.; Connolly, T.M. State-Dependent Inhibition of L-Type Calcium Channels: Cell-Based Assay in High-Throughput Format. *Anal. Biochem.* **2004**, *327*, 74–81, doi:10.1016/j.ab.2004.01.003.

UPLC/MS traces for target final compounds

The purity of target final compounds determined by use of chromatographic UPLC method was ≥99%.

3-(Methylamino)-1-(2-oxo-1-phenyl-2-(4-(3-(trifluoromethyl)phenyl)piperazin-1-yl)ethyl)pyrrolidine-2,5-dione hydrochloride (13)

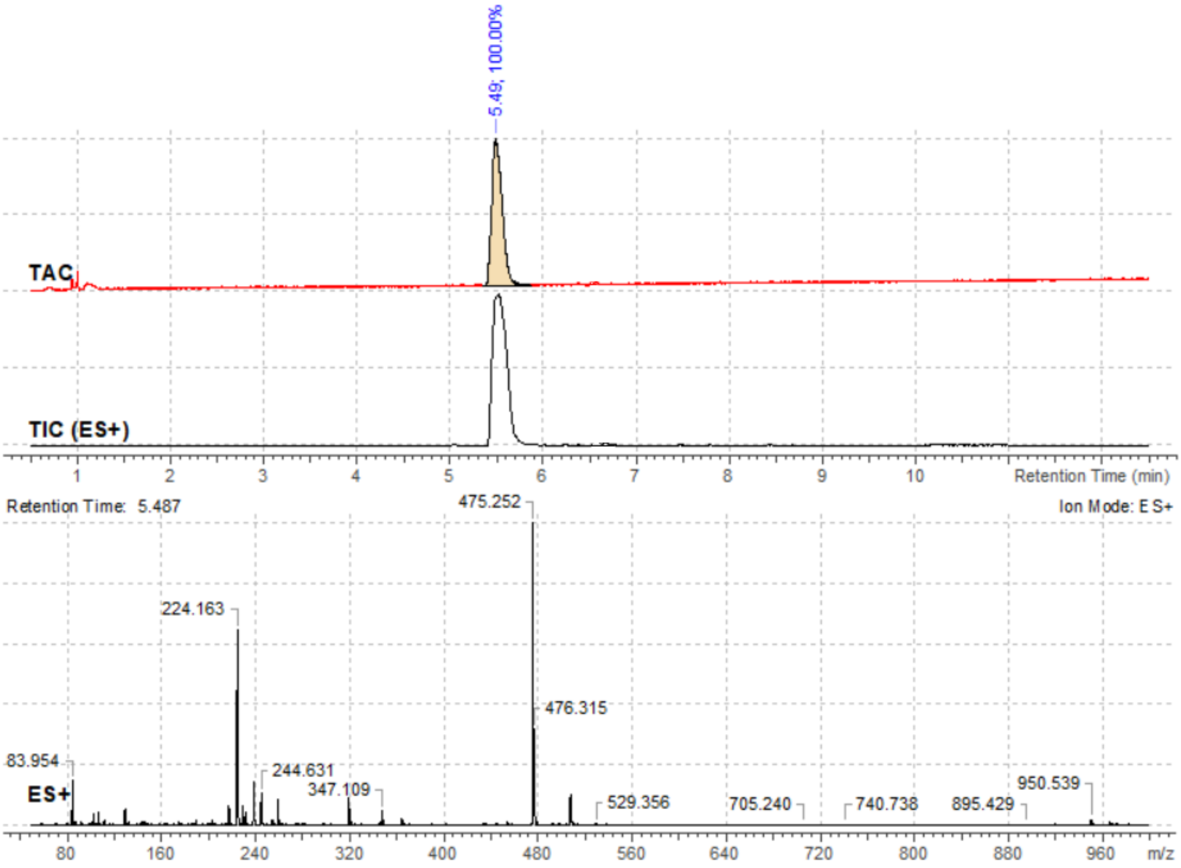

3-(Dimethylamino)-1-(2-oxo-1-phenyl-2-(4-(3-(trifluoromethyl)phenyl)-piperazin-1-yl)ethyl)pyrrolidine-2,5-dione hydrochloride (14)

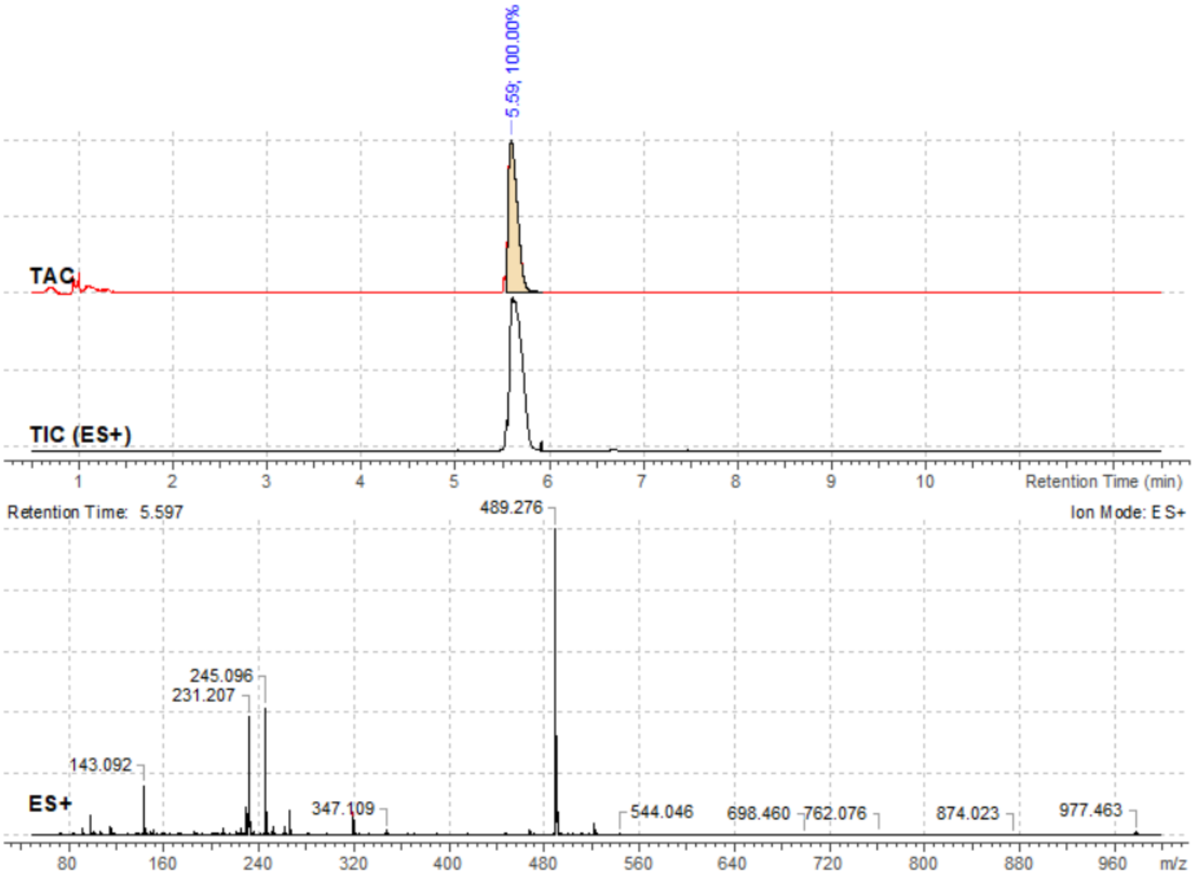

3-(Diethylamino)-1-(2-oxo-1-phenyl-2-(4-(3-(trifluoromethyl)phenyl)piperazin-1-yl)ethyl)pyrrolidine-2,5-dione hydrochloride (15)

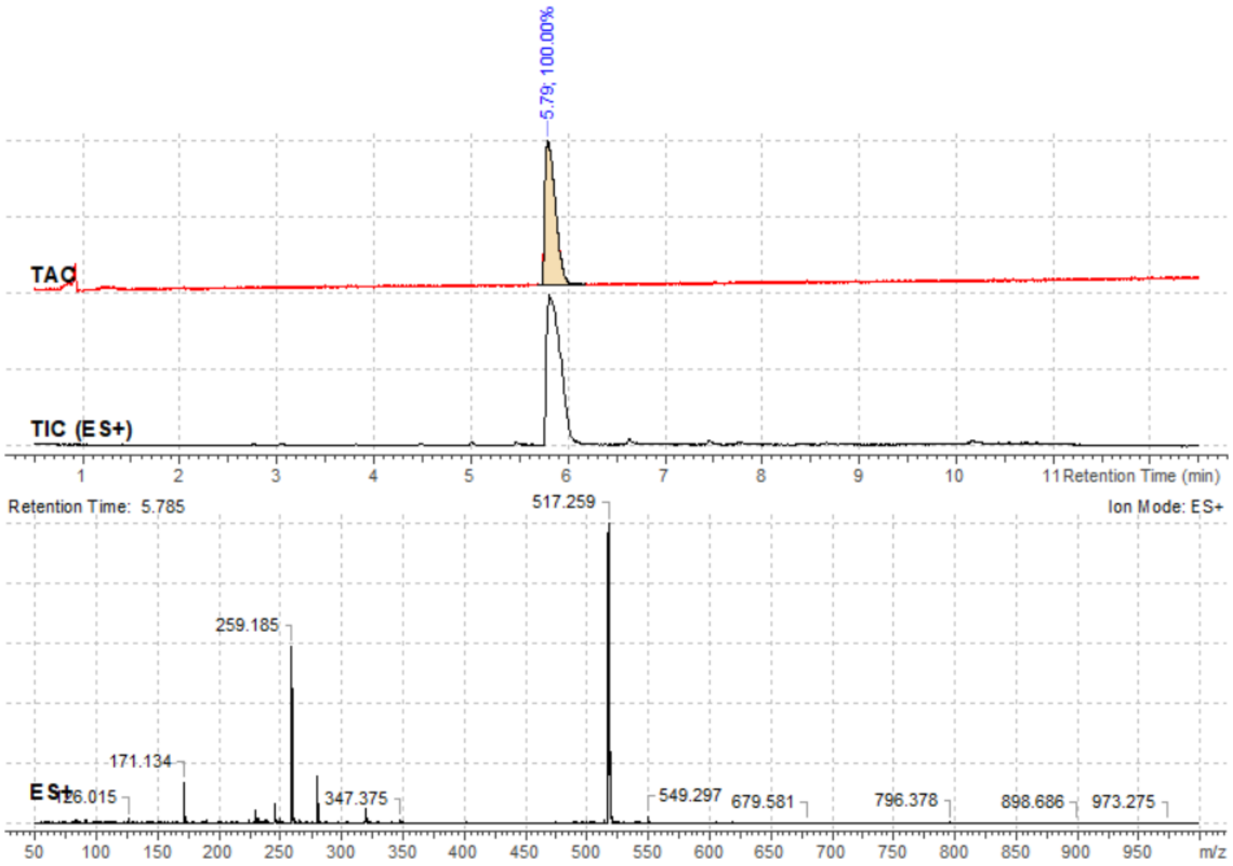

3-Morpholino-1-(2-oxo-1-phenyl-2-(4-(3-(trifluoromethyl)phenyl)piperazin-1-yl)ethyl)pyrrolidine-2,5-dione hydrochloride (16)

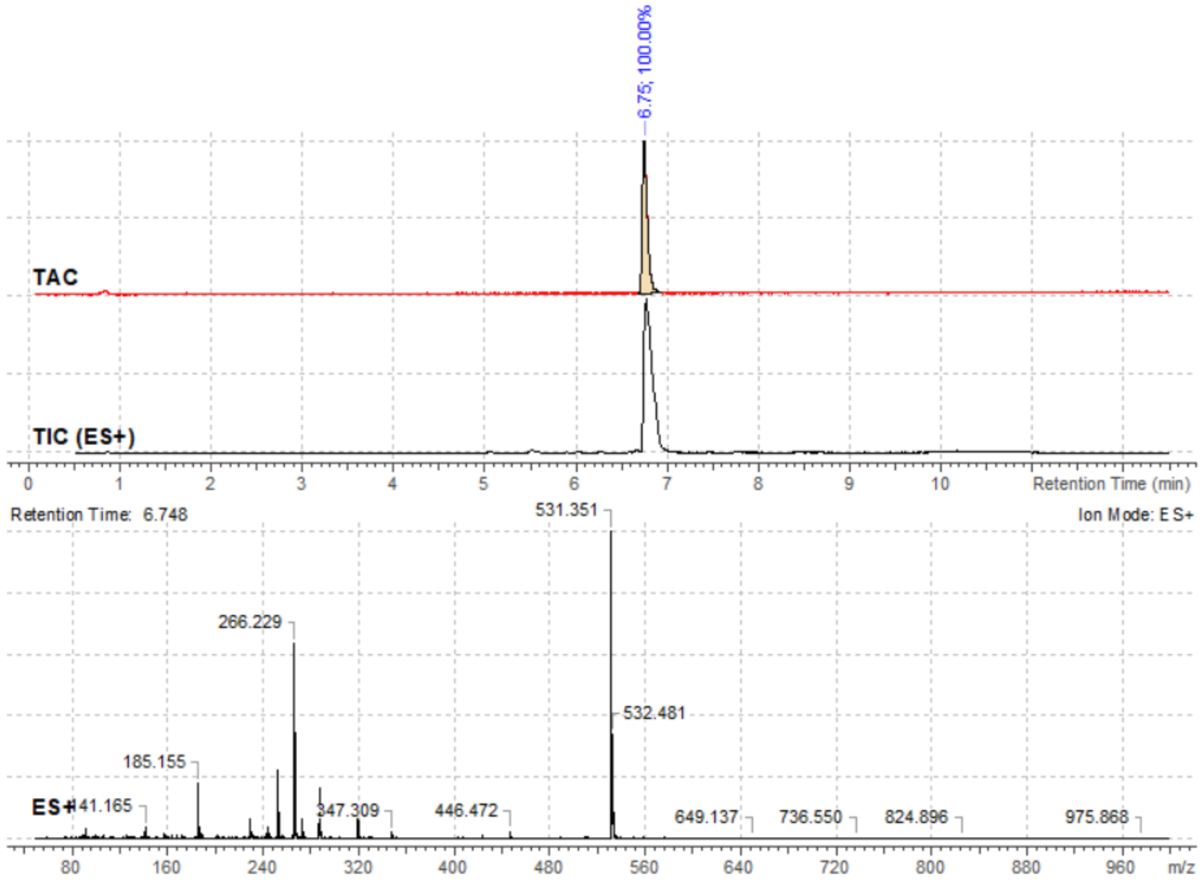

3-Dimethylamino-1-(2-oxo-1-phenyl-2-(4-(3-(trifluoromethoxy)phenyl)piperazin-1-yl)ethyl)pyrrolidine-2,5-dione hydrochloride (17)

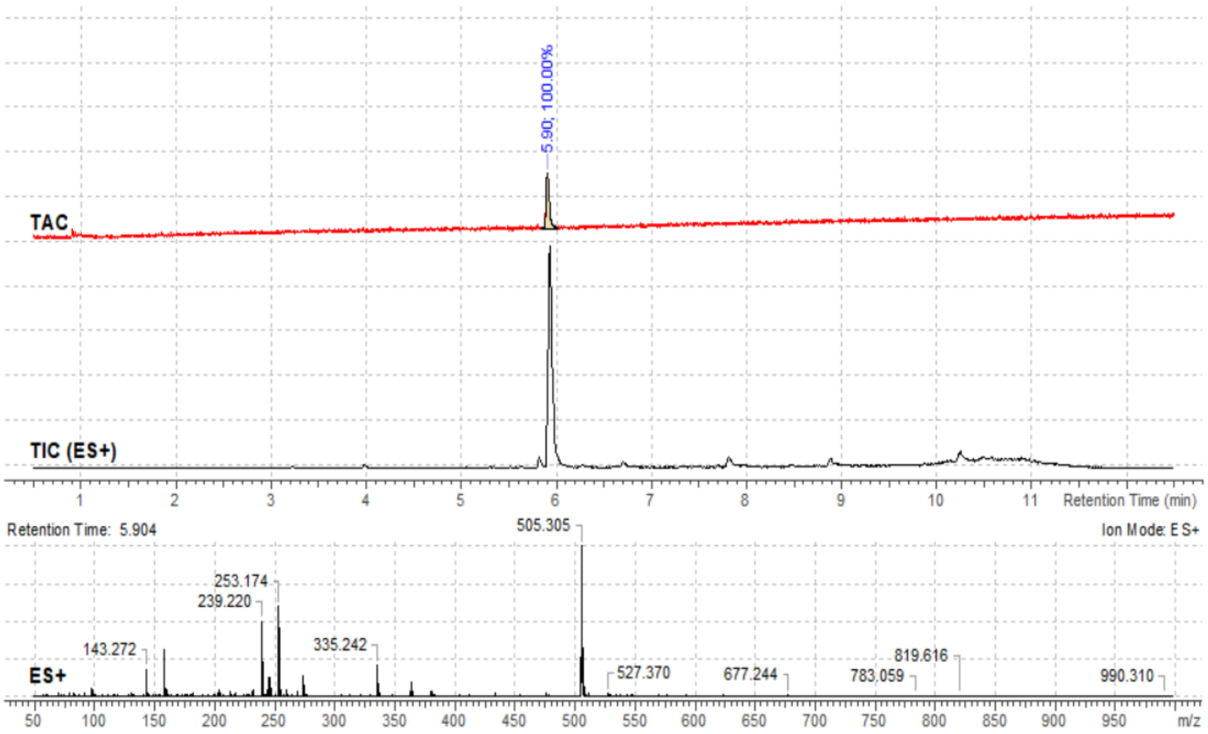

3-Dimethylamino-1-(2-oxo-1-phenyl-2-(4-(3-((trifluoromethyl)thio)phenyl)piperazin-1-yl)ethyl)pyrrolidine-2,5-dione hydrochloride (18)

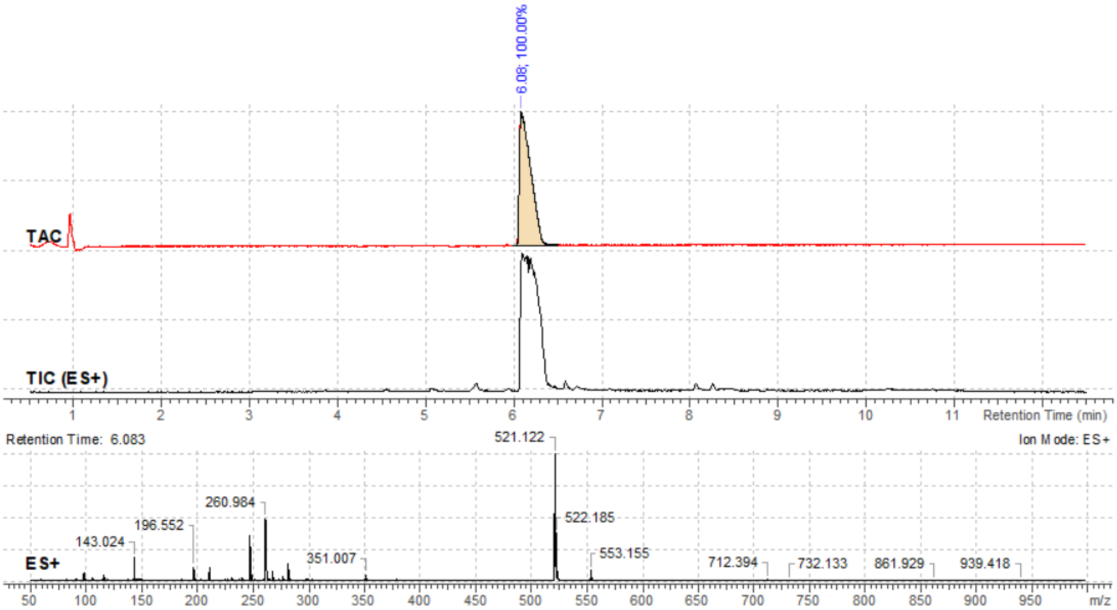

3-Dimethylamino-1-((*R*)-oxo-1-phenyl-2-(4-(3-(trifluoromethyl)phenyl)-piperazin-1-yl)ethyl)pyrrolidine-2,5-dione hydrochloride (C1-*R*)-31

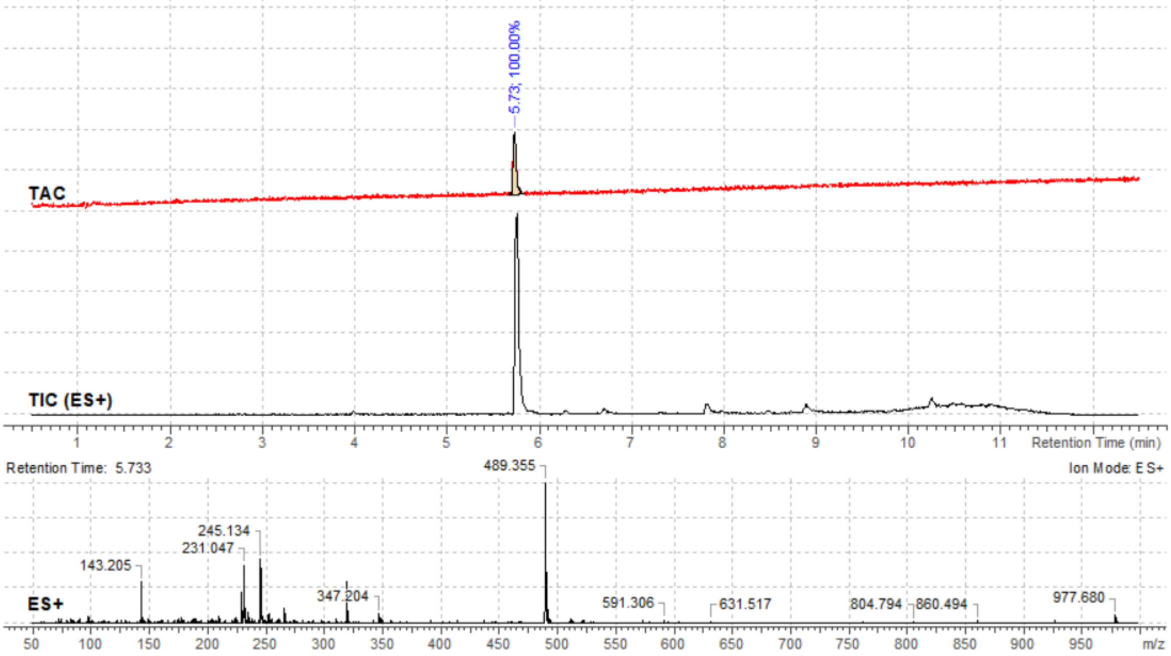

3-Dimethylamino-1-((*S*)-oxo-1-phenyl-2-(4-(3-(trifluoromethyl)phenyl)-piperazin-1-yl)ethyl)pyrrolidine-2,5-dione hydrochloride (C1-*S*)-31

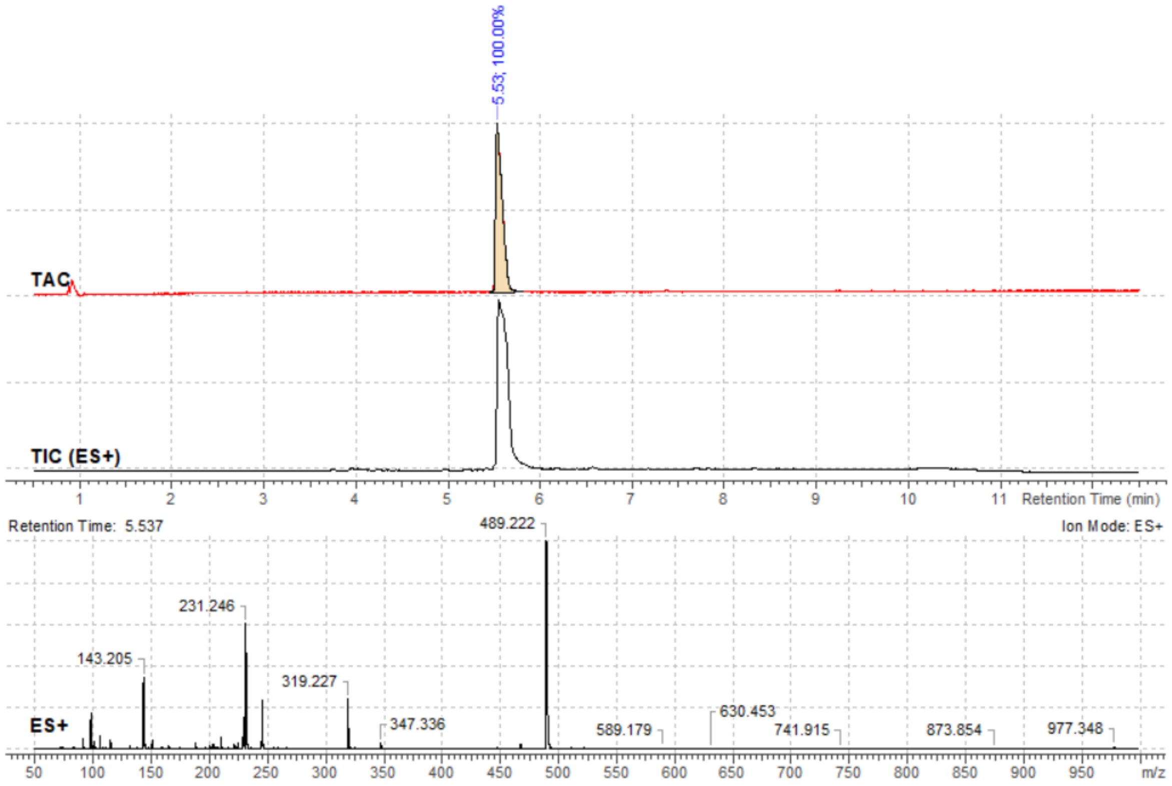

3-Dimethylamino-1-((*R*)-2-oxo-1-phenyl-2-(4-(3-(trifluoromethoxy)phenyl)piperazin-1-yl)ethyl)pyrrolidine-2,5-dione hydrochloride (C1-*R*)-32

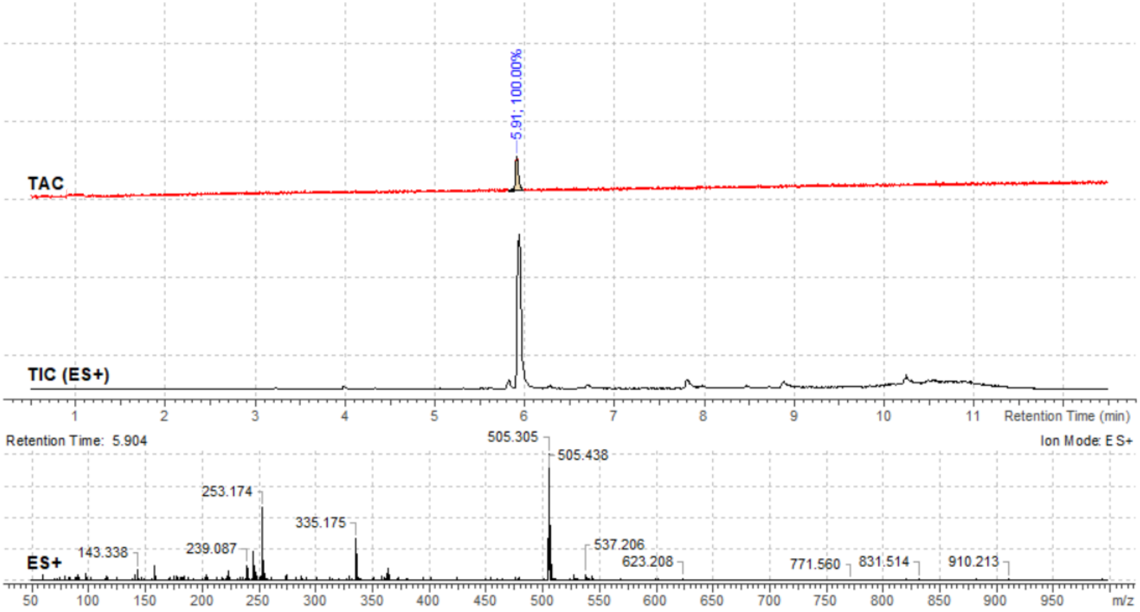

3-Dimethylamino-1-((*R*)-2-oxo-1-phenyl-2-(4-(3-((trifluoromethyl)thio)phenyl)piperazin-1-yl)ethyl)pyrrolidine-2,5-dione hydrochloride (C1-*R*)-33

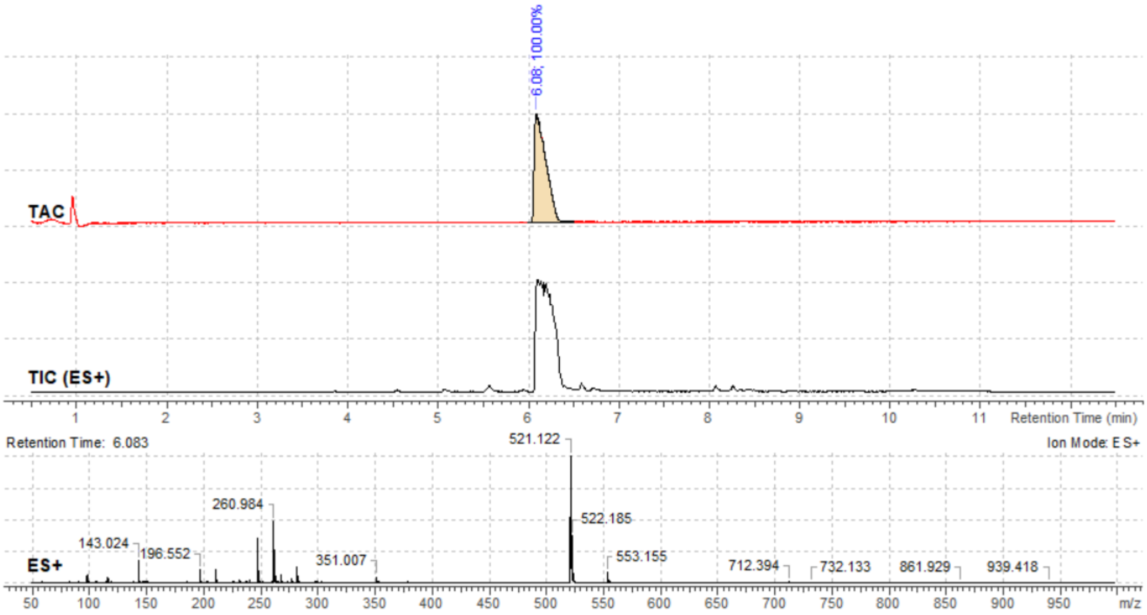

^{1}H NMR, ^{13}C NMR spectra for final compounds

3-(Methylamino)-1-(2-oxo-1-phenyl-2-(4-(3-(trifluoromethyl)phenyl)piperazin-1-yl)ethyl)pyrrolidine-2,5-dione (13)

^{1}H NMR

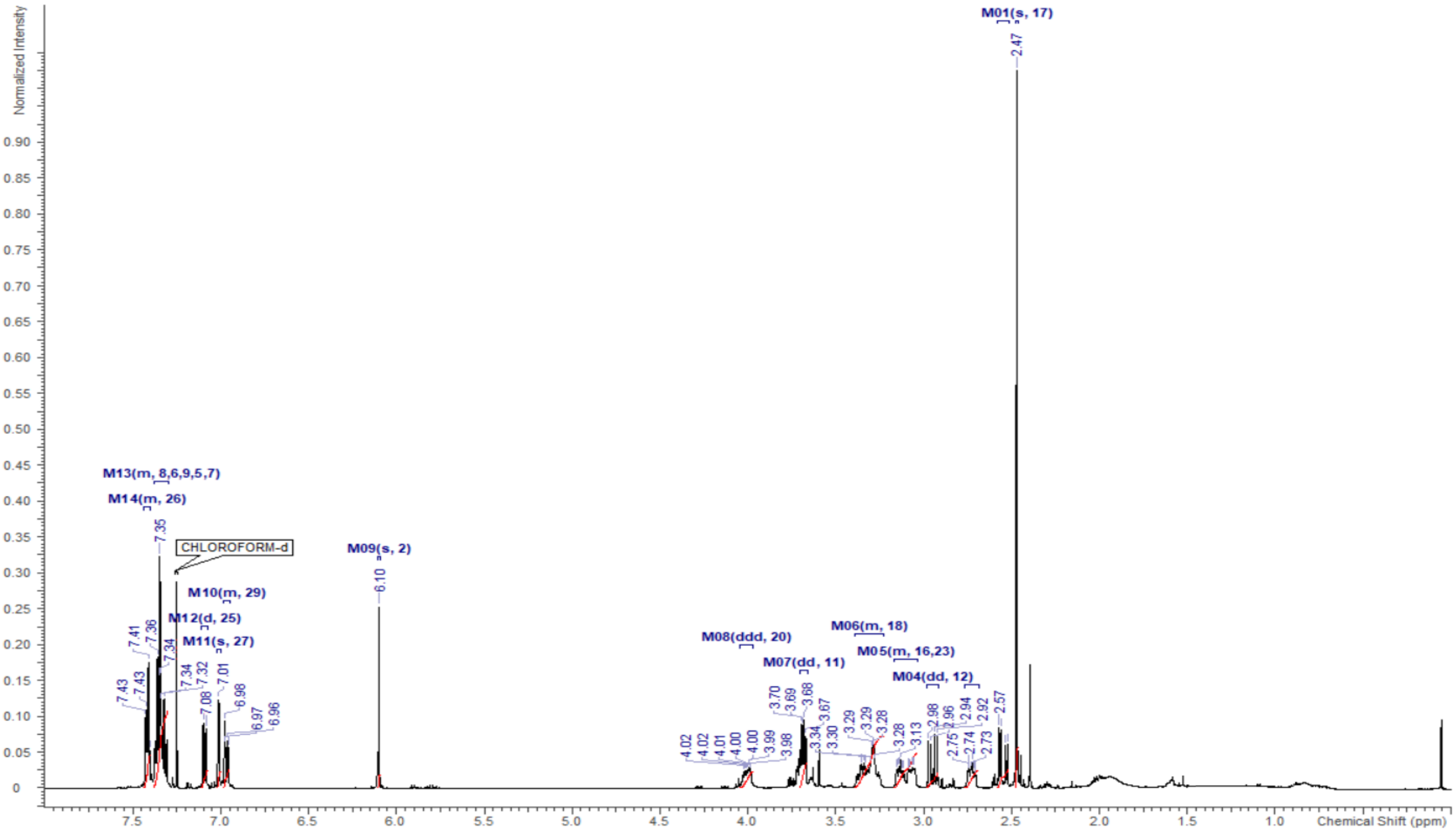

3-(Methylamino)-1-(2-oxo-1-phenyl-2-(4-(3-(trifluoromethyl)phenyl)piperazin-1-yl)ethyl)pyrrolidine-2,5-dione (13)

^{13}C NMR

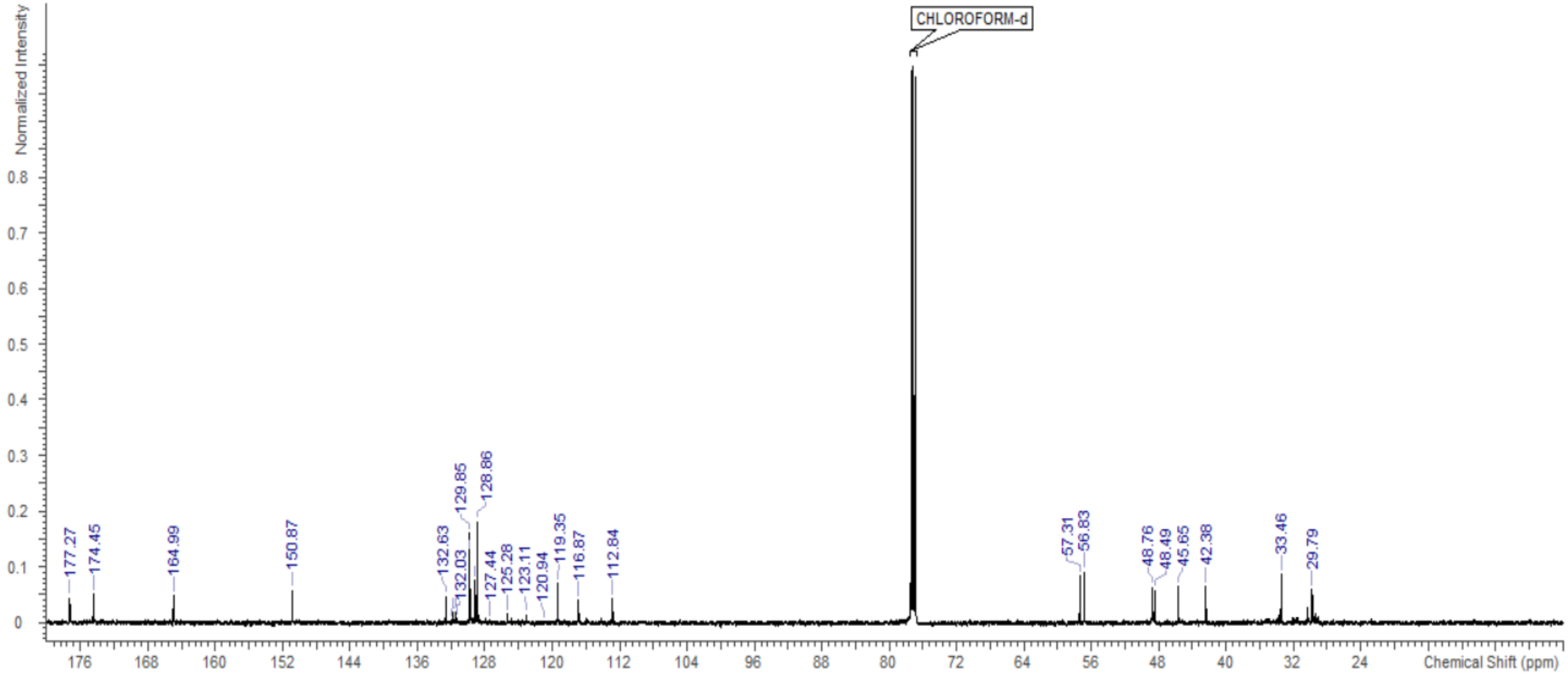

3-(Dimethylamino)-1-(2-oxo-1-phenyl-2-(4-(3-(trifluoromethyl)phenyl)-piperazin-1-yl)ethyl)pyrrolidine-2,5-dione (14)

^{1}H NMR

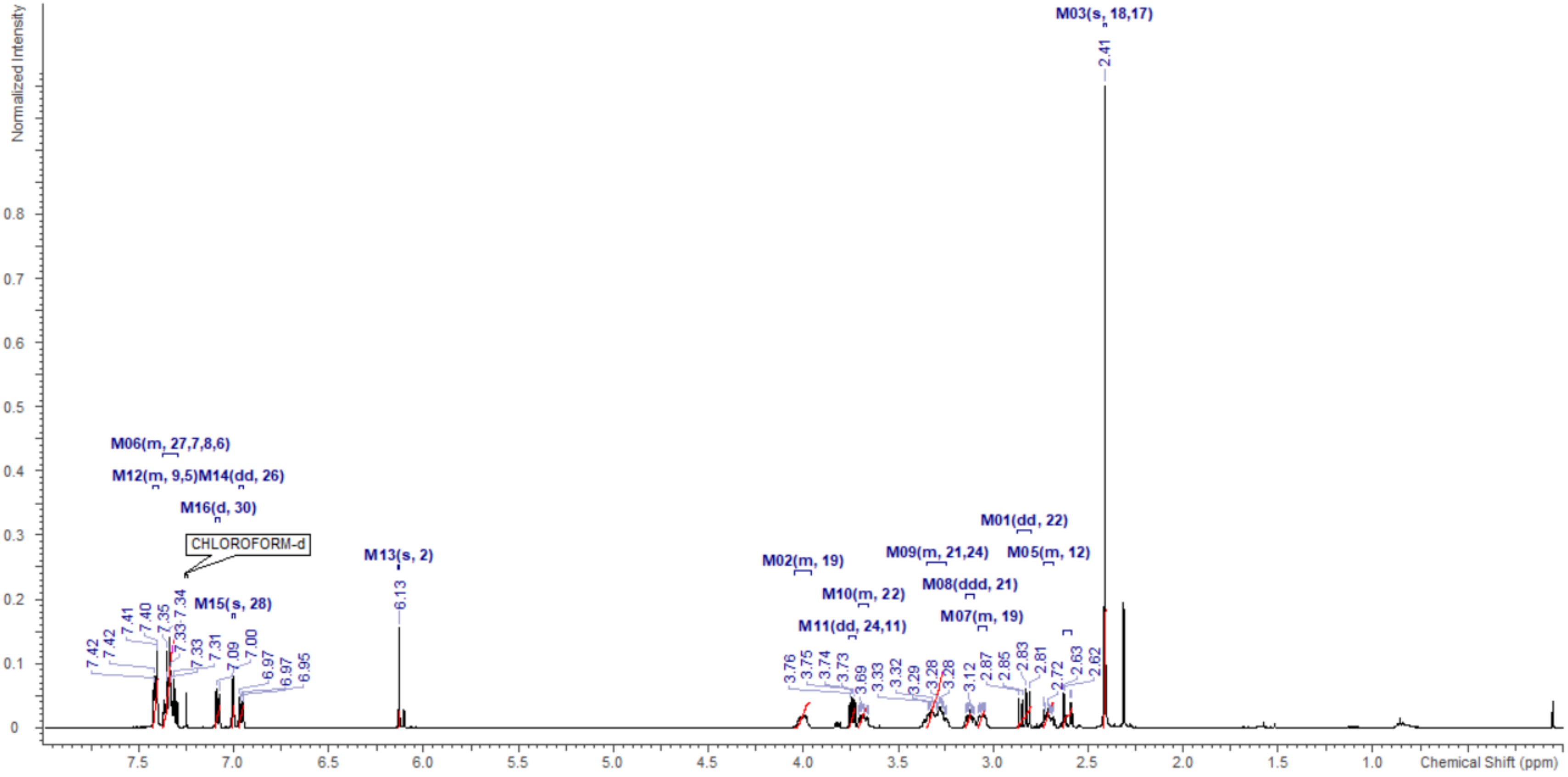

3-(Dimethylamino)-1-(2-oxo-1-phenyl-2-(4-(3-(trifluoromethyl)phenyl)-piperazin-1-yl)ethyl)pyrrolidine-2,5-dione (14)

^{13}C NMR

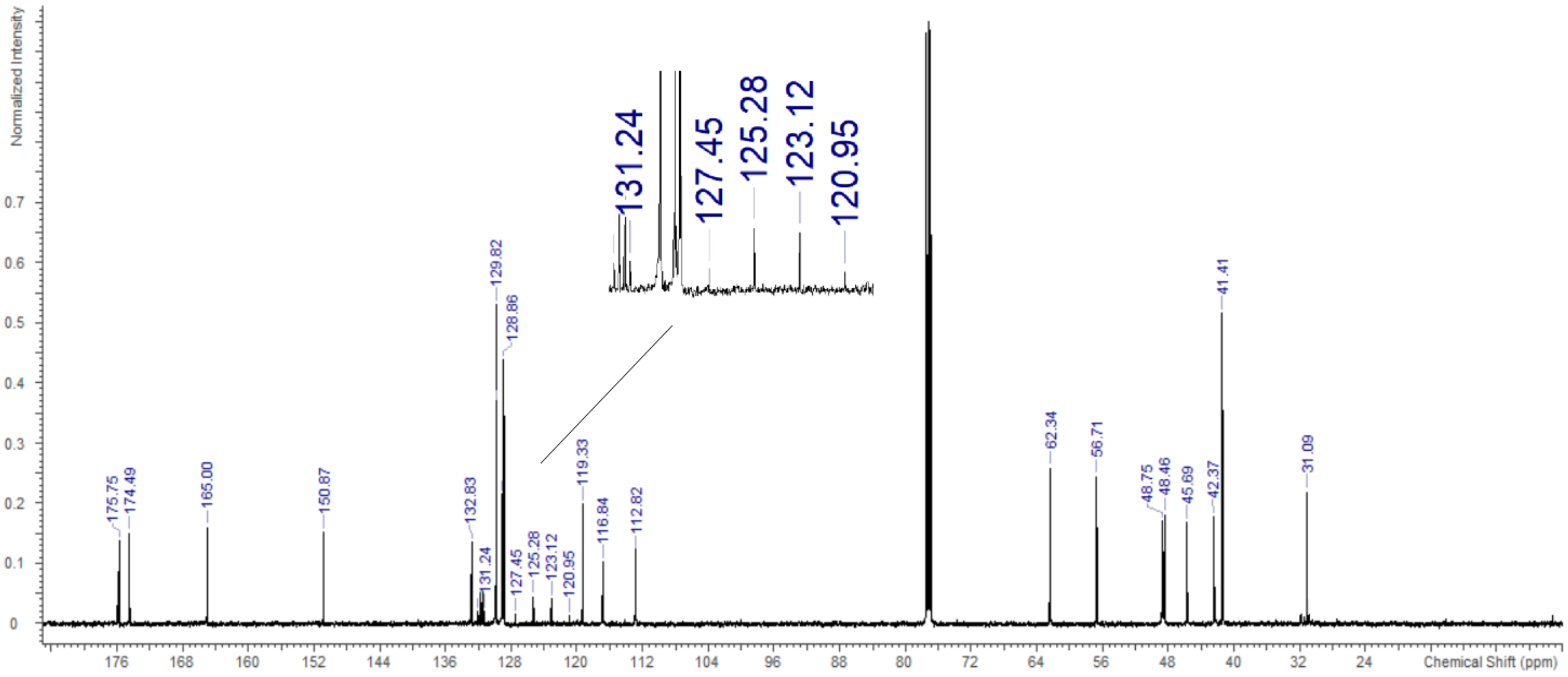

3-(Diethylamino)-1-(2-oxo-1-phenyl-2-(4-(3-(trifluoromethyl)phenyl)piperazin-1-yl)ethyl)pyrrolidine-2,5-dione (15)

^{1}H NMR

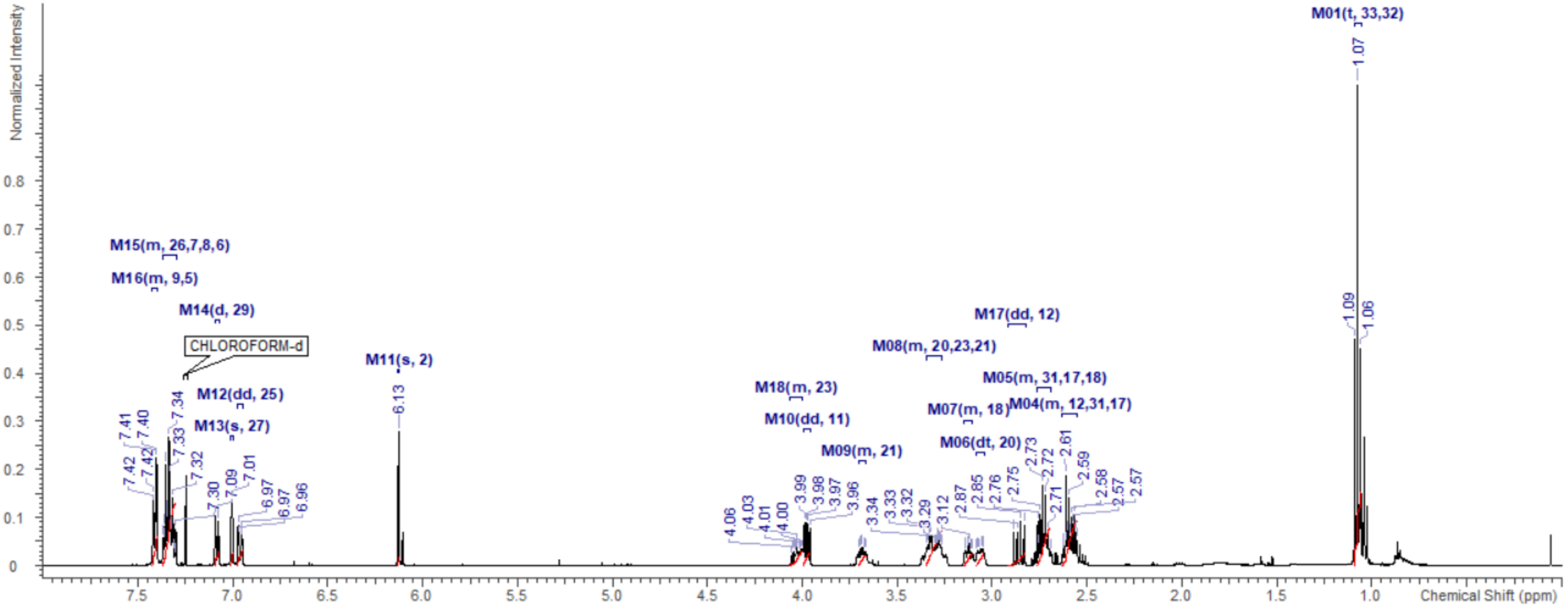

3-(Diethylamino)-1-(2-oxo-1-phenyl-2-(4-(3-(trifluoromethyl)phenyl)piperazin-1-yl)ethyl)pyrrolidine-2,5-dione (15)

^{13}C NMR

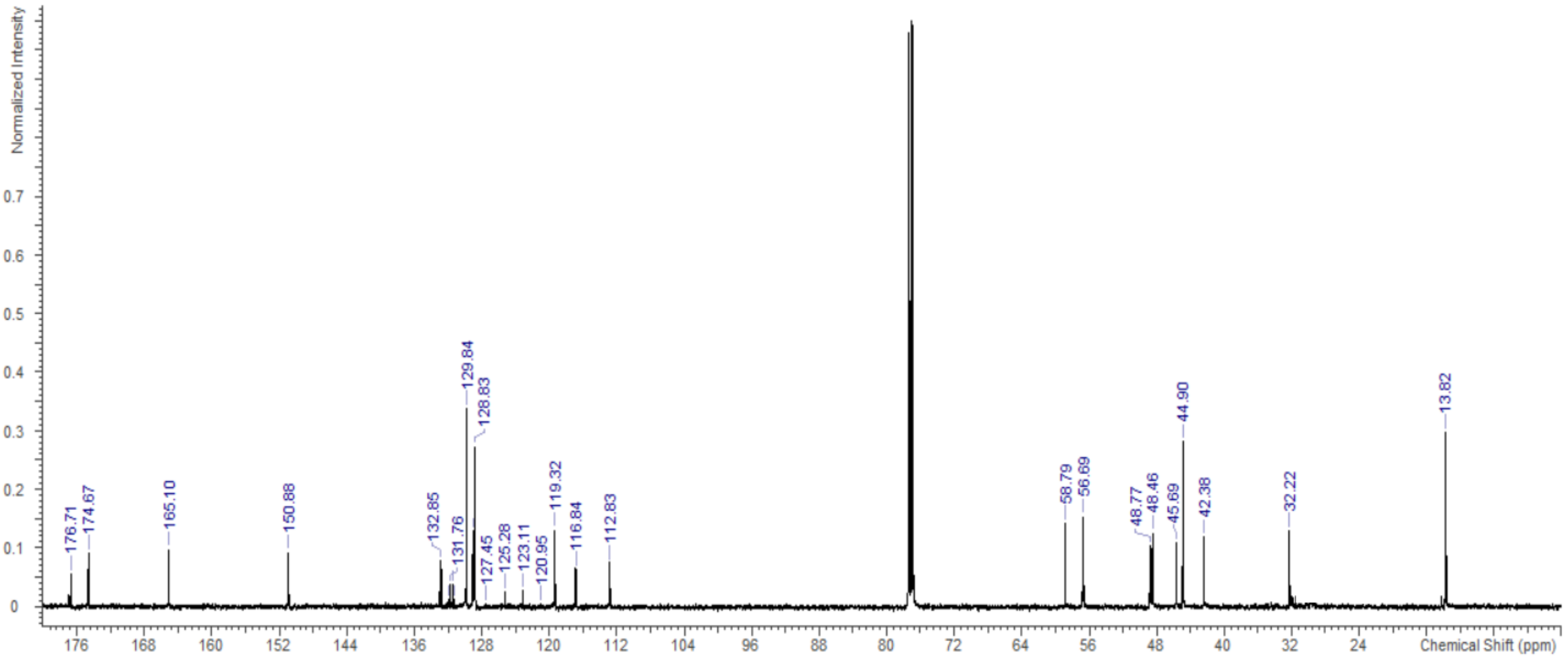

3-Morpholino-1-(2-oxo-1-phenyl-2-(4-(3-(trifluoromethyl)phenyl)piperazin-1-yl)ethyl)pyrrolidine-2,5-dione (16)

^{1}H NMR

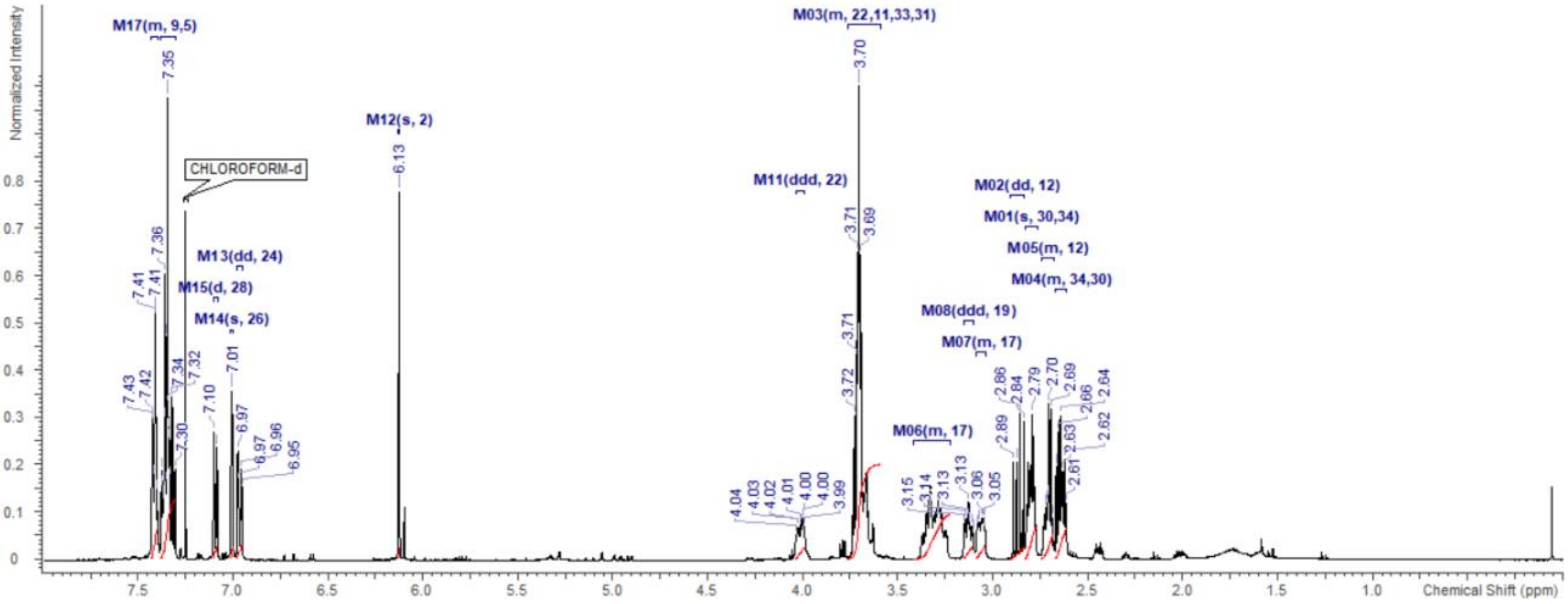

3-Morpholino-1-(2-oxo-1-phenyl-2-(4-(3-(trifluoromethyl)phenyl)piperazin-1-yl)ethyl)pyrrolidine-2,5-dione (16)

^{13}C NMR

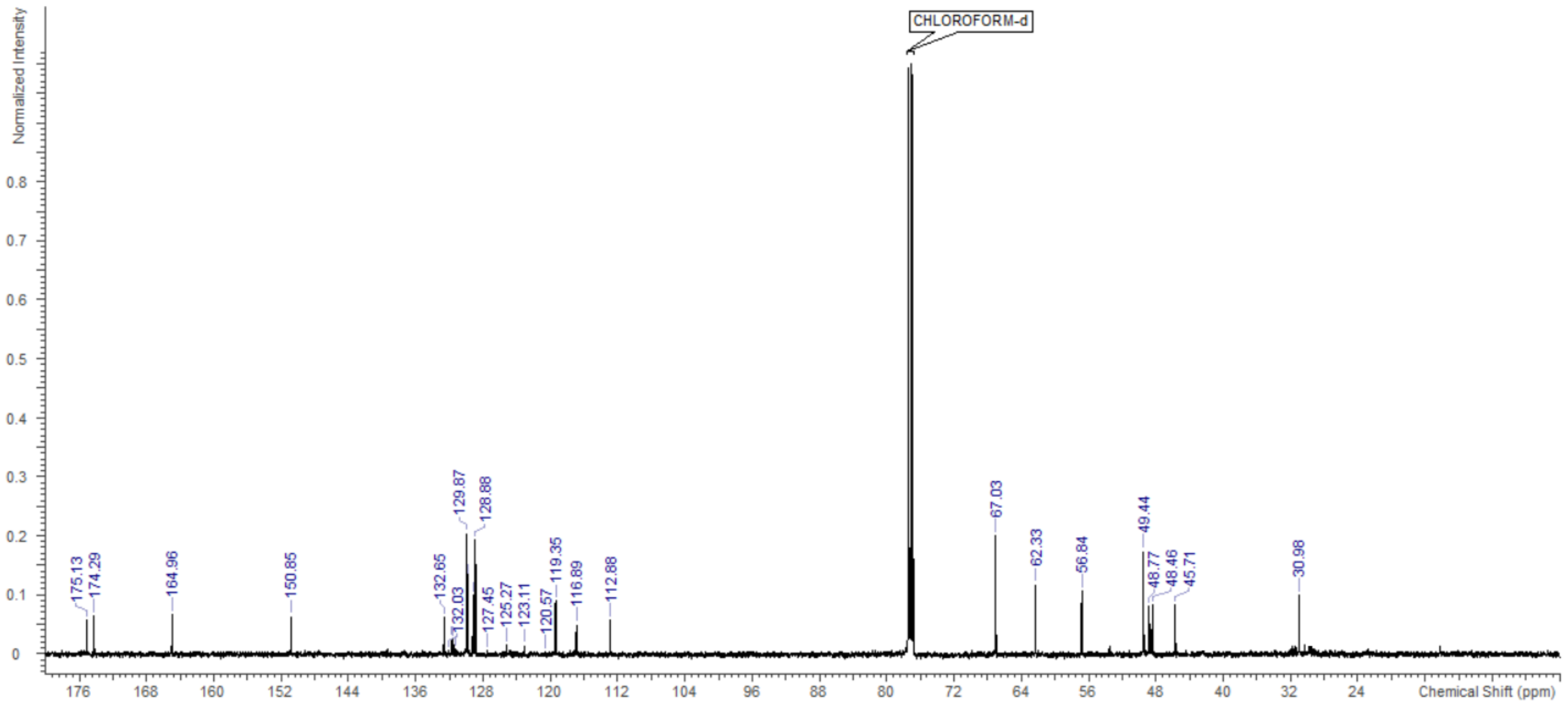

3-Dimethylamino-1-(2-oxo-1-phenyl-2-(4-(3-(trifluoromethoxy)phenyl)piperazin-1-yl)ethyl)pyrrolidine-2,5-dione (17)

^{1}H NMR

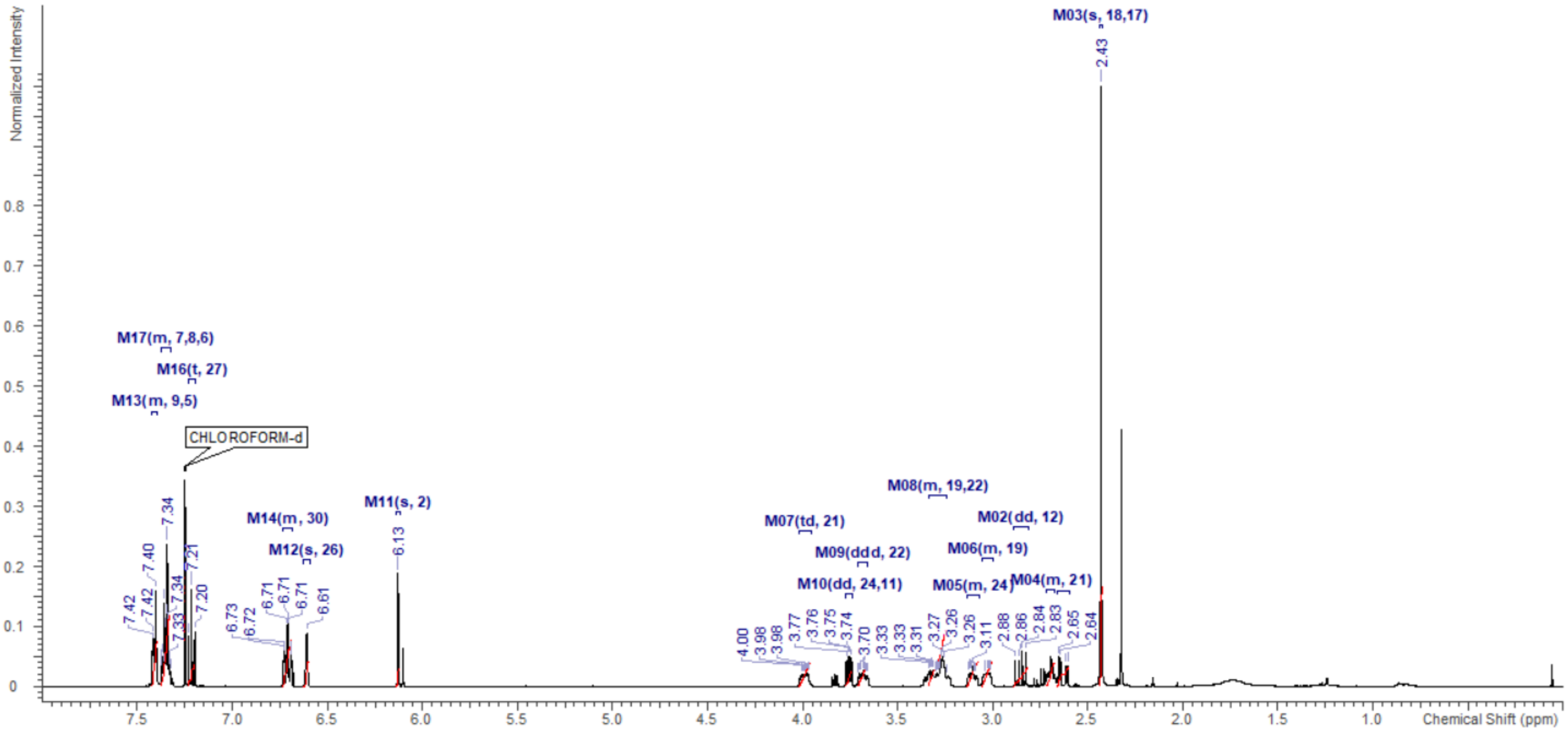

3-Dimethylamino-1-(2-oxo-1-phenyl-2-(4-(3-(trifluoromethoxy)phenyl)piperazin-1-yl)ethyl)pyrrolidine-2,5-dione (17)

^{13}C NMR

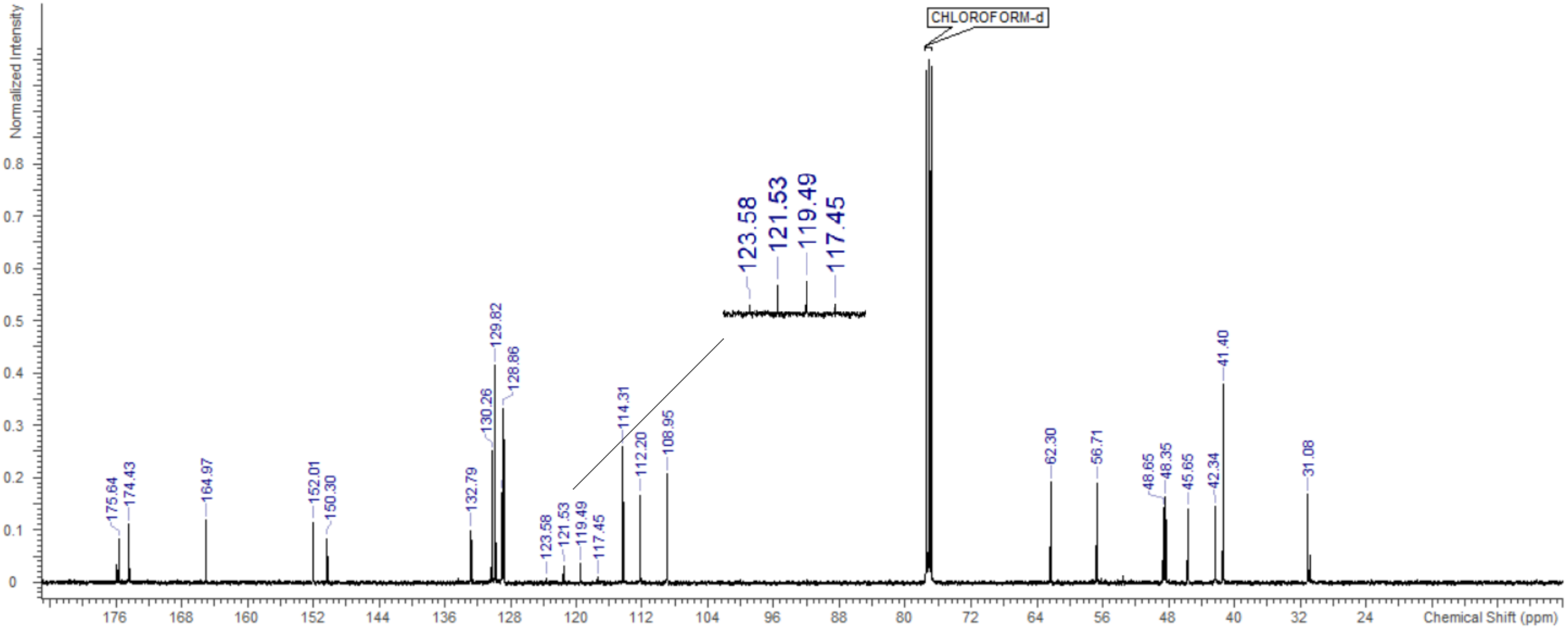

3-Dimethylamino-1-(2-oxo-1-phenyl-2-(4-(3-((trifluoromethyl)thio)phenyl)piperazin-1-yl)ethyl)pyrrolidine-2,5-dione (18)

^{1}H NMR

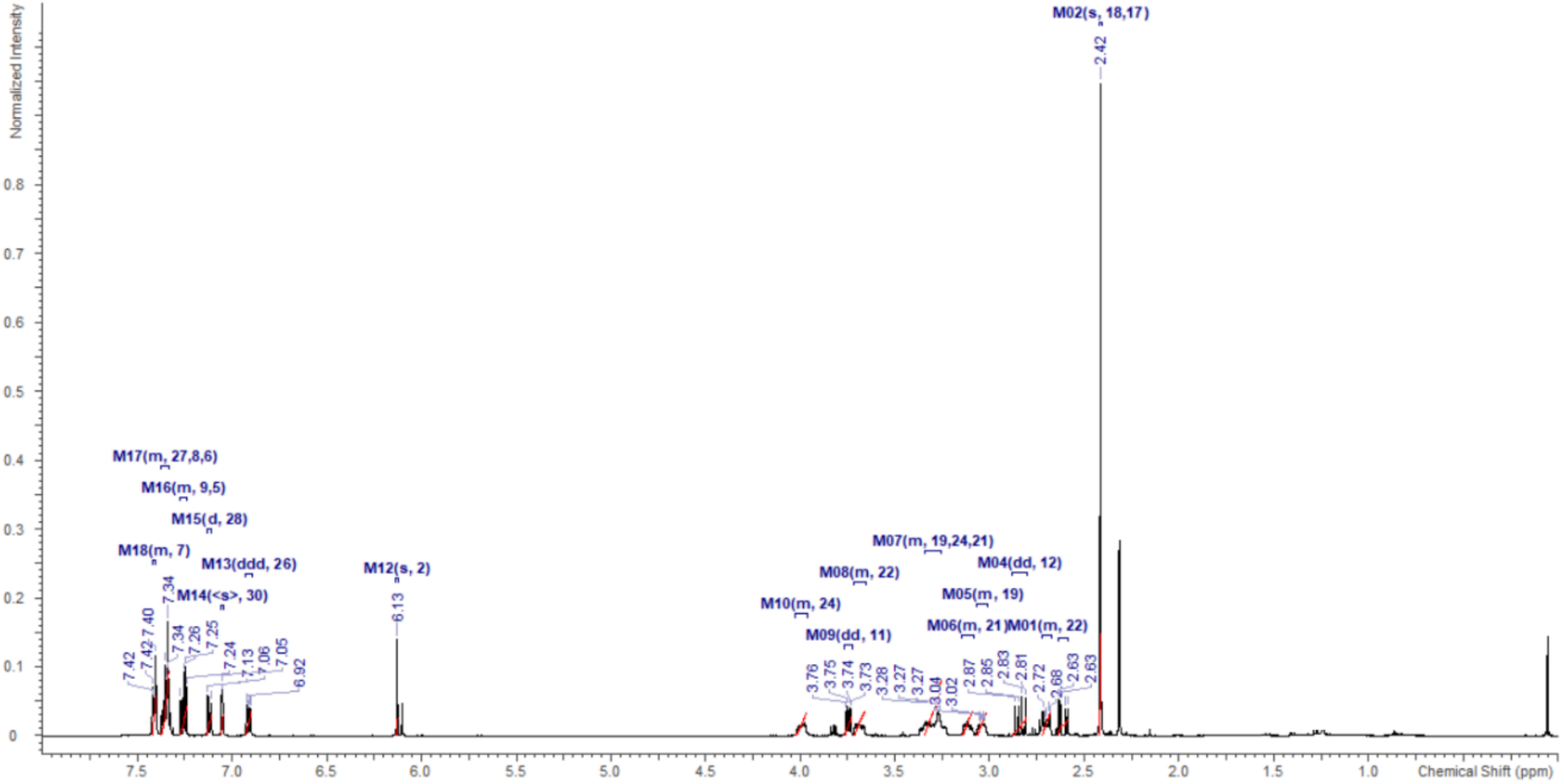

3-Dimethylamino-1-(2-oxo-1-phenyl-2-(4-(3-((trifluoromethyl)thio)phenyl)piperazin-1-yl)ethyl)pyrrolidine-2,5-dione (18)

^{13}C NMR

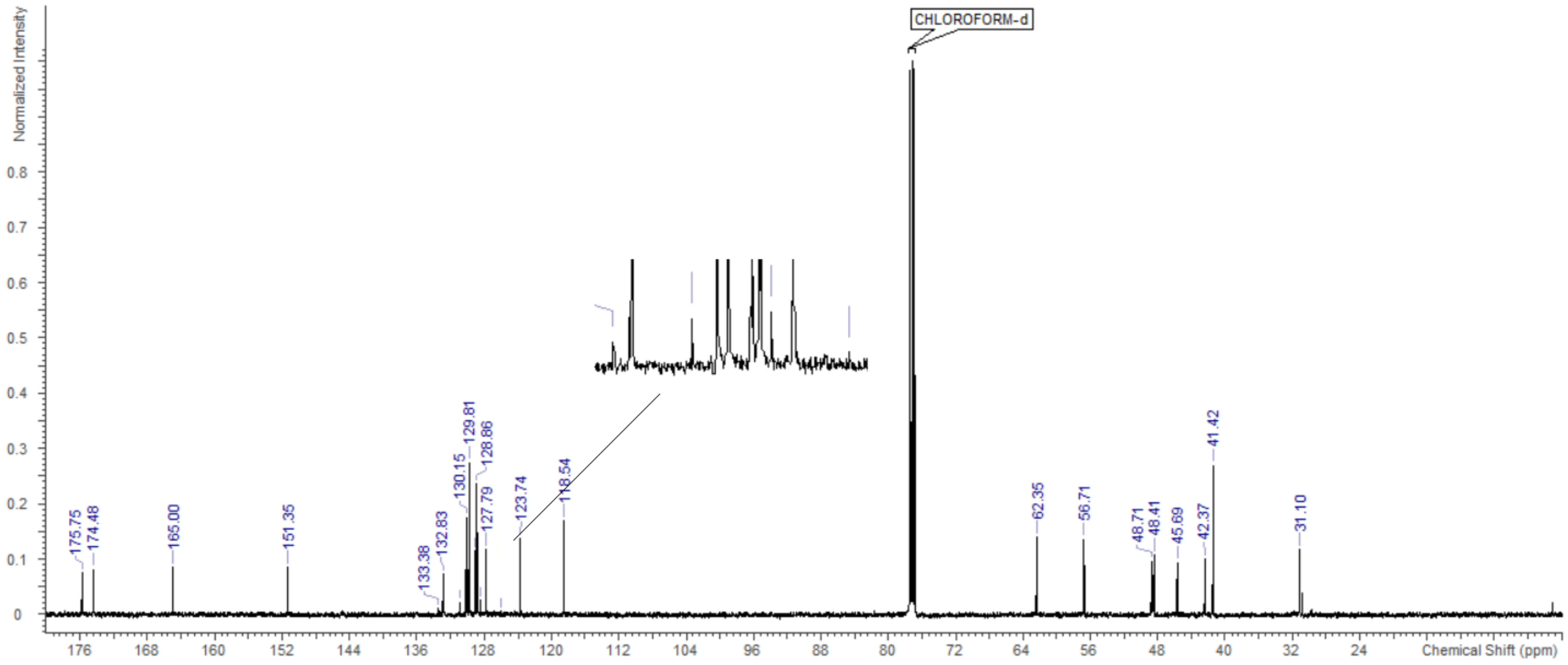

3-Dimethylamino-1-((*R*)-oxo-1-phenyl-2-(4-(3-(trifluoromethyl)phenyl)-piperazin-1-yl)ethyl)pyrrolidine-2,5-dione (C1-*R*)-31

^{1}H NMR

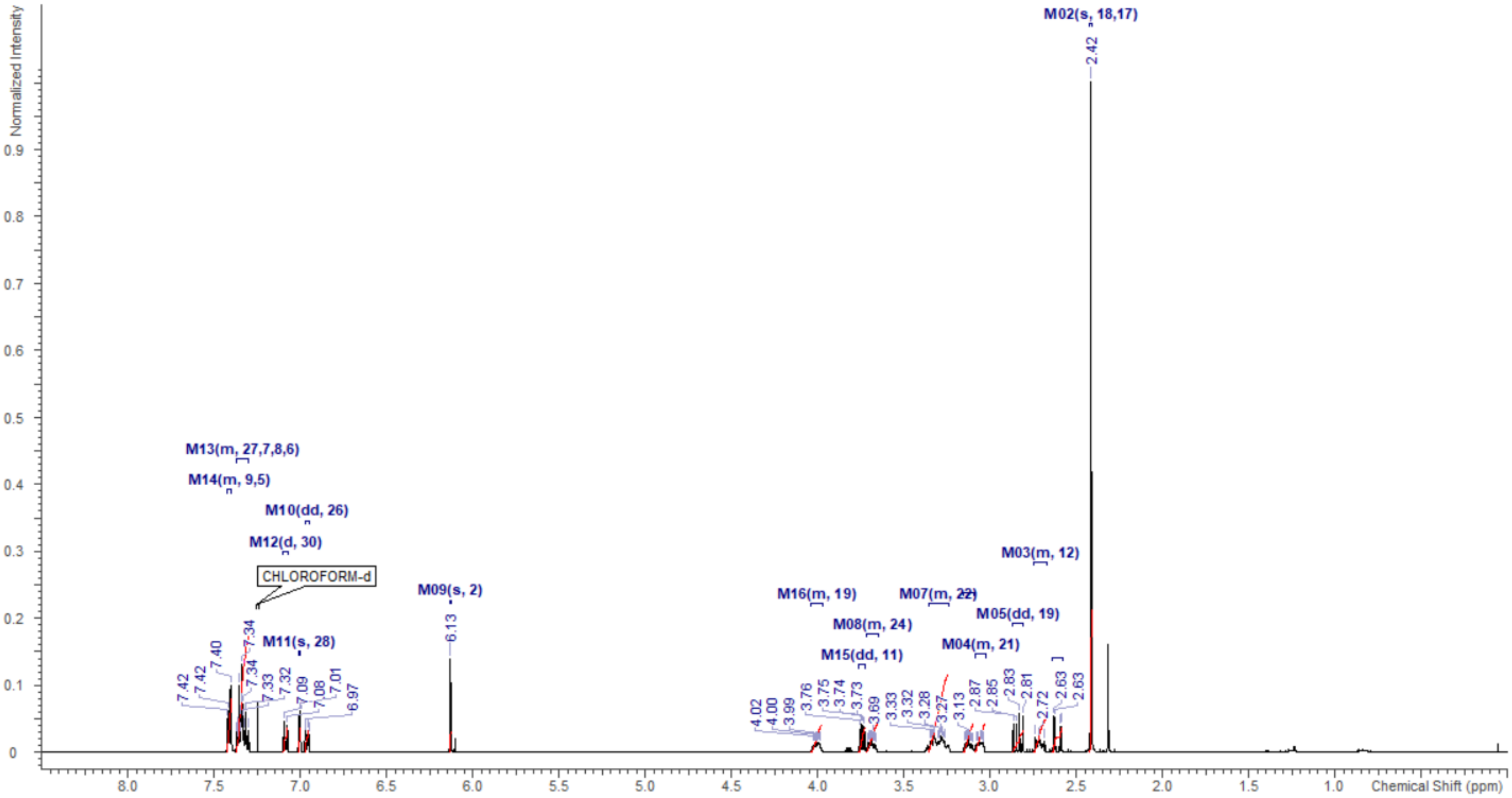

3-Dimethylamino-1-((*R*)-oxo-1-phenyl-2-(4-(3-(trifluoromethyl)phenyl)-piperazin-1-yl)ethyl)pyrrolidine-2,5-dione (C1-*R*)-31

^{13}C NMR

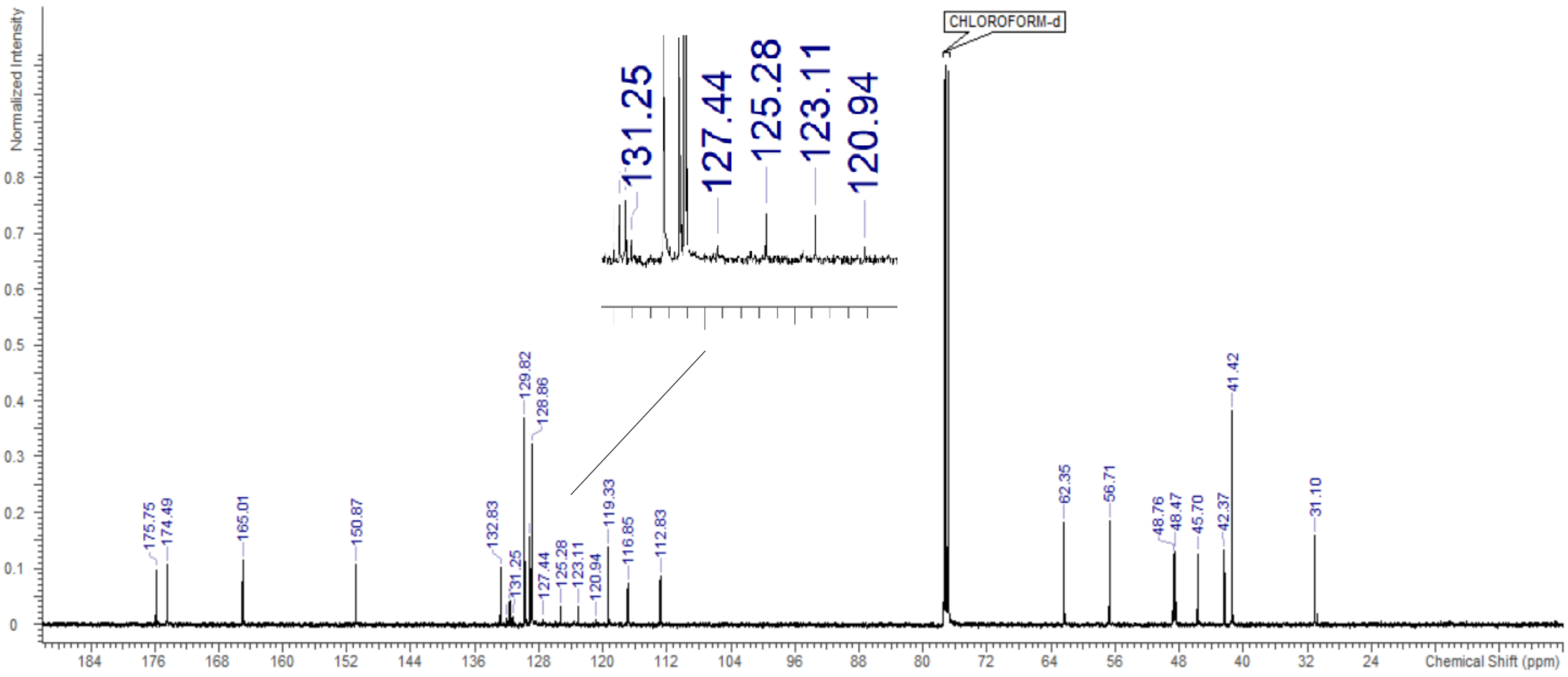

3-Dimethylamino-1-((*S*)-oxo-1-phenyl-2-(4-(3-(trifluoromethyl)phenyl)-piperazin-1-yl)ethyl)pyrrolidine-2,5-dione (C1-*S*)-31

^{1}H NMR

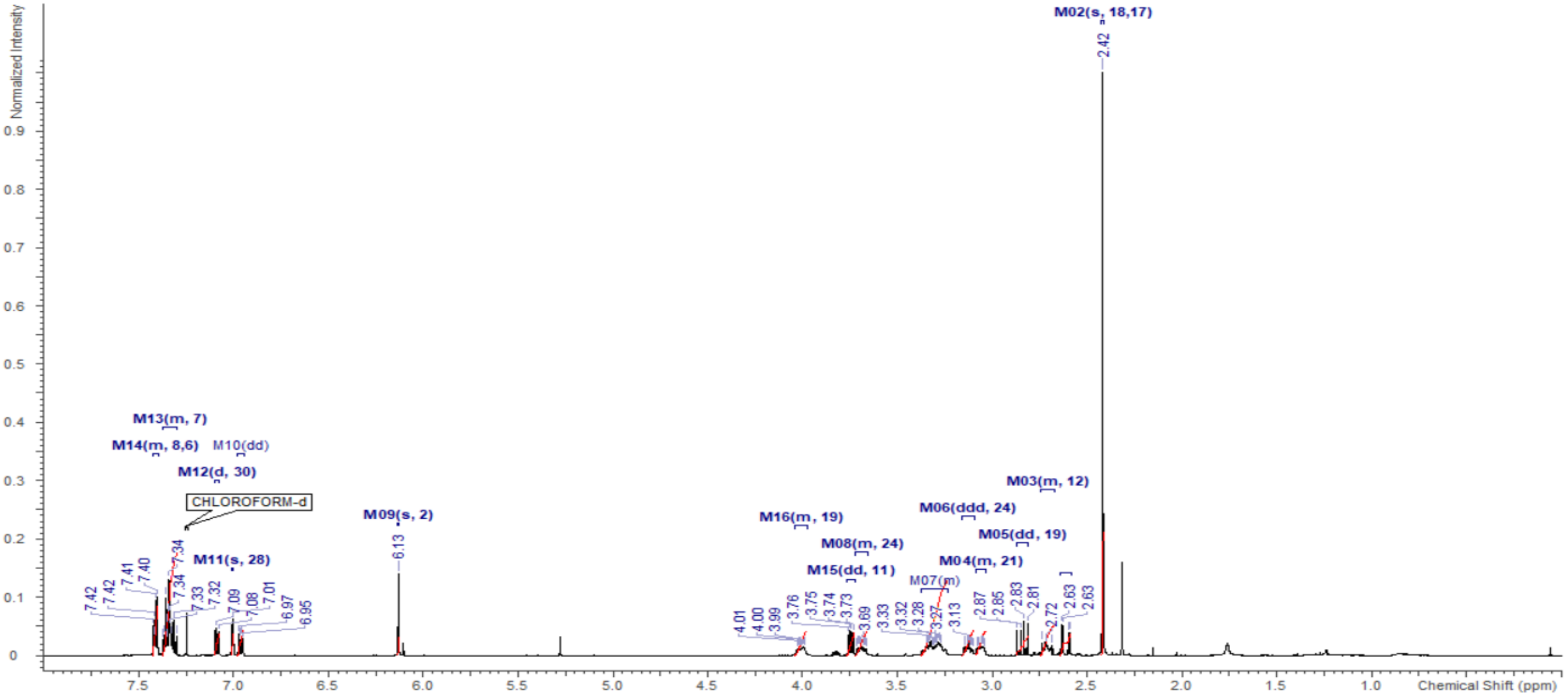

3-Dimethylamino-1-((*S*)-oxo-1-phenyl-2-(4-(3-(trifluoromethyl)phenyl)-piperazin-1-yl)ethyl)pyrrolidine-2,5-dione (C1-*S*)-31

^{13}C NMR

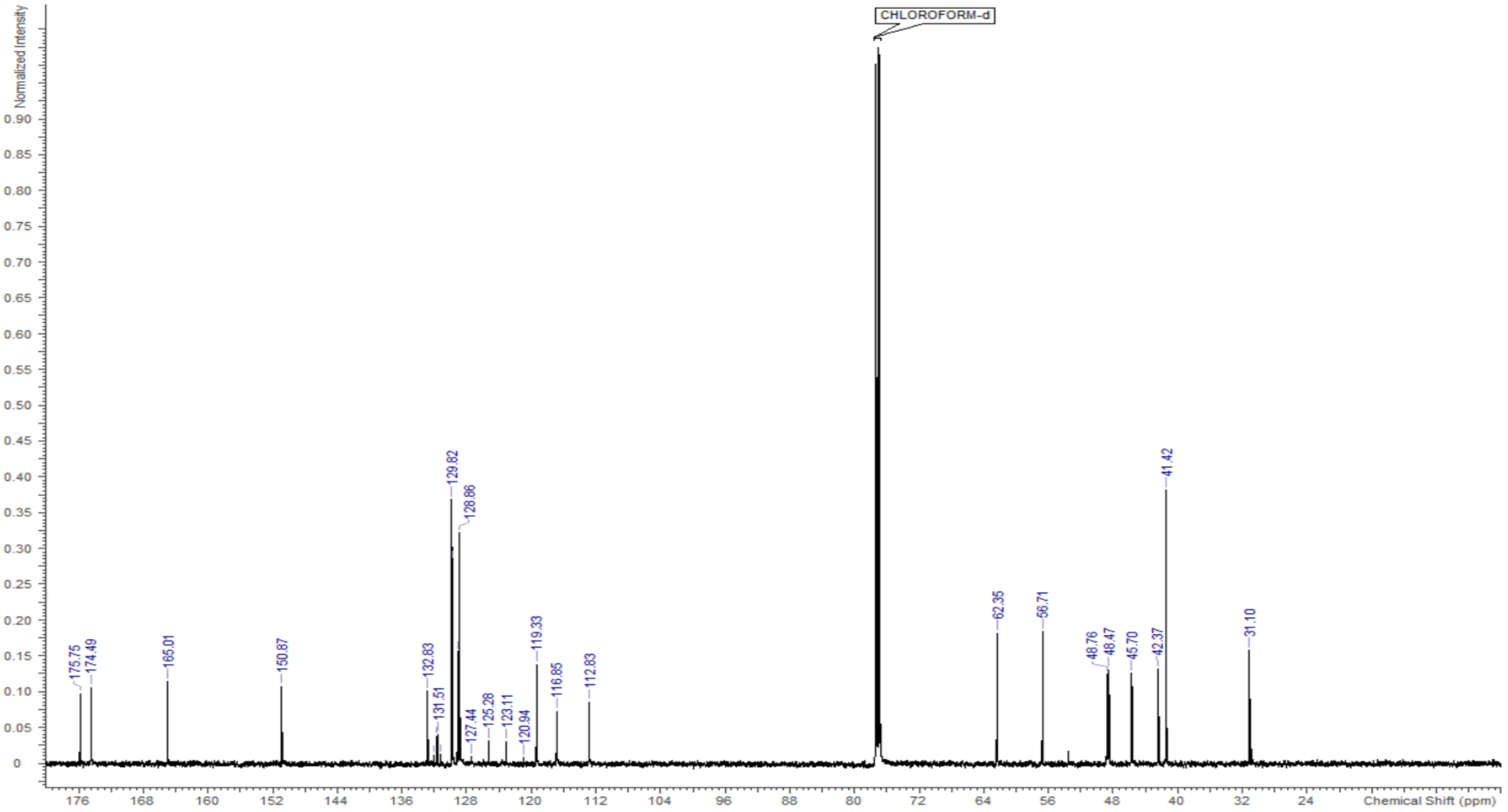

3-Dimethylamino-1-((*R*)-2-oxo-1-phenyl-2-(4-(3-(trifluoromethoxy)phenyl)piperazin-1-yl)ethyl)pyrrolidine-2,5-dione (C1-*R*)-32

^{1}H NMR

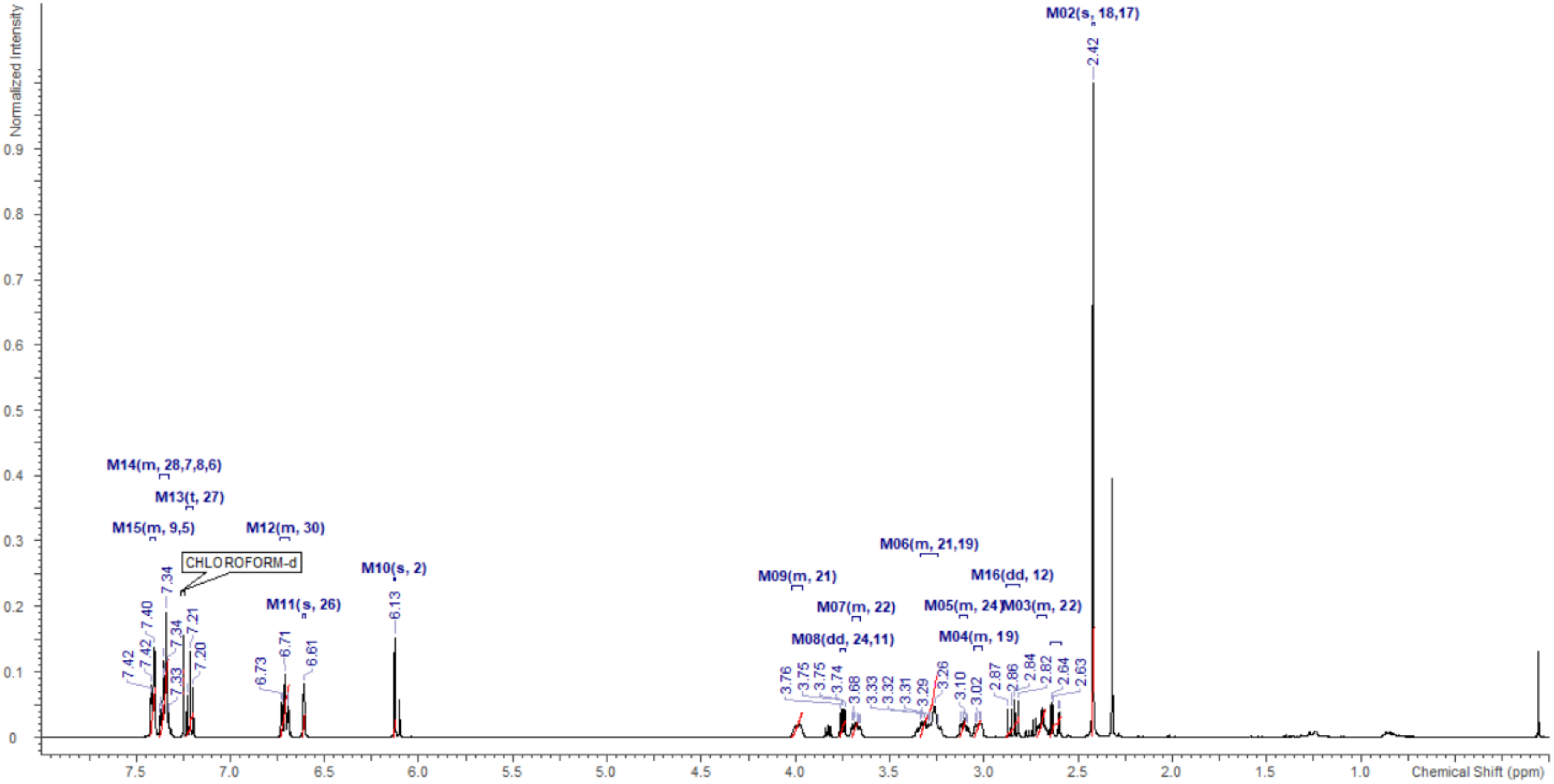

3-Dimethylamino-1-((*R*)-2-oxo-1-phenyl-2-(4-(3-(trifluoromethoxy)phenyl)piperazin-1-yl)ethyl)pyrrolidine-2,5-dione (C1-*R*)-32

^{13}C NMR

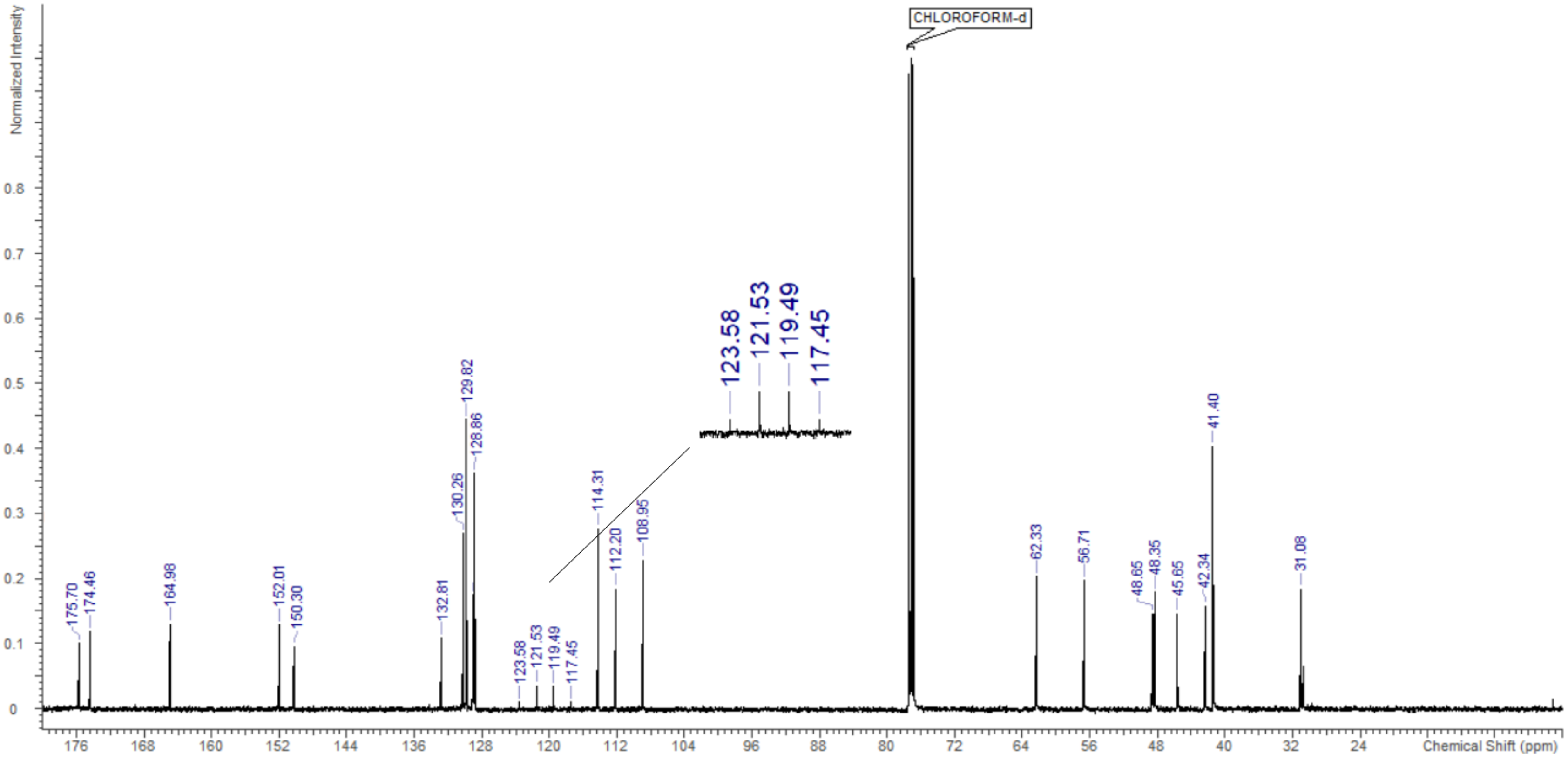

3-Dimethylamino-1-((*R*)-2-oxo-1-phenyl-2-(4-(3-((trifluoromethyl)thio)phenyl)piperazin-1-yl)ethyl)pyrrolidine-2,5-dione (C1-*R*)-33

^{1}H NMR

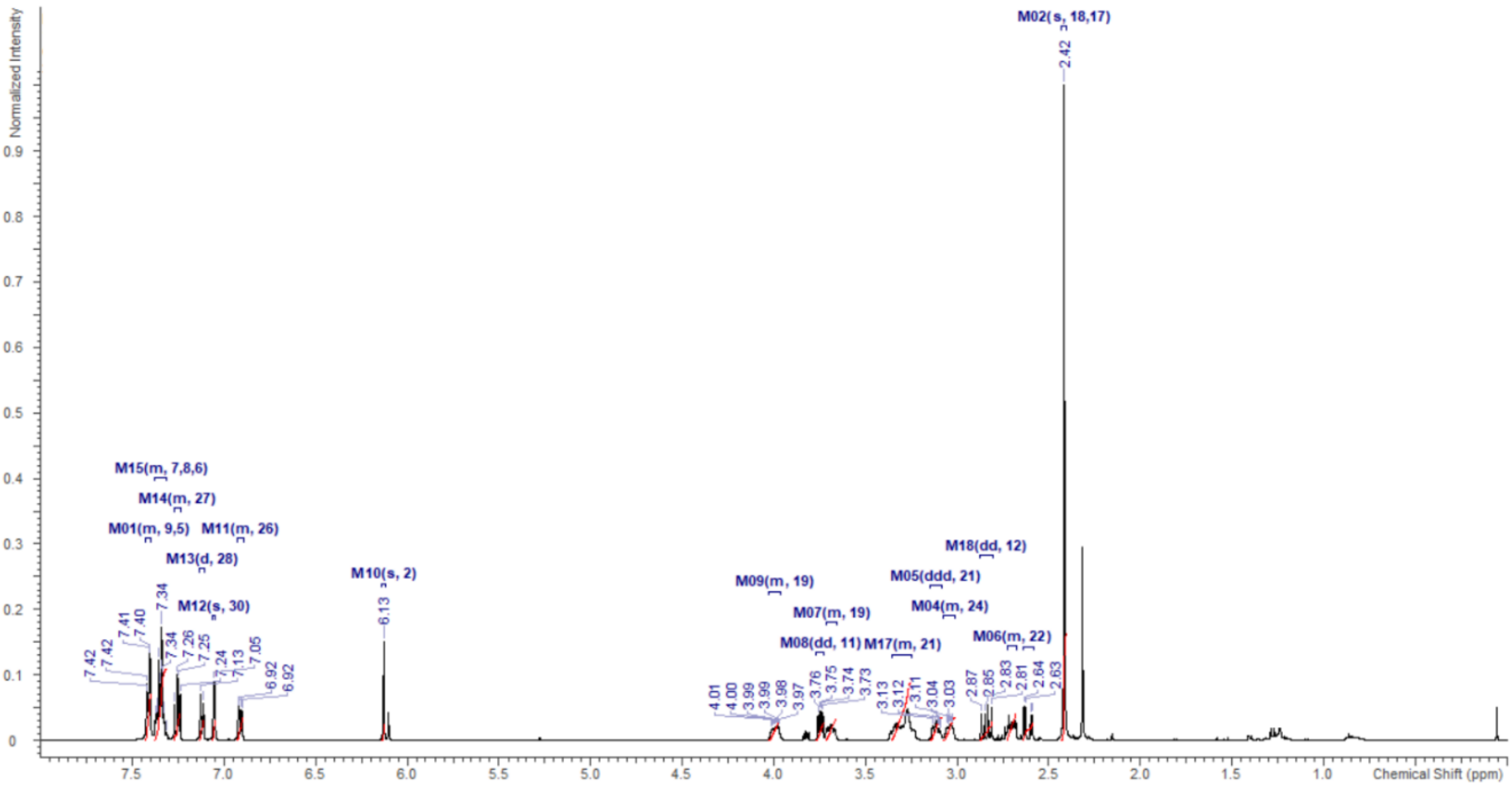

3-Dimethylamino-1-((*R*)-2-oxo-1-phenyl-2-(4-(3-((trifluoromethyl)thio)phenyl)piperazin-1-yl)ethyl)pyrrolidine-2,5-dione (C1-*R*)-33

^{13}C NMR

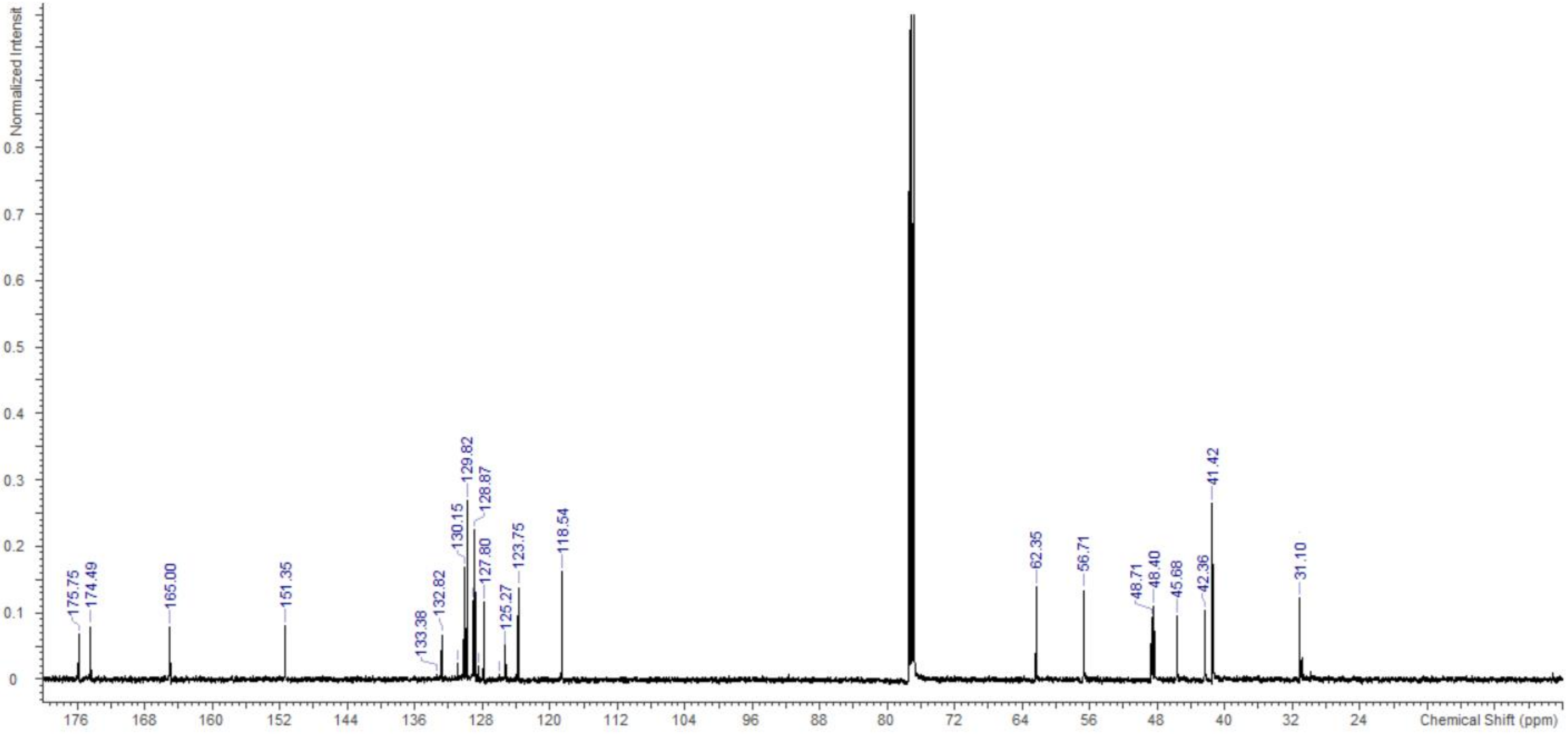

Chiral HPLC chromatograms

(*R,S*)-1-(2-Oxo-1-phenyl-2-(4-(3-(trifluoromethyl)phenyl)piperazin-1-yl)ethyl)-1*H*-pyrrole-2,5-dione (10)

15.10.2021 16:53:09 Page 1 / 1

Analysis Report

SHIMADZU LabSolutions

<Sample Information>

Sample Name	: R,S-KA-214		
Sample ID	:		
Data Filename	: R,S-KA-214.lcd		
Method Filename	: chiralneKA.lcm		
Batch Filename	:		
Vial #	: 1-26	Sample Type	: Unknown
Injection Volume	: 10 uL		
Date Acquired	: 04.10.2021 15:55:28	Acquired by	: Chemia leków1
Date Processed	: 08.10.2021 15:22:45	Processed by	: Chemia leków1

<Chromatogram>

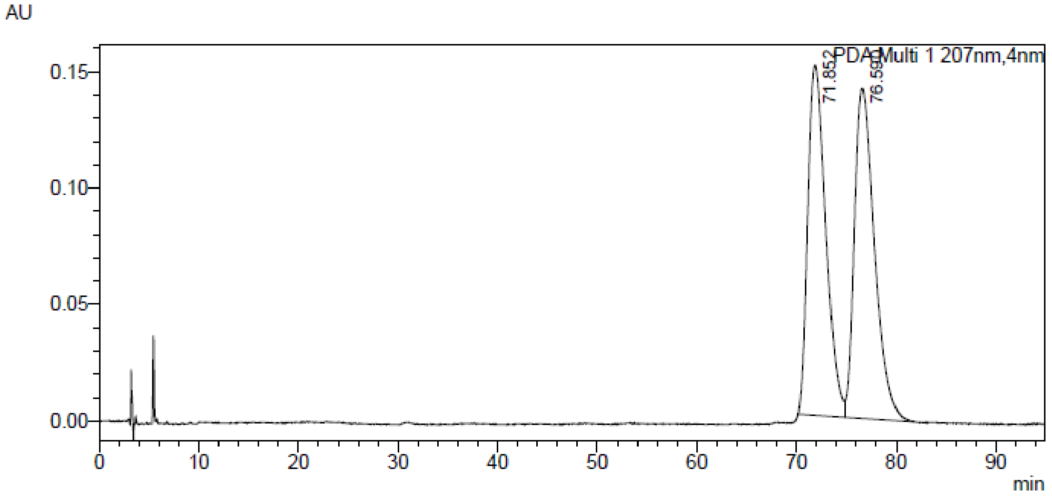

<Peak Table>

PDA Ch1 207nm

Peak#	Ret. Time	Area%
1	71.852	49.001
2	76.590	50.999
Total		100.000

(*R*)-1-(2-Oxo-1-phenyl-2-(4-(3-(trifluoromethyl)phenyl)piperazin-1-yl)ethyl)-1*H*-pyrrole-2,5-dione (C1-*R*)-28

15.10.2021 16:38:30 Page 1 / 1

SHIMADZU LabSolutions

Analysis Report

<Sample Information>

Sample Name	: R-KA-214		
Sample ID	:		
Data Filename	: R-KA-214.lcd		
Method Filename	: chiralneKA.lcm		
Batch Filename	:		
Vial #	: 1-4	Sample Type	: Unknown
Injection Volume	: 10 uL		
Date Acquired	: 08.10.2021 09:18:37	Acquired by	: Chemia leków1
Date Processed	: 15.10.2021 16:32:07	Processed by	: Chemia leków1

<Chromatogram>

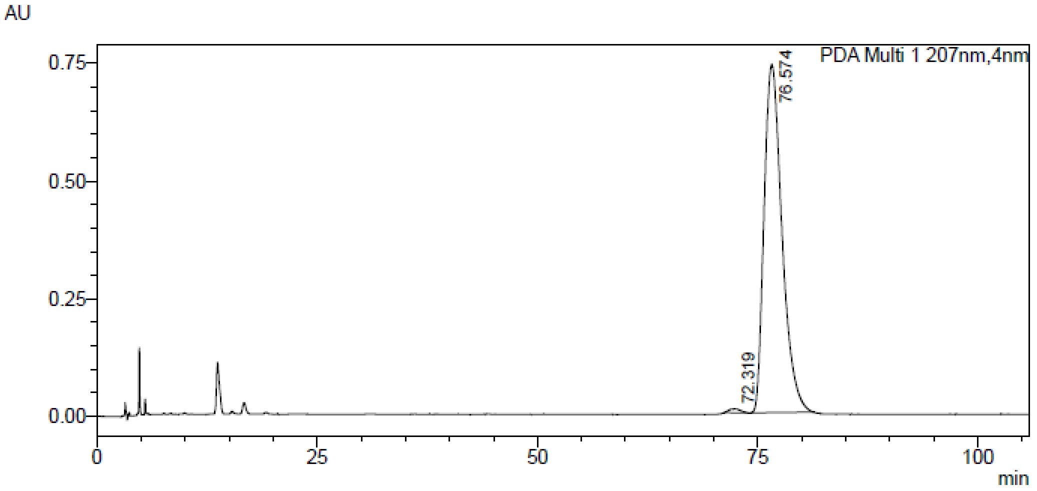

<Peak Table>

PDA Ch1 207nm

Peak#	Ret. Time	Area%
1	72.319	0.706
2	76.574	99.294
Total		100.000

(*S*)-1-(2-Oxo-1-phenyl-2-(4-(3-(trifluoromethyl)phenyl)piperazin-1-yl)ethyl)-1*H*-pyrrole-2,5-dione (C1-*S*)-28

15.10.2021 16:36:43 Page 1 / 1

SHIMADZU LabSolutions **Analysis Report**

<Sample Information>

Sample Name	: S-KA-214		
Sample ID	:		
Data Filename	: S-KA-214.lcd		
Method Filename	: chiralneKA.lcm		
Batch Filename	:		
Vial #	: 1-6	Sample Type	: Unknown
Injection Volume	: 10 uL		
Date Acquired	: 08.10.2021 17:15:24	Acquired by	: Chemia leków1
Date Processed	: 11.10.2021 10:17:25	Processed by	: Chemia leków1

<Chromatogram>

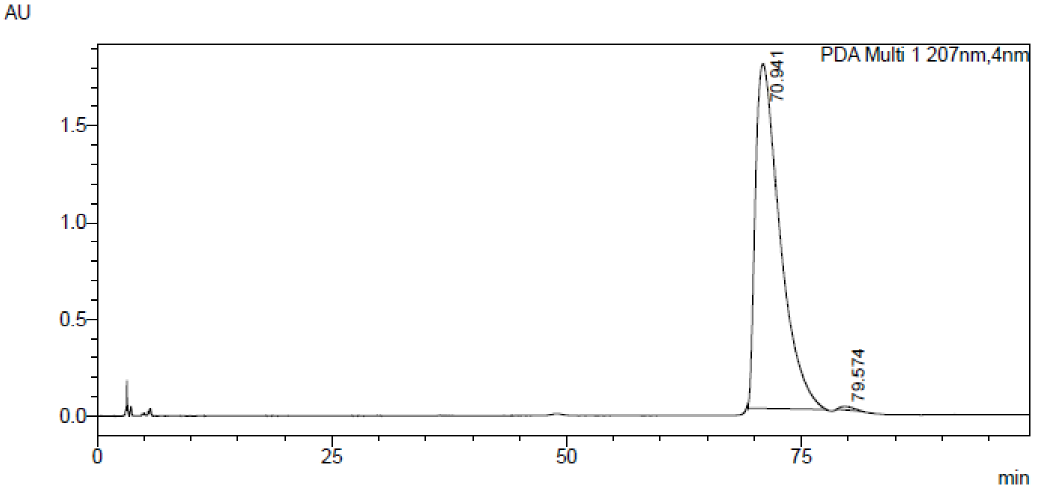

<Peak Table>

PDA Ch1 207nm

Peak#	Ret. Time	Area%
1	70.941	99.454
2	79.574	0.546
Total		100.000

(*R,S*)-1-(2-Oxo-1-phenyl-2-(4-(3-(trifluoromethoxy)phenyl)piperazin-1-yl)ethyl)-1*H*-pyrrole-2,5-dione (11)

15.10.2021 16:50:09 Page 1 / 1

SHIMADZU LabSolutions

Analysis Report

<Sample Information>

Sample Name : R,S-KA-236
Sample ID :
Data Filename : R,S-KA-236.lcd
Method Filename : chiralneKA.lcm
Batch Filename :
Vial # : 1-25
Sample Type : Unknown
Injection Volume : 10 uL
Date Acquired : 04.10.2021 14:28:47
Acquired by : Chemia leków1
Date Processed : 04.10.2021 15:53:46
Processed by : Chemia leków1

<Chromatogram>

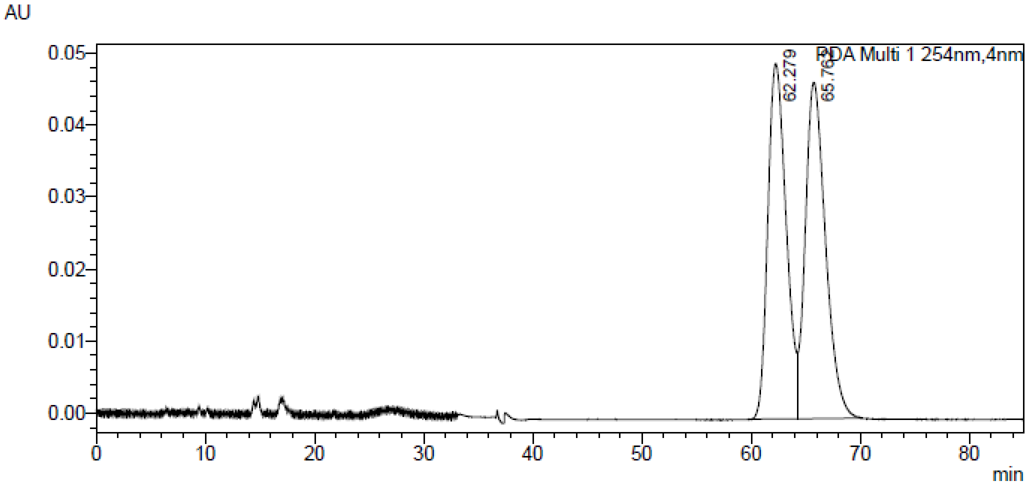

<Peak Table>

PDA Ch1 254nm

Peak#	Ret. Time	Area%
1	62.279	48.835
2	65.762	51.165
Total		100.000

(*R*)-1-(2-Oxo-1-phenyl-2-(4-(3-(trifluoromethoxy)phenyl)piperazin-1-yl)ethyl)-1*H*-pyrrole-2,5-dione (C1-*R*)-29

15.10.2021 16:46:26 Page 1 / 1

SHIMADZU LabSolutions **Analysis Report**

<Sample Information>

Sample Name : R-KA-236
Sample ID :
Data Filename : R-KA-236.lcd
Method Filename : chiralneKA.lcm
Batch Filename :
Vial # : 1-5
Sample Type : Unknown
Injection Volume : 10 uL
Date Acquired : 08.10.2021 11:23:18
Acquired by : Chemia leków1
Date Processed : 08.10.2021 15:26:40
Processed by : Chemia leków1

<Chromatogram>

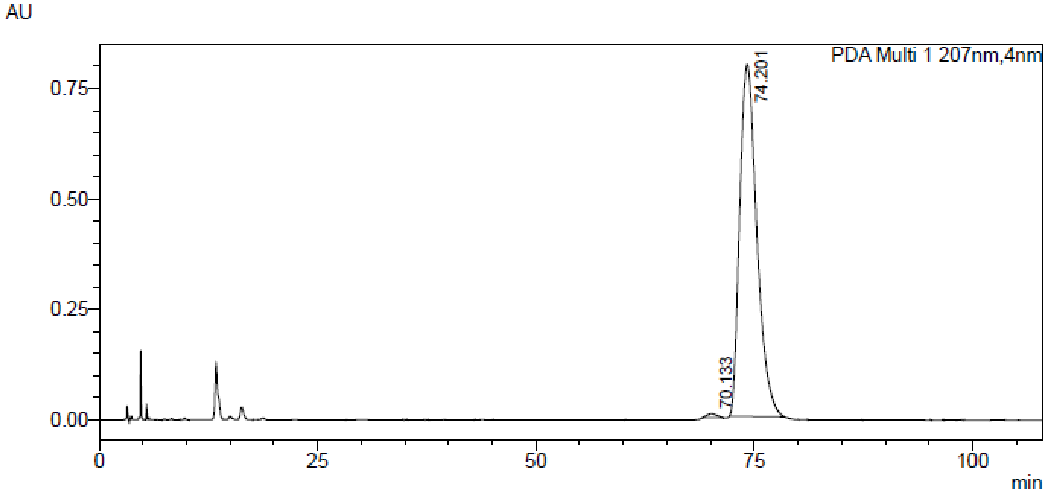

<Peak Table>

PDA Ch1 207nm

Peak#	Ret. Time	Area%
1	70.133	0.663
2	74.201	99.337
Total		100.000

(*R,S*)-1-(2-Oxo-1-phenyl-2-(4-(3-((trifluoromethyl)thio)phenyl)piperazin-1-yl)ethyl)-1*H*-pyrrole-2,5-dione (12)

15.10.2021 16:25:54 Page 1 / 1

SHIMADZU LabSolutions **Analysis Report**

<Sample Information>

Sample Name	: R,S-KA-235
Sample ID	:
Data Filename	: R,S-KA-235.lcd
Method Filename	: chiralneKA.lcm
Batch Filename	:
Vial #	: 1-2
Injection Volume	: 10 uL
Date Acquired	: 07.10.2021 11:26:15
Date Processed	: 08.10.2021 15:29:24
Sample Type	: Unknown
Acquired by	: Chemia leków1
Processed by	: Chemia leków1

<Chromatogram>

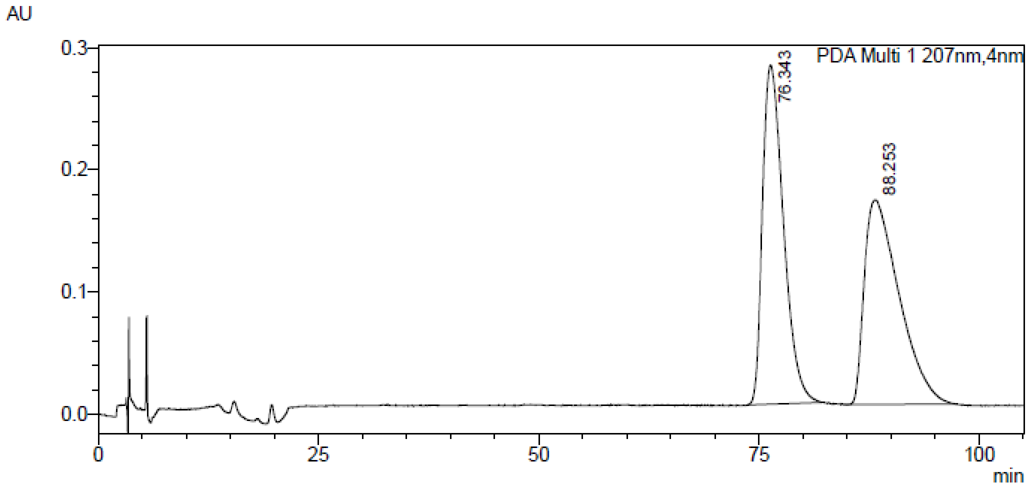

<Peak Table>

PDA Ch1 207nm

Peak#	Ret. Time	Area%
1	76.343	49.847
2	88.253	50.153
Total		100.000

(*R*)-1-(2-Oxo-1-phenyl-2-(4-(3-((trifluoromethyl)thio)phenyl)piperazin-1-yl)ethyl)-1*H*-pyrrole-2,5-dione (C1-*R*)-30

15.10.2021 16:39:50 Page 1 / 1

SHIMADZU LabSolutions

Analysis Report

<Sample Information>

Sample Name	: R-KA-235		
Sample ID	:		
Data Filename	: R-KA-235.lcd		
Method Filename	: chiralneKA.lcm		
Batch Filename	:		
Vial #	: 1-3	Sample Type	: Unknown
Injection Volume	: 10 uL		
Date Acquired	: 07.10.2021 13:12:21	Acquired by	: Chemia leków1
Date Processed	: 15.10.2021 16:28:20	Processed by	: Chemia leków1

<Chromatogram>

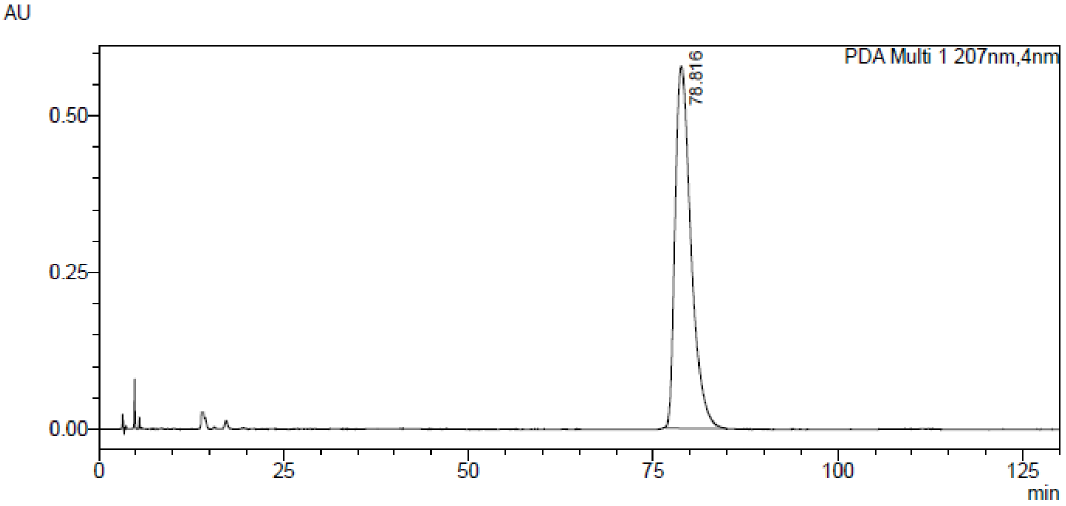

<Peak Table>

PDA Ch1 207nm

Peak#	Ret. Time	Area%
1	78.816	100.000
Total		100.000

3-(Dimethylamino)-1-(2-oxo-1-phenyl-2-(4-(3-(trifluoromethyl)phenyl)-piperazin-1-yl)ethyl)pyrrolidine-2,5-dione (14)

15.10.2021 15:31:33 Page 1 / 1

SHIMADZU LabSolutions

Analysis Report

<Sample Information>

Sample Name	: R,S-KA-223		
Sample ID	:		
Data Filename	: R,S-KA-223.lcd		
Method Filename	: chiralneKA.lcm		
Batch Filename	:		
Vial #	: 1-16	Sample Type	: Unknown
Injection Volume	: 10 uL		
Date Acquired	: 15.10.2021 12:59:22	Acquired by	: Chemia leków1
Date Processed	: 15.10.2021 14:09:28	Processed by	: Chemia leków1

<Chromatogram>

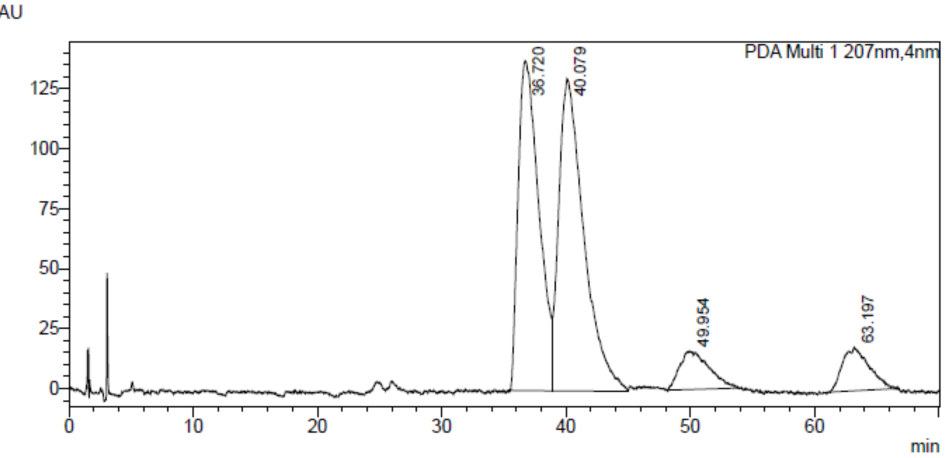

<Peak Table>

PDA Ch1 207nm

Peak#	Ret. Time	Area%
1	36.720	40.179
2	40.079	46.243
3	49.954	6.694
4	63.197	6.884
Total		100.000

3-Dimethylamino-1-((*R*)-oxo-1-phenyl-2-(4-(3-(trifluoromethyl)phenyl)-piperazin-1-yl)ethyl)pyrrolidine-2,5-dione (C1-*R*)-31

15.10.2021 16:02:41 Page 1 / 1

SHIMADZU LabSolutions

Analysis Report

<Sample Information>

Sample Name	: R-KA-223		
Sample ID	:		
Data Filename	: R-KA-223.lcd		
Method Filename	: chiralneKA.lcm		
Batch Filename	:		
Vial #	: 1-10	Sample Type	: Unknown
Injection Volume	: 10 uL		
Date Acquired	: 14.10.2021 09:14:45	Acquired by	: Chemia leków1
Date Processed	: 14.10.2021 10:26:06	Processed by	: Chemia leków1

<Chromatogram>

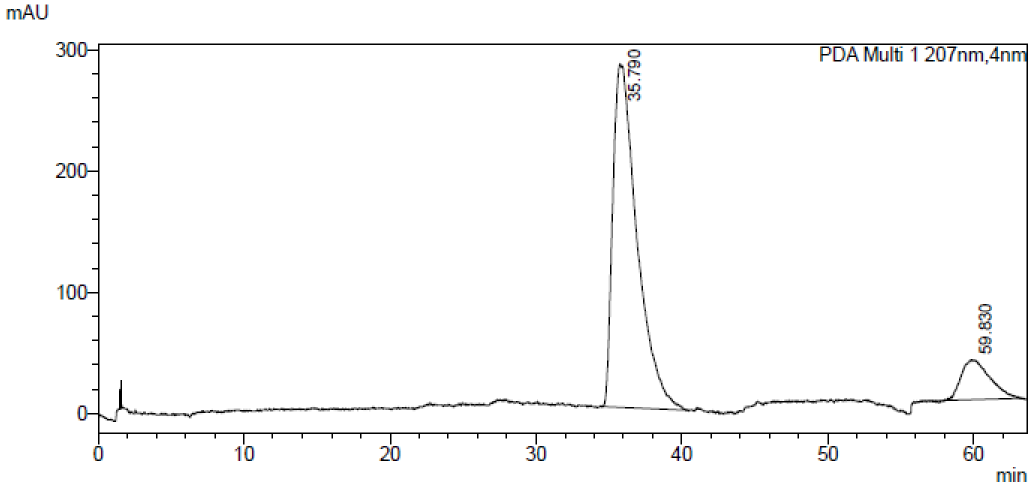

<Peak Table>

PDA Ch1 207nm

Peak#	Ret. Time	Area%
1	35.790	87.957
2	59.830	12.043
Total		100.000

3-Dimethylamino-1-((*S*)-oxo-1-phenyl-2-(4-(3-(trifluoromethyl)phenyl)-piperazin-1-yl)ethyl)pyrrolidine-2,5-dione (C1-*S*)-31

SHIMADZU LabSolutions

Analysis Report

<Sample Information>

Sample Name	: S-KA-223		
Sample ID	:		
Data Filename	: S-KA-223.lcd		
Method Filename	: chiralneKA.lcm		
Batch Filename	:		
Vial #	: 1-11	Sample Type	: Unknown
Injection Volume	: 10 uL		
Date Acquired	: 14.10.2021 10:19:12	Acquired by	: Chemia leków1
Date Processed	: 14.10.2021 11:33:45	Processed by	: Chemia leków1

<Chromatogram>

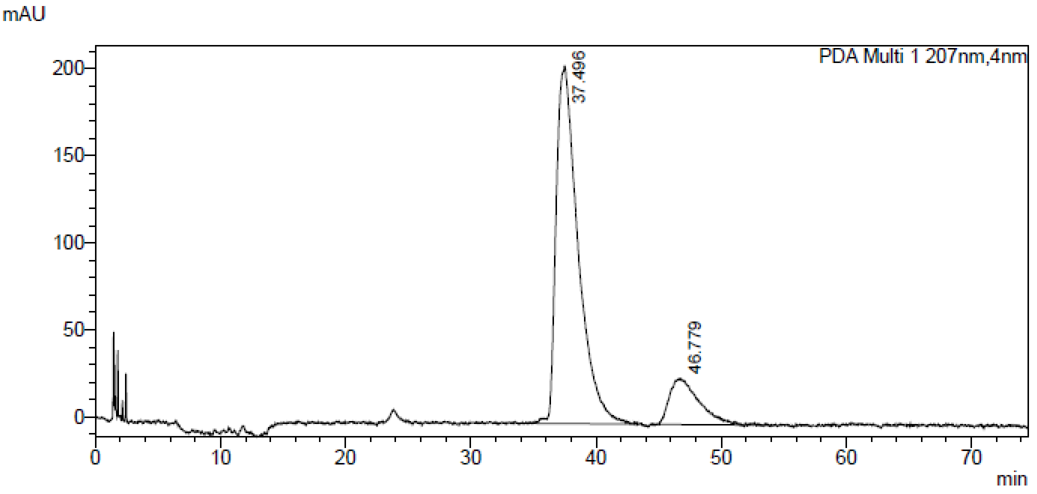

<Peak Table>

PDA Ch1 207nm

Peak#	Ret. Time	Area%
1	37.496	85.241
2	46.779	14.759
Total		100.000

3-Dimethylamino-1-(2-oxo-1-phenyl-2-(4-(3-(trifluoromethoxy)phenyl)piperazin-1-yl)ethyl)pyrrolidine-2,5-dione (17)

15.10.2021 16:19:46 Page 1 / 1

SHIMADZU LabSolutions

Analysis Report

<Sample Information>

Sample Name : R,S-KA-238
Sample ID :
Data Filename : R,S-KA-238.lcd
Method Filename : chiralneKA.lcm
Batch Filename :
Vial # : 1-7
Sample Type : Unknown
Injection Volume : 10 uL
Date Acquired : 13.10.2021 14:07:11
Acquired by : Chemia leków1
Date Processed : 13.10.2021 15:21:00
Processed by : Chemia leków1

<Chromatogram>

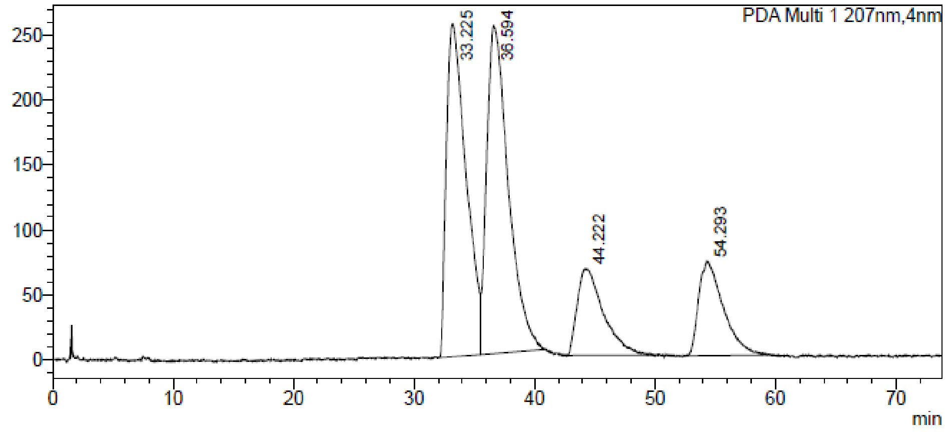

<Peak Table>

PDA Ch1 207nm

Peak#	Ret. Time	Area%
1	33.225	35.932
2	36.594	38.884
3	44.222	12.433
4	54.293	12.750
Total		100.000

3-Dimethylamino-1-((*R*)-2-oxo-1-phenyl-2-(4-(3-(trifluoromethoxy)phenyl)piperazin-1-yl)ethyl)pyrrolidine-2,5-dione (C1-*R*)-32

15.10.2021 16:05:39 Page 1 / 1

SHIMADZU LabSolutions **Analysis Report**

<Sample Information>

Sample Name	: R-KA-238		
Sample ID	:		
Data Filename	: R-KA-238.lcd		
Method Filename	: chiralneKA.lcm		
Batch Filename	:		
Vial #	: 1-8	Sample Type	: Unknown
Injection Volume	: 10 uL		
Date Acquired	: 13.10.2021 15:22:24	Acquired by	: Chemia leków1
Date Processed	: 13.10.2021 16:22:17	Processed by	: Chemia leków1

<Chromatogram>

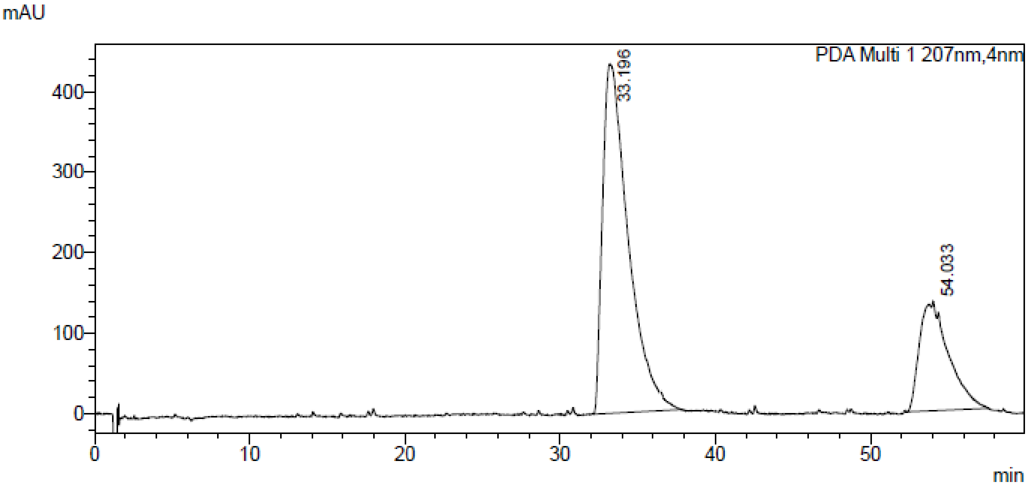

<Peak Table>

PDA Ch1 207nm

Peak#	Ret. Time	Area%
1	33.196	73.741
2	54.033	26.259
Total		100.000

3-Dimethylamino-1-(2-oxo-1-phenyl-2-(4-(3-((trifluoromethyl)thio)phenyl)piperazin-1-yl)ethyl)pyrrolidine-2,5-dione (18)

15.10.2021 15:49:19 Page 1 / 1

SHIMADZU LabSolutions **Analysis Report**

<Sample Information>

Sample Name	: R,S-KA-237		
Sample ID	:		
Data Filename	: R,S-KA-237.lcd		
Method Filename	: chiralneKA.lcm		
Batch Filename	:		
Vial #	: 1-14	Sample Type	: Unknown
Injection Volume	: 10 uL		
Date Acquired	: 15.10.2021 09:47:52	Acquired by	: Chemia leków1
Date Processed	: 15.10.2021 10:57:21	Processed by	: Chemia leków1

<Chromatogram>

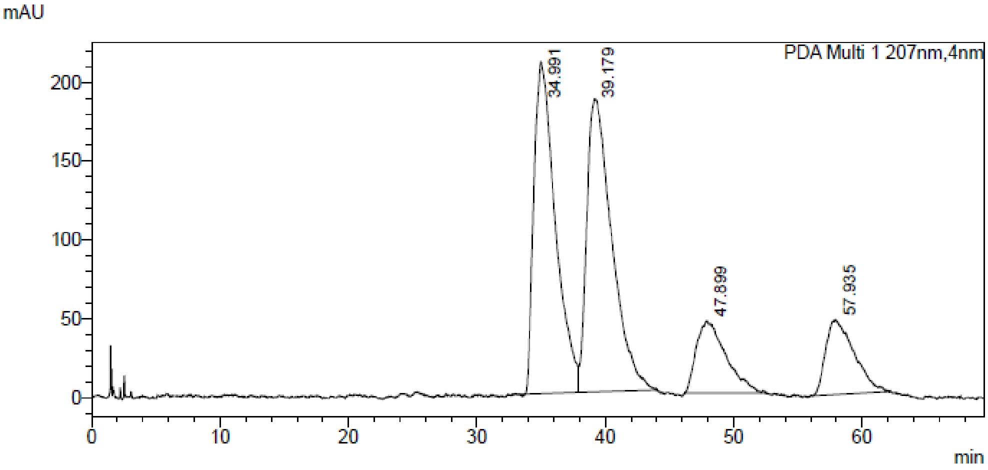

<Peak Table>

PDA Ch1 207nm

Peak#	Ret. Time	Area%
1	34.991	38.066
2	39.179	39.301
3	47.899	11.345
4	57.935	11.288
Total		100.000

3-Dimethylamino-1-((*R*)-2-oxo-1-phenyl-2-(4-(3-((trifluoromethyl)thio)phenyl)piperazin-1-yl)ethyl)pyrrolidine-2,5-dione (C1-*R*)-33

15.10.2021 15:38:50 Page 1 / 1

SHIMADZU LabSolutions **Analysis Report**

<Sample Information>

Sample Name	: R-KA-237		
Sample ID	:		
Data Filename	: R-KA-237.lcd		
Method Filename	: chiralneKA.lcm		
Batch Filename	:		
Vial #	: 1-15	Sample Type	: Unknown
Injection Volume	: 10 uL		
Date Acquired	: 15.10.2021 11:07:19	Acquired by	: Chemia leków1
Date Processed	: 15.10.2021 12:17:01	Processed by	: Chemia leków1

<Chromatogram>

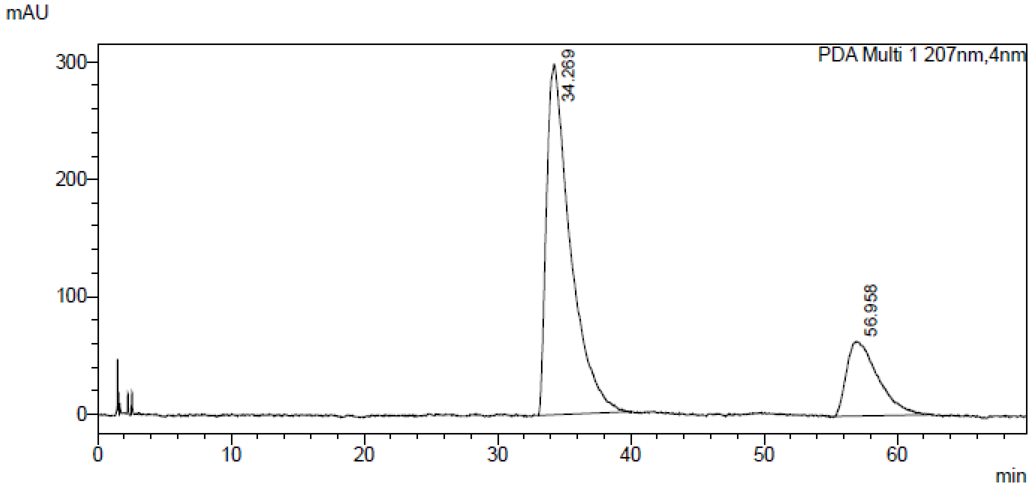

<Peak Table>

PDA Ch1 207nm

Peak#	Ret. Time	Area%
1	34.269	78.432
2	56.958	21.568
Total		100.000
